# Identification of Clinical Relevant Molecular Subtypes of Pheochromocytoma

**DOI:** 10.3389/fendo.2021.605797

**Published:** 2021-06-21

**Authors:** Umair Ali Khan Saddozai, Fengling Wang, Muhammad Usman Akbar, Lu Zhang, Yang An, Wan Zhu, Longxiang Xie, Yongqiang Li, Xinying Ji, Xiangqian Guo

**Affiliations:** ^1^ Department of Preventive Medicine, Institute of Biomedical Informatics, Cell Signal Transduction Laboratory, Bioinformatics Center, School of Basic Medical Sciences, Henan University, Kaifeng, China; ^2^ Gomal Center of Biochemistry and Biotechnology, Gomal University, Dera Ismail Khan, Pakistan; ^3^ Department of Anesthesia, Stanford University, Stanford, CA, United States

**Keywords:** pheochromocytoma, prognosis, molecular subtype, mutation, subtype specific treatment

## Abstract

Pheochromocytoma (PCC) is a rare neuroendocrine tumor of the adrenal gland with a high rate of mortality if diagnosed at a late stage. Common symptoms of pheochromocytoma include headache, anxiety, palpitation, and diaphoresis. Different treatments are under observation for PCC but there is still no effective treatment option. Recently, the gene expression profiling of various tumors has provided new subtype-specific options for targeted therapies. In this study, using data sets from TCGA and the GSE19422 cohorts, we identified two distinct PCC subtypes with distinct gene expression patterns. Genes enriched in Subtype I PCCs were involved in the dopaminergic synapse, nicotine addiction, and long-term depression pathways, while genes enriched in subtype II PCCs were involved in protein digestion and absorption, vascular smooth muscle contraction, and ECM receptor interaction pathways. We further identified subtype specific genes such as *ALK, IGF1R, RET*, and *RSPO2* for subtype I and *EGFR, ESR1*, and *SMO* for subtype II, the overexpression of which led to cell invasion and tumorigenesis. These genes identified in the present research may serve as potential subtype-specific therapeutic targets to understand the underlying mechanisms of tumorigenesis. Our findings may further guide towards the development of targeted therapies and potential molecular biomarkers against PCC.

## Introduction

Pheochromocytoma (PCC) is a type of tumor with catecholamine secretion derived from the chromaffin cells of the sympathoadrenal system (1–4). The majority of PCC arises within the adrenal medulla where the chromaffin cells are located in abundance (4). However, a small number of them are found in extra-adrenal sites (such as neck, mediastinum, abdomen, pelvis, and organ of Zuckerkandl) and are termed Paragangliomas (4, 5). The annual incidence rate of PCC is 1-4/10^6^ of the population while the recurrence rate is 4.6-6.5% (5, 6). In females the tendency for PCC progression (55.2%) is slightly higher than in males (44.8%). PCC occurs most frequently in aged individuals around 40-50 years old (7, 8). The most common signs and symptoms include hypertension, palpitation, headache, pallor, and sweating because of excessive catecholamine secretion. While the less common signs and symptoms are fever, nausea, weight loss, constipation, flushing, and fatigue (9). The metastases rate is about 10-15%in pheochromocytoma patients (10), but only a few patients are suitable candidates for surgical resection of the tumor in this case (11). Although the survival advantage of surgical debunking is not proven, it can significantly reduce organ damage, catecholamine secretion, and the required dosage of alpha and beta blockades. The decreased tumor burden as a result of surgical resection can also assist in successive radiotherapy or chemotherapy (9). Different methodologies are under process for guiding the treatment of cancers including the recently developed gene expression profiling methods used against gastric cancer, breast cancer, and uterine carcinosarcomas (12–18). The successful categorization of cancers into different molecular subtypes help cancer patients to receive better diagnosis and get more effective therapy for cancers (19). Therefore, the characterization of PCC into molecular subgroups will provide a better understanding of the underlying mechanisms of disease and thus will lead to a better and more precise treatment for PCC in the future. In the current study, by using gene expression profiling method, we successfully defined two distinct solid subtypes of PCC with enriched different potential genes and pathways. Our findings will accelerate the understanding of PCC pathogenesis and provide opportunities for effective subtype-specific therapies.

## Materials and Methods

### Determination and Validation of Molecular Subtypes of PCC

TCGA and Gene Expression Omnibus (GEO) databases were checked to obtain the Expression profiling data of clinical PCC cases. Two datasets, including one dataset from TCGA (154 cases) and the other dataset of GSE19422 from GEO (63 samples), were collected and used to define the molecular subtypes of PCC. After filtering individual expression datasets with standard deviation, the transformation of the data was done by gene-based centering. To identify the molecular subtypes, both datasets were separately run on Consensus clustering (R package Consensus clustering Plus) (20) with a set of parameters, including 80% sample resampling, distance (1-Pearson correlation), 80% gene resampling, maximum evaluated k of 12, agglomerative hierarchical clustering algorithm, and 1000 iterations. Finally, the R package cluster (silhouette width) was used to determine the accuracy of subtype assignment from Consensus Clustering Plus (21).

### Reproducibility Measurement of PCC Molecular Subtypes

Subclass Mapping (SubMap) implemented in Gene Pattern was used to determine the reproducibility of PCC molecular subtypes between TCGA and GSE19422 cohorts. SubMap analysis was achieved with parameters of (num. marker. genes=300, num.perm=1000 and num.per.fisher=1000) (22).

### Gene Ontology and Gene Set Enrichment (GSEA) Analysis

Subtype-specific genes were identified by SAM (23) and SAMseq (24) with a false discovery rate of less than 0.05. GO and KEGG pathway analyses were performed using DAVID Bioinformatics resources online version 6.7 (https://david.ncifcrf.gov/). GSEA (25) analysis was carried out to examine the expression of gene patterns and pathways of each subtype. Furthermore, therapeutic genes of each PCC subtype were explored through the TARGET V2 database (http://www.broadinstitute.org/cancer/cga/target).

### Statistical Analysis

For the evaluation of statistical significance between the clinical factors and subtypes of PCC, Fisher exact tests and chi-square test were applied and a *p-value* value less than 0.05 was considered to be significant. The survival curve was also calculated by log-rank test and Kaplan-Meier plot through Graphpad Prism 7 software.

## Results

### Consensus Clustering Identifies Two Different PCC Molecular Subtypes

**Figure 1:**
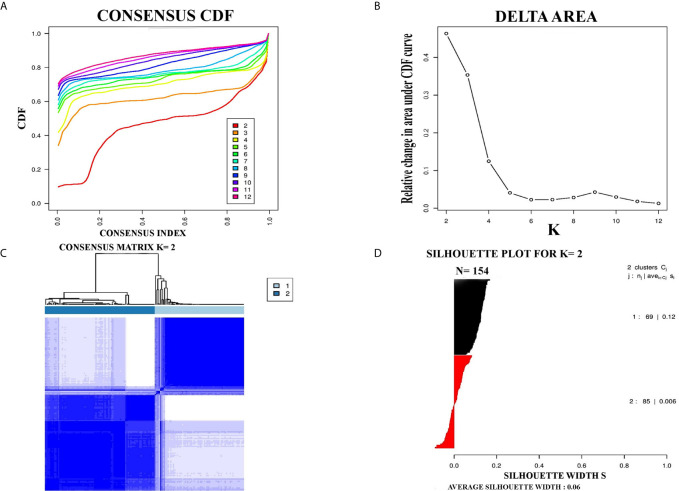
Represented two molecular subtypes in TCGA cohort of PCC. **(A)** The optimal number of PCC molecular subtypes was determined through Empirical cumulative distribution plot. **(B)** The increasement of area under the CDF curve with the increased expected number of molecular subtypes. **(C)** Consensus clustering matrix for the two distinct subtypes of PCC. **(D)** Silhouette plot based on Consensus clustering assignment.

PCC subtypes were identified using the gene expression profiling data of PCC with consensus clustering. Initially, the TCGA cohort (154 PCC samples) was revealed to have two optimal molecular subtypes based on the curve of empirical cumulative distribution (CDF) (**Figures 1A–C**). Subtype assignment was confirmed through silhouette width analysis. Out of 154 samples, 114 samples were found to have positive silhouette value, which was used for further analysis. In 114 samples, subtype I gathered 69 samples while 45 samples belonged to subtype II (**Figure 1D**).

### Further Validation of PCC Molecular Subtypes in an Independent Dataset

**Figure 2:**
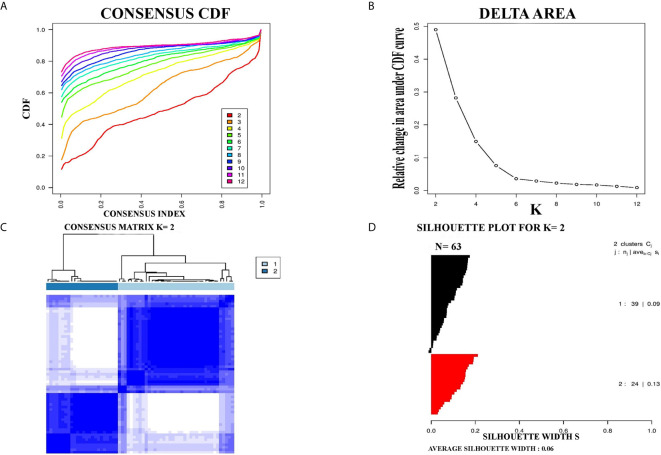
Identification of two molecular subtypes in GSE19422 cohort of PCC. **(A)** The optimal number of PCC molecular subtypes was defined through Empirical cumulative plot. **(B)** Comparative increase in the area under the CDF curve with the increasing expected number of molecular subtypes. **(C)** Consensus clustering matrix of the two PCC subtypes. **(D)** Silhouette plot of PCC samples based on Consensus clustering assignment.

For further confirmation of PCC subtypes identified in the TCGA cohort, a GEO dataset (GSE19422) with 63 PCC cases was analyzed. Consensus clustering identified two distinct molecular subtypes in the GSE19422 dataset as well (**Figure 2**). As in the TCGA dataset, positive silhouette cases were obtained and used for further analysis in GSE19422.

### Reproduced Molecular Subtypes in Independent PCC Cohorts by SubMap Analysis

**Figure 3:**
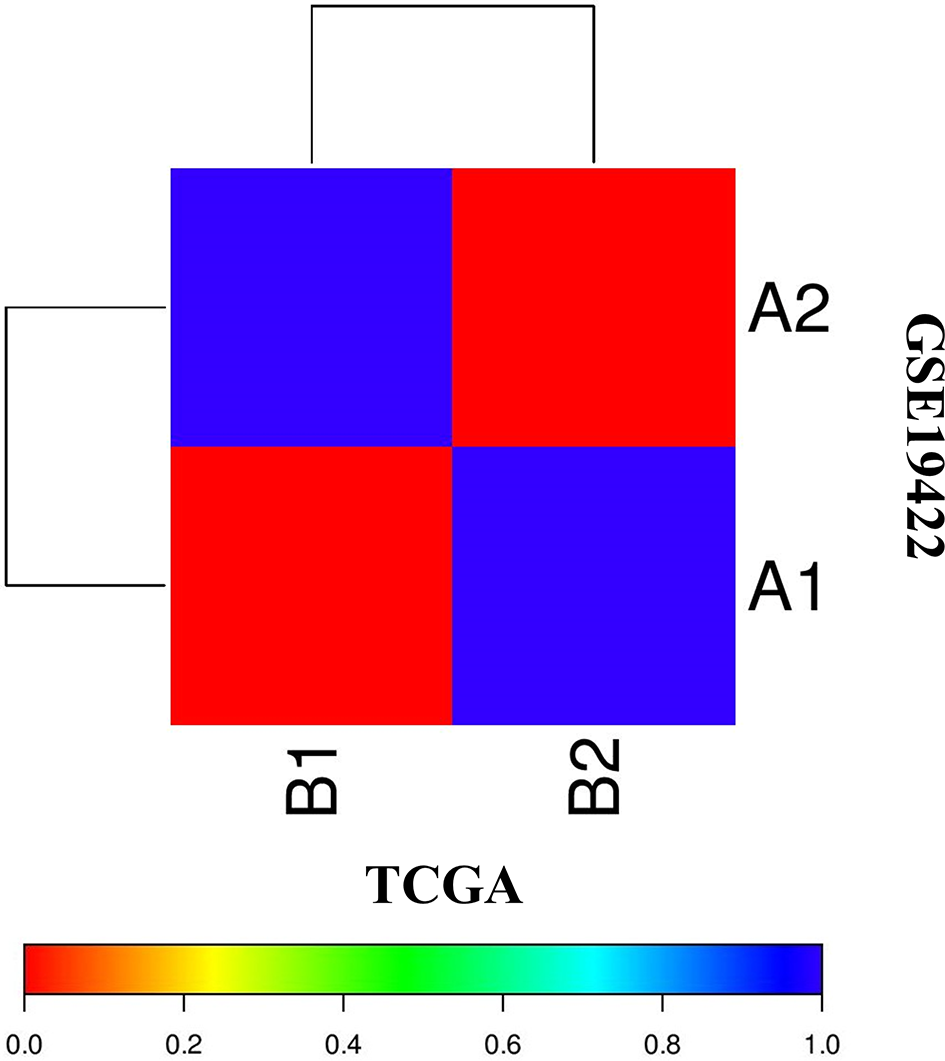
SubMap association between the two molecular subtypes from the two independent datasets of TCGA and GSE19422 presents a significant correlation. FDR-corrected p-value denoted the correlation significance.

The correlation of two distinct molecular subtypes of PCC in independent datasets was measured through SubMap analysis. The result of SubMap analysis revealed a significant correlation between A1-A2 subtypes of TCGA with the B1-B2 of GSE19422 (**Figure 3**), indicating the common and reproducible PCC molecular subtype across different cohorts.

### Clinical Characteristics of PCC Molecular Subtypes

To understand the clinical characteristics of PCC molecular subtypes, the relationship between molecular subtypes and clinical factors was checked in the TCGA cohort. Notably, the laterality rate of subtype II PCC was found to be higher on the left side of patients (24/45) than subtype I PCC (34/69) (**Supplementary Table S1**, *P* = 0.2057). Furthermore, it is noteworthy to highlight that the detection rate of the disease during the initial screening was found to be higher in subtype I PCC patients (37/69) than in subtype II PCC patients (22/45) (*P* = 0.5883). The mean age at diagnosis was found to be higher in Subtype I PCCs (48.4 years) as compared to Subtype II PCCs (41.8 years) (*P*=0.02*). The median overall survival (OS) time in patients of Subtype I PCCs was recorded as 736 days, which was slightly shorter than the patients with Subtype II PCCs who had an OS time period of 944 days. However, survival curve analysis (Kaplan-Meier plots) showed no significant difference in survival between the two PCC subtypes. In addition to the survival rate, there was no significant difference found based on the patient’s sex among the two subtypes (*P* = .5248) as well as their success in primary therapy outcome (*P* = 0.6136) (**Supplementary Table S1**).

### Functional Analysis of PCC Subtype-Specific Genes

**Figure 4:**
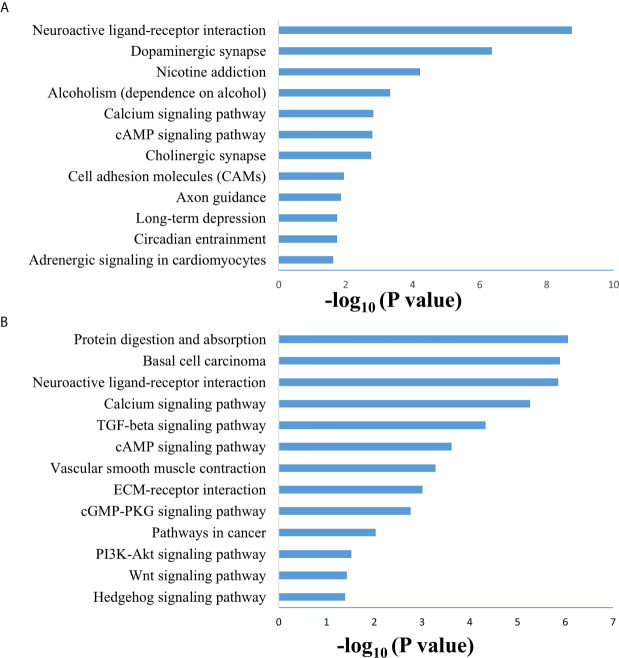
Enriched biological pathways found through the analysis of overexpression of genes individual PCC subtypes. **(A)** KEGG pathway through the gene overexpression profile analysis in subtype (I) **(B)** KEGG pathway through the gene overexpression profile analysis subtype II.

**Figure 5:**
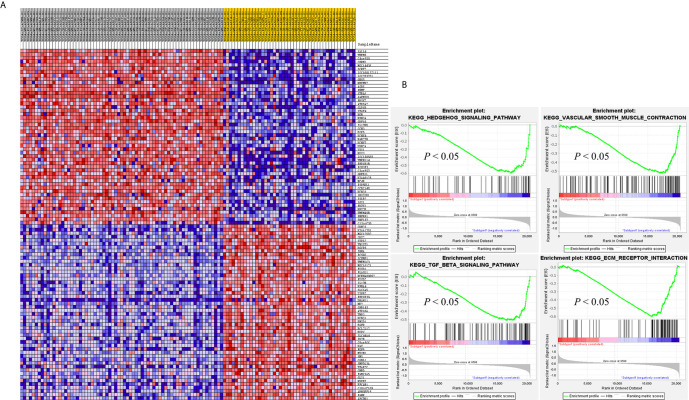
GSEA results validates the different gene expression patterns found in each PCC subtype. **(A)** Heatmap of gene expression of Top 100 genes in subtype I and subtype II. Over-expressed genes (Red) and down-expressed genes (blue). **(B)** GSEA enriched pathways in subtype II. GSEA results presented improved expression of vascular smooth muscle contraction and hedgehog signaling pathway. Distinct gene expression signatures are produced by the individual gene sets enriched in subtype I and subtype II. Permutation = 1000, *p* < 0.01.

SAMseq analysis was performed in the TCGA dataset to analyze differentially expressed genes between two PCC molecular subtypes. A total of 6813 genes were found to have differential expression between the subtypes, among which 2840 genes were overexpressed in subtype I while 3973 genes had higher expression in subtype II (**Supplementary Table S2**). KEGG and GO analyses were performed on the Top 1000 overexpressed genes from each PCC subtype to obtain further biological information about the subtypes. GO analysis revealed 187 biological processes enriched in subtype I, including Nervous system-associated genes (4.5%) (**Supplementary Table S3**). KEGG analysis of subtype I overexpressing genes revealed 23 different pathways that belonged to Neuroactive ligands receptor interaction, cAMP signaling pathways, and Calcium signaling pathways, (**Figure 4A**). Whereas 259 biological processes and 26 KEGG pathways were significantly enriched in subtype II PCCs. These pathways included Vascular Smooth Muscle Contraction, ECM Receptor Interaction, and Hedgehog Signaling Pathway. (**Figure 4B**). In addition, GSEA analysis in the TCGA cohort demonstrated gene sets enriched with significant biological pathways were found to be abundant only in subtype II. These pathways for subtype II include Hedgehog signaling pathways, Vascular smooth interaction, ECM interaction, and TGF Beta signaling pathway (**Figure 5**).

### Clinical Implication of PCC Subtyping

The purpose of molecular subtyping of PCC was to search and identify therapeutic routes and to employ those specified routes further in clinical studies and discourses. Overexpressed genes in each PCC subtype were obtained and compared with the target database (that contains target genes and functional inhibitors) for the determination of therapeutic molecules (26). Further studies may be carried out on the targeted genes to translate them into potential clinical stages (27–29).

We have found seven subtype-specific target genes which would give relative benefits to the PCC patients from distinct subtypes. Given in **Table 1**, subtype I PCCs benefit from four target genes, namely *ALK, IGF1R, RET*, and *RSPO2*, while subtype II contains three target genes: *EGFR, ESR1*, and *SMO*.

**Table 1:** Target genes enriched in each molecular subtype.

Gene overexpressed	Examples of Potential Therapeutic Agents	
	*ALK*	Crizotinib, ALK inhibitors
	*IGF1R*	IGF1R Inhibitor
**Subtype I**	*RET*	Sorafenib, vandetinib, RET Inhibitors
	*RSPO2*	WNT inhibitors
	*EGFR*	Erlotinib, Gefitinib, EGFR Inhibitors
**Subtype II**	*ESR1*	Hormonal therapy
	*SMO*	Vismodegib, hedgehog inhibitors

## Discussion

PCC is a type of catecholamine-secreting neuroendocrine tumors, most of which arise from chromaffin cells of the adrenal medulla. About 15-20% of these PCC tumors belong to an extra-adrenal origin and are termed as paraganglioma (PGL) (30). Due to excessive secretion of catecholamines, both forms of tumors (adrenal and extra-adrenal) have similar clinical symptoms and are only distinguished based on potential differences in prognosis (31). Most of the PCCs are benign, although metastasis may develop in patients with specific backgrounds (32, 33). At present, different studies have debated PCC treatment. Patients at the same stage may still respond to treatment differently due to the molecular heterogeneity even if they were administered the identical treatment (34, 35). Molecular subtyping approaches based on gene expression profiling of tumors has greatly guided the medical community in introducing subtype specific diagnostic techniques and targeted therapies (20). The subtype-based targeted therapies in clinical trials of breast cancer is a good example of molecular prognostic and treatment of malignancies. The positive response of HER2-positive breast cancer patients towards subtype specific therapies is an example for the future directions of the current study (36). Using the gene expression profiling method, it is possible to get a better understanding of the heterogeneity of PCCs, and also provides the opportunity to develop subtype-specific therapeutic strategies.

In this study, we identified two molecular subtypes of PCC (also confirmed previously by Fishbian et al.) (37). The Gene set enrichment and Gene ontology analyses of the identified subtypes revealed the overexpression of certain genes and pathways specific to each subtype (**Supplementary Table S4**). The subtype I PCCs include the overexpressed genes involved in pathways of Dopaminergic synapse, Nicotine addiction, and Long-term depression. The *SALL4* involved in the proliferation of cancer was also found to be overexpressed within subtype I (**Figure 5**). *SALL4* gene is involved in the maintenance of pluripotency and self-renewal of embryonic stem cells (38, 39). Expression of *SALL4* has been reported in various cancers such as precursor B-cell lymphoblastic lymphoma (40, 41), acute myeloid leukemia (42), myelodysplastic syndromes (43), breast cancer (44), chronic myeloid leukemia (45), lung cancer (46, 47), endometrial cancer (48), liver cancer (49, 50) gastrointestinal carcinoma (51–53), glioma (54), germ cell tumor, and yolk sac tumor (55, 56). In subtype II PCCs, enriched genes and pathways include the overexpression of smooth muscle-specific markers and the genes involved in the lymph node. Overexpressed pathways in subtype II include protein digestion and absorption pathway, Vascular smooth muscle contraction pathway, and ECM-receptor interaction pathway. The overexpressed gene in this subtype include *Twist1* (**Figure 5**). The consistent significance of *Twist1* gene in cancer biology involves its overexpression which is linked to metastasis, therapeutic failure, recurrence, and inferior prognosis (57). The role of *Twist1* has already been shown to serve as a useful prognostic factor predicting poor outcome in breast cancer (58), nasopharyngeal cancer (59), ovarian cancer (60) and cervical cancer (61). Expression of these distinct genes and pathways in each subtype will provide a better way to understand PCC at the subtype level and to develop subtype-specific treatment.

After analyzing the overexpressed genes and pathways in each subtype, we further checked these overexpressed genes in the TARGET database and identified seven known target genes for each subtype. Subtype I PCCs have four overexpressed genes, namely *ALK, IGF1R, RET*, and *RSPO2*, while subtype II includes *EGFR, ESR1*, and *SMO*. In most types of cancers, overexpression of the *IGF1R* gene is found to be a typical hallmark (62). In addition, *IGF1R* has an important role in invasion, metastasis, and angiogenesis (63–65). Its overexpression has also been found in pheochromocytoma and paraganglioma with a high risk of metastasis (66).

Blocking the IGF1R *via* antisense therapy (67), anti IGF1R antibodies (68–70), dominant negative IGF1R (71), and small-molecule inhibitors has proven efficacious in the treatment of various cancers. A preclinical study found Cixutumumad effective against prostate cancer as it caused significant delaying of the androgen resistance by blocking IGF1R in disease (72, 73). Recent studies have suggested linsitinib (OSI-906) as a promising drug for PCC patients, when used alone or combined with mTOR inhibitors (74). Therefore, IGF1R inhibitors may play a significant role in subtype I of PCC.

The overexpression of *EGFR* has been observed to play a key role in tumorigenesis (75). Targeting *EGFR* using different approaches has proven effective in the treatment of various solid tumors such as head and neck, colorectal, pancreatic, and non-small lung cancer (NSCLS) (76–80). Gefitinib, the first FDA-approved anti-EGFR drug (81, 82), has been shown to prevent autophosphorylation of *EGFR* in many tumor cell lines and xenografts (83). It inhibits the cell growth in HER2-overexpressing breast cancer cells (84, 85). Similarly, Erlotinib is another FDA-approved drug that acts as an inhibitor of *EGFR* (86, 87) and has been proven effective in the treatment of NSCLC and metastatic pancreatic cancer when used in combination with gemcitabine (76, 80). Gene expression analysis revealed the role of the *EGFR* gene in subtype II patients of the PCC cohort. Based on the *EGFR’s* role in cancer and the availability of anti-EGFR inhibitors, patients of subtype II may benefit from anti-EGFR inhibitors.

In conclusion, we have characterized two distinct molecular subtypes of PCC in two independent cohorts. Differentially expressed genes found in the two subtypes provide an insight into the underlying mechanisms of tumorigenesis and progression in a subtype-specific manner. Targeted therapies against molecular targets identified in the present study may help better understand the disease prognosis and aid in developing specified therapies against individual subtypes of PCC.

## Data Availability Statement

The datasets presented in this study can be found in online repositories. The names of the repository/repositories and accession number(s) can be found in the article/**Supplementary Material**.

## Author Contributions

Term Definition Conceptualization: XG. Methodology: XG. Software: XG. Validation: US, XG. Formal analysis: US, XG, FW, ZL. Investigation: US, XG. Resources: XG, XJ, YL. Data Curation: US, XG. Writing - Original Draft: US, MA, WZ, XG. Writing - Review and Editing: US, MA, WZ, XG, XJ, LX, YA. Visualization: US. Supervision: XG, XJ, YL. Project administration: XG, XJ, YL. Funding acquisition: XG. All authors contributed to the article and approved the submitted version.

## Funding

This work was supported by the program for Innovative Talents of Science and Technology in Henan Province (No. 18HASTIT048). The funding bodies were not involved in the study design, data collection, analysis and interpretation of data, or writing of this manuscript.

## Conflict of Interest

The authors declare that the research was conducted in the absence of any commercial or financial relationships that could be construed as a potential conflict of interest.
