# Supplementary material for: Identification of Clinical Relevant Molecular Subtypes of Pheochromocytoma
Source: Front Endocrinol (Lausanne). 2021 Jun 21;12:605797. doi: 10.3389/fendo.2021.605797 (PMC8256389; doi:10.3389/fendo.2021.605797)
Supplement: Supplementary file 1 [file DataSheet_1.pdf]

Table 1. Clinicopathologic Characteristics (N=154)

Characteristic	Patients, n(%)	Subtype I	Subtype II	Others	*P value*
**Total number**	154	69	45	40	
**Age (year)**					
Mean	47	48.4	41.8	50.45	0.02*
Range	19-83	19-83	19-78	28-80	
**Sex**					
Female	86	40	22	24	0.5248
Male	68	29	23	16	
**Laterality**					
Left	73	34	24	15	0.2057
Right	79	35	19	25	
Bilateral	1	0	1	0	
**Disease detected on screening**					
Yes	79	37	22	20	0.5883
No	36	19	11	6	
**Benign Tumor**					
Yes	16	5	10	1	0.0095*
No	138	64	35	39	
**Primary therapy outcome success**					
Progressive diseas	7	2	3	2	0.6136
Complete respons	139	65	39	35	
**Radiation therapy**					
Yes	2	1	1	0	1
No	149	67	43	39	
**Time to event (days)**					
Median	78	736	944	691	0.097
Range	2--4185	2--3491	43-4185	24-3293	
**Event**					
Alive	148	65	44	39	1
Dead	4	2	1	1	
