# Supplementary material for: Identification of Clinical Relevant Molecular Subtypes of Pheochromocytoma
Source: Front Endocrinol (Lausanne). 2021 Jun 21;12:605797. doi: 10.3389/fendo.2021.605797 (PMC8256389; doi:10.3389/fendo.2021.605797)
Supplement: Supplementary file 2 [file DataSheet_2.pdf]

Table 2. Results of SAM analysis between different subtypes of PCC in TCGA.
Data is representing overexpressionof gene in two subtype in positive and minus value.
(1).Gene were ordered according the SAM significance, gene with the positive value were
overexpressed in subtype II of PCC whereas minus value genes were overexpressed in subtype I.
(2).Significance increased with decreased of absolute values of ranks from 10 to 1 or -10-1.Rank 1 or -1 genes are the most significant of SAM results.

Gene symbol	TCGA sItosII	Gene symbol	TCGA sItosII	Gene symbol	TCGA sItosII	Gene symbol	TCGA sItosII	Gene symbol	TCGA sItosII
NHEG1	-1768	LOC285593	-609	PALMD	551	ADAMTSL2	1710	CAPN8	2869
CD1E	-1767	CILP2	-608	ADAMTS6	552	NAV3	1711		
ITPR2	-1766	AGRP	-607	CERK	553	MYC	1712		
FAM157A	-1765	RNF157	-606	TDRD10	554	PQLC3	1713		
CPNE9	-1764	DEFB132	-605	DACH2	555	HP	1714		
FLJ25758	-1763	FAM196B	-604	EVX1	556	SLC5A12	1715		
GLUD2	-1762	C14orf39	-603	IL1R1	557	PLOD2	1716		
DZIP1	-1761	MTMR8	-602	TMEM102	558	GPAT2	1717		
C16orf68	-1760	C19orf30	-601	MSX1	559	MYO1D	1718		
PDE4DIP	-1759	SPOCK1	-600	FAM69C	560	PDK1	1719		
NPM2	-1758	CD226	-599	RCC1	561	RHCG	1720		
PCDHA10	-1757	TCF7	-598	PDE1B	562	SOX18	1721		
TIGIT	-1756	GPLD1	-597	C8orf85	563	C8orf84	1722		
NBLA00301	-1755	MORN3	-596	UPB1	564	LOC728875	1723		
CCDC136	-1754	C20orf201	-595	DENND2A	565	NXNL1	1724		
TTLL7	-1753	GPR75	-594	RGMA	566	CLEC14A	1725		
LOC349196	-1752	IGF1R	-593	GGT3P	567	SPARCL1	1726		
LOC113230	-1751	ZNF826	-592	LAYN	568	B4GALT1	1727		
SNORD115-13	-1750	CHRM4	-591	MAP1LC3C	569	LMO3	1728		
BEX2	-1749	HMX3	-590	POU6F2	570	RHBDF1	1729		
F13A1	-1748	FMNL1	-589	ASPN	571	VEGFC	1730		
XCL1	-1747	LCP1	-588	GLI2	572	LOC729991-ME	1731		
PGBD1	-1746	KNCN	-587	CRHR1	573	PLAC8L1	1732		
HARBI1	-1745	CADM4	-586	SLC17A7	574	LOXL2	1733		
LOC100272216	-1744	C15orf59	-585	GRAMD3	575	TCFL5	1734		
STH	-1743	PCDHGC5	-584	CP	576	CD209	1735		
CEP170	-1742	DNAI1	-583	ZNF114	577	H1FNT	1736		
SLC4A9	-1741	PCDHGB8P	-582	C14orf181	578	KCTD11	1737		
OXCT1	-1740	ARSF	-581	KRT7	579	NOTCH2	1738		
C1orf21	-1739	DKK3	-580	SLC2A1	580	LMOD1	1739		
ASXL2	-1738	TEX19	-579	LAMC3	581	SCARF2	1740		
LNPEP	-1737	PSG1	-578	DLX6	582	IL3RA	1741		
FAM150B	-1736	KIF21A	-577	PABPN1L	583	SLC19A3	1742		
HCN3	-1735	DNASE2B	-576	MBOAT1	584	C1orf105	1743		
CCDC121	-1734	NPB	-575	CABP1	585	SAA4	1744		
RIN1	-1733	ACTA1	-574	ADA	586	GRID2IP	1745		
HIST1H2BN	-1732	CCL11	-573	BEND4	587	NEUROD4	1746		
C15orf62	-1731	HYDIN	-572	CCDC102A	588	CPA6	1747		
TBC1D9	-1730	IFIT3	-571	SCUBE3	589	SULT2A1	1748		
PTK7	-1729	CHD5	-570	RCVRN	590	LOC100101266	1749		
PLA2G2D	-1728	C6orf114	-569	LRRC8E	591	SYT15	1750		
NAALAD2	-1727	TRIL	-568	ADAD2	592	MMP12	1751		
ELN	-1726	SCN5A	-567	ITGA8	593	ALG3	1752		
ATG12	-1725	HHAT	-566	RARRES2	594	NCRNA00173	1753		
ELL2	-1724	FLJ40330	-565	KIF25	595	PDE5A	1754		
LBX1	-1723	SPAG6	-564	BARX1	596	PPAP2B	1755		
C1orf173	-1722	SYNGR3	-563	RBPMS	597	ABCA8	1756		
FAM57B	-1721	ELFN2	-562	ART5	598	LRRC10B	1757		
SLC25A45	-1720	CECR2	-561	MMRN1	599	RIN3	1758		
PCDHGA10	-1719	KCNH4	-560	EDARADD	600	S100A7	1759		
IL15	-1718	LOC647859	-559	CDC42BPG	601	PVALB	1760		
C18orf8	-1717	EML6	-558	EMP3	602	PART1	1761		
ROGDI	-1716	MYCBPAP	-557	GRIN2C	603	PKHD1L1	1762		
CD200	-1715	DQX1	-556	PLCH1	604	KCNMB3	1763		
TMEM151A	-1714	KLHL33	-555	SLC2A14	605	NLRP1	1764		
TUBB2B	-1713	ARHGAP22	-554	SGCA	606	SIRT4	1765		
DPYSL4	-1712	LOC642597	-553	PLAC9	607	FAM3B	1766		
VSTM2L	-1711	AK7	-552	SLC12A8	608	TFPI	1767		
BAIAP3	-1710	PDYN	-551	CRHBP	609	FBXO39	1768		
FAM154B	-1709	DPY19L2P2	-550	AANAT	610	C20orf106	1769		
ATE1	-1708	SH3D20	-549	GPRC6A	611	NR2E1	1770		
LOC149837	-1707	GLYATL2	-548	CLCN1	612	TMEM51	1771		
C10orf35	-1706	GDF15	-547	KCNK5	613	TNFRSF10B	1772		
APLP1	-1705	ARHGAP26	-546	TBC1D26	614	FXYD1	1773		
MBL2	-1704	ZCCHC18	-545	ALPL	615	IQGAP2	1774		
C17orf46	-1703	LOC100130148	-544	LOC285954	616	EPHA7	1775		
LPIN1	-1702	PPFIBP2	-543	TPSAB1	617	FLT4	1776		
C20orf144	-1701	SYN2	-542	MAG	618	EFHB	1777		
B3GALT1	-1700	GPR137C	-541	MRAP	619	SLC6A6	1778		
DLX2	-1699	ADAM12	-540	KCNF1	620	OSMR	1779		
FAM7A3	-1698	SYT2	-539	FGF13	621	C2orf89	1780		
TRAM1L1	-1697	PDIA2	-538	IFFO2	622	C6orf221	1781		
MAPK8IP1	-1696	YPEL4	-537	FMO4	623	PLS1	1782		
SLC27A2	-1695	OR1J2	-536	GRM5	624	WDR93	1783		
BHLHB9	-1694	DNAH10	-535	TCF21	625	INMT	1784		
MOBP	-1693	C11orf41	-534	GJA1	626	CALML6	1785		
ACE	-1692	KCNG1	-533	KHDRBS2	627	PRRX1	1786		
UAP1L1	-1691	NHLH1	-532	C6orf123	628	ACPP	1787		
TNIK	-1690	GAP43	-531	NDRG1	629	FAM81B	1788		
TMX4	-1689	GULP1	-530	SYCP2L	630	OR51E1	1789		
CKB	-1688	XYLB	-529	WWC1	631	SLC22A2	1790		
AGPHD1	-1687	ARHGEF4	-528	CBLN4	632	IGFBP6	1791		
ARHGAP27	-1686	DPY19L2P1	-527	TPRXL	633	HRK	1792		

HELB	-1685	C7orf57	-526	C3orf36	634	RHOBTB1	1793
FAM164C	-1684	TRIM14	-525	C15orf60	635	FLI1	1794
ABAT	-1683	OAS3	-524	PPP1R14A	636	CCDC42B	1795
UNC80	-1682	RENBP	-523	ZNF469	637	ILDR1	1796
RIMS1	-1681	CORO6	-522	PLEKHH2	638	ZNF238	1797
KIF3C	-1680	ACSL6	-521	KCNJ15	639	GNGT1	1798
CCDC75	-1679	RNASE12	-520	JPH2	640	RERG	1799
PRRT4	-1678	WBSCR26	-519	C21orf15	641	ST7OT1	1800
GPATCH2	-1677	TCEAL6	-518	SOX21	642	TSPAN2	1801
ZFP92	-1676	SNORD115-26	-517	MYL3	643	LDLRAD1	1802
FAM155B	-1675	STEAP2	-516	MAFA	644	NXF3	1803
DNASE1L2	-1674	HKDC1	-515	LOC150197	645	LOH3CR2A	1804
C11orf75	-1673	TMEM59L	-514	TRPA1	646	CYP3A7	1805
TIFAB	-1672	CDK15	-513	NR0B1	647	GPRC5B	1806
RPS10P7	-1671	RASAL1	-512	ACTL8	648	NOTCH2NL	1807
C3orf67	-1670	DRP2	-511	EN2	649	NNMT	1808
C4orf35	-1669	ZNF391	-510	MT1L	650	LYPLA2P1	1809
ST7OT3	-1668	NRK	-509	RSPO1	651	C19orf59	1810
CNTN1	-1667	LRRC16B	-508	IL1RL2	652	GYG2	1811
SP110	-1666	SIAE	-507	HRC	653	MAMDC2	1812
C10orf75	-1665	PRDM8	-506	ACSBG1	654	MGLL	1813
OSBPL3	-1664	LOC148696	-505	PCDH19	655	C19orf18	1814
TMEFF1	-1663	LONRF3	-504	FBXO40	656	FOXC1	1815
SPDYE1	-1662	NDST3	-503	FOSL2	657	IMPA2	1816
STK32A	-1661	UCN3	-502	SLC10A6	658	CALCRL	1817
MAGEE1	-1660	DKFZP434L187	-501	NOSTRIN	659	ITGA11	1818
LOC442421	-1659	ELAVL4	-500	PDE1A	660	HMGB3	1819
FOXG1	-1658	HIST3H2BB	-499	IRS1	661	IGFBP7	1820
MAML2	-1657	SLC4A4	-498	STC2	662	TGIF1	1821
C12orf66	-1656	CEND1	-497	PEX5L	663	C8orf22	1822
ICAM5	-1655	CCNA1	-496	FXYD2	664	TIE1	1823
KIAA1377	-1654	CACNG4	-495	CNTN4	665	DHRS7B	1824
SPTBN4	-1653	C6orf227	-494	ADORA2A	666	GJC1	1825
CRMP1	-1652	SNTG1	-493	C16orf74	667	TM4SF4	1826
EEF1DP3	-1651	SLC6A2	-492	MYOZ3	668	TRIM54	1827
FAM90A1	-1650	CRYBA2	-491	SLC17A3	669	RPS8	1828
PCDHGB5	-1649	MDK	-490	XPNPEP2	670	NRARP	1829
ZNF195	-1648	PENK	-489	CA3	671	NFIA	1830
ZNF821	-1647	CRIP3	-488	ZNF385D	672	AOC2	1831
PCDHGB4	-1646	C19orf21	-487	ST8SIA6	673	LSP1	1832
CXCL10	-1645	CHST11	-486	GEMIN8P4	674	HDAC7	1833
LOC100130872	-1644	BEGAIN	-485	PTX4	675	HOXB9	1834
CPEB2	-1643	ASAH2B	-484	FOLH1	676	PABPC4L	1835
PTPLAD1	-1642	GAL	-483	VSIG1	677	IL23R	1836
GPC2	-1641	LRRC3B	-482	CHAD	678	GJA3	1837
PTCD2	-1640	NPW	-481	ZFPM2	679	ERRFI1	1838
REEP2	-1639	B3GNT3	-480	CYP11B2	680	IQCG	1839
KHK	-1638	B3GNT4	-479	SATB2	681	FHL2	1840
HIST1H4H	-1637	HOXC10	-478	PHGDH	682	CDCP2	1841
SBNO1	-1636	FAM86B2	-477	HRNBP3	683	GPR4	1842
F10	-1635	ALS2CR11	-476	TMEM200A	684	EBF3	1843
C11orf70	-1634	ZDHHC8P1	-475	CHRND	685	NEB	1844
HCG4	-1633	MAP2	-474	HRCT1	686	SHISA6	1845
EXOC8	-1632	PEBP4	-473	PTGFRN	687	C6orf145	1846
CASKIN1	-1631	GLT1D1	-472	ISM1	688	UCN	1847
OGFRL1	-1630	GPR1	-471	KLK2	689	TEAD4	1848
NAA15	-1629	DENND1B	-470	CCDC36	690	C21orf7	1849
DPY19L3	-1628	ANKRD34C	-469	FRK	691	LOC254559	1850
DOCK11	-1627	CAMK4	-468	C1QTNF2	692	ADCY5	1851
SLC1A2	-1626	NNAT	-467	IGDCC3	693	C14orf70	1852
ZDHHC22	-1625	CCL22	-466	LOC100190938	694	STC1	1853
F12	-1624	PCDHAC2	-465	LRMP	695	RTN4RL2	1854
NEFH	-1623	LAG3	-464	GPC4	696	SNORD17	1855
NOG	-1622	CPNE7	-463	EFHD1	697	SERINC2	1856
CHST15	-1621	PTGER3	-462	SVOPL	698	TDRD5	1857
LRRTM2	-1620	MEG8	-461	CSMD2	699	GPR44	1858
WDR17	-1619	FLRT1	-460	PHACTR2	700	ORC1L	1859
SLC25A40	-1618	TCHP	-459	PCK1	701	CD248	1860
BLZF1	-1617	DMC1	-458	ROBO3	702	RNASE10	1861
CDH11	-1616	PCSK2	-457	SEC14L4	703	FAM194A	1862
CCDC34	-1615	NELL1	-456	RHCE	704	EDN3	1863
THEMIS	-1614	PCDH10	-455	MGST1	705	NRG4	1864
MEGF11	-1613	FLG	-454	SLC24A3	706	OBP2B	1865
RABIF	-1612	TMEM100	-453	ALOX15B	707	ACE2	1866
LOC283174	-1611	HTN3	-452	ATOH8	708	IGF2BP1	1867
CHRNA7	-1610	ZNF215	-451	KLHL32	709	SHE	1868
GNG2	-1609	TM6SF2	-450	EPHX1	710	LTBP1	1869
LY6G6C	-1608	NHSL2	-449	CFC1B	711	KCNK6	1870
INSM2	-1607	CCDC78	-448	PPYR1	712	F3	1871
IGSF11	-1606	TRPC7	-447	IRX4	713	SPAG17	1872
SH3RF1	-1605	FGF16	-446	SLC25A34	714	PGF	1873
MYD88	-1604	MAB21L2	-445	C10orf41	715	ANO1	1874
PCDHGB2	-1603	MYOZ2	-444	FXYD5	716	GRAMD1C	1875
SLC8A3	-1602	GPA33	-443	CYS1	717	KLHL1	1876
KIAA1949	-1601	EPHA6	-442	DACT1	718	SLC15A2	1877
CCL20	-1600	DUSP4	-441	LOC389634	719	CBY1	1878
FNDC3A	-1599	DBC1	-440	RAB34	720	ENPEP	1879
ANKRD20B	-1598	ABCG4	-439	TRPC4	721	RGS20	1880
GLRX	-1597	HERC6	-438	CDX1	722	ASGR2	1881

ZNF578	-1596	FSIP1	-437	GNASAS	723	LOC283050	1882
GRIP1	-1595	PRRG4	-436	FLJ40852	724	LOC728855	1883
SNX32	-1594	ADAM22	-435	LYVE1	725	BNC1	1884
STS	-1593	FAM83G	-434	AREG	726	NFIL3	1885
TRIM36	-1592	NRG1	-433	COL4A4	727	FZD8	1886
HCN1	-1591	TUBBP5	-432	MYH3	728	TTLL10	1887
C2CD2L	-1590	STX1B	-431	USH1G	729	C17orf72	1888
C16orf3	-1589	NPTX1	-430	MT1A	730	ICOSLG	1889
CD27	-1588	SHD	-429	UNC45B	731	GPR12	1890
APC2	-1587	DISP2	-428	ACSF2	732	CNO	1891
FMN2	-1586	C11orf45	-427	CLEC4M	733	ZYX	1892
C7	-1585	CDH10	-426	DUOXA1	734	KLHDC8A	1893
PTPN20B	-1584	CAPN6	-425	HTR1D	735	KRT40	1894
ALS2CR12	-1583	SLC38A11	-424	SLC28A1	736	ENG	1895
ENPP6	-1582	SLC26A5	-423	PTPN14	737	MYO7B	1896
LOC348926	-1581	ANKRD33	-422	FBP2	738	CLEC18B	1897
PTK2B	-1580	DGAT2	-421	TACSTD2	739	CD34	1898
INTS4L1	-1579	TMED7-TICAM2	-420	CCBP2	740	MYLK2	1899
GLRA2	-1578	RCOR2	-419	DES	741	DMBX1	1900
SEMA6D	-1577	SERTAD4	-418	GSTA3	742	SLCO2A1	1901
LOC284441	-1576	SCN8A	-417	LDB2	743	C21orf91	1902
SNAP91	-1575	SRMS	-416	CCDC19	744	KRTAP19-1	1903
LOC285847	-1574	IL7	-415	AFARP1	745	CKM	1904
SMN1	-1573	CDKL5	-414	KRT14	746	PF4	1905
CLOCK	-1572	C13orf36	-413	GSTM5	747	DMP1	1906
C5orf20	-1571	RNF175	-412	SPINK2	748	GP2	1907
ACTN1	-1570	HRH1	-411	C14orf73	749	HOXD9	1908
CELF4	-1569	GPD1	-410	VWC2	750	NOX5	1909
TBX20	-1568	MX1	-409	C1QTNF1	751	ALPK1	1910
LRDD	-1567	EIF4E3	-408	SLC6A15	752	EGLN3	1911
SULT1C2	-1566	AFF3	-407	EPO	753	DAB2	1912
ZNF154	-1565	SUSD5	-406	FGF11	754	ZNF169	1913
TMEM35	-1564	HS3ST2	-405	FAM110B	755	KLRG2	1914
ACCN1	-1563	CHRM5	-404	C8orf86	756	STX11	1915
ATP6V1G2	-1562	ANGPTL7	-403	ENTPD8	757	LMO2	1916
INTS7	-1561	CHRM3	-402	MYOCD	758	CPA2	1917
GPR174	-1560	GDEP	-401	IGFBP1	759	VEGFA	1918
HIVEP1	-1559	ZCCHC12	-400	ANO7	760	HEY2	1919
LOC440905	-1558	PPP4R4	-399	WNT9B	761	NAT8B	1920
NAIP	-1557	KRTAP5-1	-398	CPAMD8	762	ACSL5	1921
ST18	-1556	LOC283856	-397	C11orf9	763	CDC7	1922
PNMA1	-1555	UNC5C	-396	TMEM47	764	EMP1	1923
SVIP	-1554	OR2AG2	-395	ADAMTSL5	765	AHR	1924
WBP2NL	-1553	C12orf50	-394	KRT18	766	ZNF492	1925
GPR173	-1552	C11orf87	-393	LYPD3	767	CSPG4	1926
PRRT2	-1551	ATP2B2	-392	LOC572558	768	HSD17B3	1927
PSD	-1550	NUDT10	-391	REP15	769	GMFG	1928
AGTPBP1	-1549	TIGD4	-390	LPL	770	ST6GALNAC2	1929
MAP1A	-1548	LRRC4C	-389	FOXI2	771	COPZ2	1930
IFIT2	-1547	CSGALNACT1	-388	CNTNAP4	772	APOBEC3B	1931
RDH10	-1546	TRIM46	-387	TUSC5	773	MLKL	1932
BCL2L10	-1545	LOC100189589	-386	MCTP2	774	IRAK2	1933
SH2D6	-1544	ANKRD45	-385	PLA2G2A	775	LOC100190939	1934
C7orf52	-1543	SORCS1	-384	SOX11	776	C9orf70	1935
PTGER4	-1542	KCNT1	-383	CKMT2	777	GPX7	1936
GTF2H2B	-1541	OTOP1	-382	FABP7	778	SPC25	1937
TMEM151B	-1540	CYB5R2	-381	CRB1	779	SLC24A6	1938
RAPGEF6	-1539	ACTBL2	-380	SRL	780	CCDC37	1939
CDK14	-1538	AKR1C2	-379	ADORA2B	781	CHRNB1	1940
MC3R	-1537	MYPN	-378	KCNMB1	782	NFIX	1941
DPYSL3	-1536	SERPIND1	-377	LOC400804	783	SPATS2L	1942
MGC45800	-1535	VSNL1	-376	ASGR1	784	JAG2	1943
LOC650623	-1534	TMIE	-375	ZNF521	785	DMBT1	1944
ATAD2	-1533	SYT13	-374	FLJ42709	786	GABRG1	1945
LMTK2	-1532	AGPAT9	-373	RAET1E	787	HYAL1	1946
C3orf18	-1531	EML4	-372	SCEL	788	HS2ST1	1947
RARRES3	-1530	NOS1AP	-371	EHD2	789	MUSTN1	1948
DCHS2	-1529	GDA	-370	CLEC3B	790	MATN4	1949
HIST2H2BF	-1528	BHMT	-369	MAEL	791	FAM113B	1950
HERC2P4	-1527	SESN3	-368	S1PR3	792	ASPRV1	1951
GJC3	-1526	TLX1NB	-367	BMF	793	SLC2A3	1952
IL25	-1525	CNKSR2	-366	OLAH	794	WASF2	1953
GUSBP3	-1524	FUT9	-365	MORN5	795	MFRP	1954
ZDHHC20	-1523	DEPDC7	-364	C1orf213	796	MYOM2	1955
ZNF699	-1522	C9orf4	-363	CYP3A5	797	TRIP6	1956
TRAT1	-1521	SPIN2A	-362	NMUR1	798	MARCKSL1	1957
GSTO2	-1520	HS6ST2	-361	QRFPR	799	AQP7	1958
ZNF485	-1519	DRGX	-360	ID2	800	HIST1H4C	1959
SIRPG	-1518	HERC5	-359	TTLL6	801	TNFAIP2	1960
AGA	-1517	CRLF1	-358	SLIT2	802	MYO3B	1961
DOCK3	-1516	C21orf88	-357	NTN5	803	SGK2	1962
GBX1	-1515	SYNPR	-356	HIP1R	804	LRGUK	1963
NCKAP5	-1514	CLEC4F	-355	NR5A2	805	ZNRF3	1964
LOC645332	-1513	NTN3	-354	C2orf70	806	BET3L	1965
SPSB4	-1512	CAPN14	-353	PRRG1	807	PLA1A	1966
CATSPER3	-1511	ATRNL1	-352	CRYBB2	808	CD109	1967
KBTBD4	-1510	CACNA2D3	-351	MSC	809	TRPV3	1968
KCNA2	-1509	C9orf140	-350	CREG2	810	RSPH10B2	1969
F7	-1508	LOC338651	-349	NPPA	811	ELF4	1970

STXBP5L	-1507	ACRV1	-348	GAS1	812	SHROOM4	1971
TLR1	-1506	PDE11A	-347	WDR63	813	BMP4	1972
GTF2H2	-1505	ELMO3	-346	EGF	814	TMC5	1973
RIMS2	-1504	OXGR1	-345	LOC348840	815	CTRB1	1974
WDR66	-1503	TSLP	-344	LOC283999	816	TAT	1975
KIF6	-1502	FHOD3	-343	NKX6-2	817	GLTPD1	1976
RGS7BP	-1501	TNR	-342	HDC	818	ENDOG	1977
SIGLEC15	-1500	SH2D5	-341	KLHL31	819	GHR	1978
RELL1	-1499	SCML4	-340	PLA2G4D	820	C19orf33	1979
NCRNA00176	-1498	GPR22	-339	TGFBR3	821	CAPN11	1980
PAFAH1B2	-1497	RAD51AP2	-338	GPR37	822	FILIP1L	1981
PRO0611	-1496	RFPL1	-337	SDK1	823	FSTL3	1982
TNFSF14	-1495	HCG9	-336	VEPH1	824	TRIM16	1983
ZNF669	-1494	FLRT3	-335	TF	825	FLT1	1984
RAC3	-1493	GABRG3	-334	TSKS	826	NAPSA	1985
GHRH	-1492	FGF22	-333	CARD6	827	NXT2	1986
SCN10A	-1491	PRPH	-332	FGG	828	USP13	1987
GTF2H2C	-1490	THSD7A	-331	ST3GAL1	829	EDIL3	1988
LOC283867	-1489	TAL2	-330	LDLRAD2	830	SERPINF1	1989
RACGAP1P	-1488	GRIA3	-329	GGT6	831	CCDC117	1990
RIMBP2	-1487	LOC100192426	-328	C14orf64	832	CCDC89	1991
ERCC8	-1486	ACCN4	-327	CCDC116	833	SLC5A9	1992
C10orf90	-1485	OPRD1	-326	RAPSN	834	LOC100190940	1993
P2RY4	-1484	PLXNC1	-325	CHST6	835	HNF4G	1994
MAN1A1	-1483	NRXN3	-324	SLN	836	PLXDC1	1995
HTR2B	-1482	FAM19A1	-323	CXorf30	837	GJC2	1996
GNG4	-1481	GPR150	-322	SNAI2	838	CCDC28B	1997
FITM2	-1480	PCDH11Y	-321	LOC389033	839	CFD	1998
INPP5F	-1479	CYP26A1	-320	OXT	840	SMOC2	1999
NHLRC4	-1478	GRID2	-319	PRELP	841	CHRDL1	2000
XCR1	-1477	C1QL4	-318	COL2A1	842	CASP5	2001
PPP2R2B	-1476	JAKMIP3	-317	LRRC14B	843	SLC24A4	2002
ZBED3	-1475	OMG	-316	RGS13	844	HS6ST1	2003
NCRNA00087	-1474	KAL1	-315	PZP	845	COL14A1	2004
HOXD13	-1473	ESRRG	-314	LATS2	846	TMEM132B	2005
ZNF648	-1472	KCNIP1	-313	SNHG3	847	C6orf97	2006
SLC7A5P2	-1471	WDR16	-312	NELL2	848	MAP6D1	2007
MIP	-1470	OAS1	-311	PITPNC1	849	SLC22A10	2008
TTC22	-1469	C11orf90	-310	KRT15	850	TNNI3	2009
LOC282997	-1468	ANKRD35	-309	ADAMTS10	851	FAM132A	2010
HIST2H2BA	-1467	PACSIN1	-308	RAMP3	852	SNORA63	2011
CAMP	-1466	NCAN	-307	MATN1	853	SLC44A2	2012
MYBL1	-1465	SCN9A	-306	SMTN	854	VSIG10	2013
CDR1	-1464	C15orf56	-305	LBX2	855	ARHGEF16	2014
PPP2R2C	-1463	VWA5B2	-304	SLC16A9	856	RBP7	2015
GRM7	-1462	COL22A1	-303	MYBPC3	857	CPNE2	2016
SH2B3	-1461	STMN2	-302	IGSF9	858	EYS	2017
CCR10	-1460	LOC100170939	-301	TMEM26	859	POPDC2	2018
ZCWPW2	-1459	ETV7	-300	ADRB3	860	FAM22G	2019
WDR76	-1458	SPOCK3	-299	HTR1F	861	TNNI2	2020
SYN1	-1457	PLEKHA7	-298	LOC90586	862	FLJ39609	2021
KIAA1211	-1456	HHIPL2	-297	CCDC8	863	MAGEB3	2022
CXXC4	-1455	AGBL1	-296	PCSK9	864	C13orf29	2023
PLCXD2	-1454	MAP7D2	-295	DIRC3	865	FAM150A	2024
PCDHA3	-1453	ZCCHC16	-294	FAT3	866	FHAD1	2025
REC8	-1452	CSAG3	-293	TCTEX1D4	867	HDAC1	2026
HSF5	-1451	EGR4	-292	PRKG1	868	ACR	2027
HDX	-1450	IGSF10	-291	BMP2	869	HTR4	2028
SLC9A3	-1449	TPH2	-290	FZD9	870	FAM69A	2029
RND2	-1448	PPFIA2	-289	SRPX2	871	EPS8L3	2030
C1orf220	-1447	DNAH5	-288	LEPREL1	872	ELANE	2031
LOC100240735	-1446	MYADML2	-287	HAPLN2	873	C14orf182	2032
HOTAIR	-1445	OC90	-286	PHLPP1	874	S100A4	2033
CORO1A	-1444	FEV	-285	CAMK2A	875	TMEM200B	2034
C19orf46	-1443	NPY	-284	SNHG3-RCC1	876	C10orf55	2035
HTR7	-1442	OPRL1	-283	DLX4	877	PCDHB7	2036
B4GALT6	-1441	TOX	-282	FBLIM1	878	C7orf61	2037
LY6G5C	-1440	GLRA1	-281	AFF2	879	BGN	2038
NXPH1	-1439	TMIGD2	-280	BTNL9	880	RPSAP52	2039
GABRB3	-1438	FCHO1	-279	WISP2	881	DPH3B	2040
ZDBF2	-1437	FLJ45079	-278	HS3ST5	882	GPR157	2041
EXOC5	-1436	KCNH1	-277	VAV3	883	HOMER3	2042
RNLS	-1435	PIP5KL1	-276	GBP7	884	HIST2H2AC	2043
SLC7A5P1	-1434	KCNK10	-275	PELI2	885	ATP8B1	2044
YIPF6	-1433	C1orf111	-274	SLC2A10	886	CBLN3	2045
TBC1D24	-1432	FAM18A	-273	DLL1	887	TMEM41A	2046
PLEKHB1	-1431	PCDHGC4	-272	FHL3	888	CTTNBP2	2047
PPM1M	-1430	ARAP2	-271	CNGA3	889	FGGY	2048
LRRC6	-1429	PIK3AP1	-270	TMEM217	890	PDGFD	2049
CD96	-1428	TAC3	-269	DMRTA1	891	GIMAP5	2050
LRRN3	-1427	LRRC43	-268	OLFM2	892	RABEP2	2051
C3orf14	-1426	PNCK	-267	PCYT1B	893	PTPRG	2052
DGKZ	-1425	KCNA3	-266	DDO	894	HTR5A	2053
C1orf114	-1424	CDCP1	-265	GSTA1	895	BREA2	2054
SIRPB1	-1423	MFI2	-264	MGAM	896	COL6A2	2055
PCDHGA12	-1422	KCNAB1	-263	ASPA	897	SLC9A3R1	2056
ANKRD20A4	-1421	SLC9A5	-262	GNGT2	898	SFRP1	2057
FFAR3	-1420	C1orf61	-261	LOC221442	899	VGLL4	2058
LRFN2	-1419	FAR2	-260	KBTBD11	900	LRRC59	2059

MDGA1	-1418	RSAD2	-259	FOXF2	901	MUC13	2060
CHGB	-1417	UNC13D	-258	ITGA7	902	ELTD1	2061
CBFB	-1416	ARC	-257	S100A3	903	GYPC	2062
TSPAN5	-1415	GJD2	-256	VASH2	904	LOC100192378	2063
SHOX	-1414	B4GALNT1	-255	OLIG1	905	RASGRF1	2064
CHI3L1	-1413	OMP	-254	MYH6	906	AGBL3	2065
TRIM68	-1412	FBXW12	-253	GRRP1	907	HIGD1B	2066
MAST4	-1411	MRAP2	-252	KCNJ4	908	SNORD97	2067
PLEKHA8	-1410	TNFSF13B	-251	KCNS2	909	SCARF1	2068
SCG3	-1409	TRPC5	-250	GRIK3	910	PLS3	2069
STAT1	-1408	DCX	-249	KCNA5	911	P2RY12	2070
C9orf50	-1407	NAT8L	-248	KRT2	912	RPL23AP32	2071
TNFSF8	-1406	LOC374491	-247	ANO2	913	FRMD7	2072
FAM43A	-1405	KRTAP5-2	-246	FAM131B	914	C9orf153	2073
SMOC1	-1404	ABCD2	-245	SDR42E1	915	KLK10	2074
COL10A1	-1403	PTK6	-244	TSHR	916	ERI3	2075
VCX3A	-1402	POU2F2	-243	KCNE1L	917	DAPK2	2076
HAND1	-1401	SCN2B	-242	KCNIP2	918	SLC25A33	2077
MFSD2A	-1400	SMTNL1	-241	CLEC2L	919	MESP2	2078
CAMKV	-1399	NCAM2	-240	FUT1	920	LHX6	2079
VILL	-1398	C7orf69	-239	CLEC1A	921	SLC5A4	2080
C5orf30	-1397	DGKK	-238	CYP4F11	922	SLFN12	2081
ZNF815	-1396	CNGB1	-237	SLITRK2	923	ASPG	2082
ACOT7	-1395	FGF1	-236	CFI	924	SLC35D1	2083
DCAF12L2	-1394	GDF1	-235	FGF10	925	TNFSF12-TNFSF	2084
NPR2	-1393	TMC3	-234	LEFTY2	926	SERPINF2	2085
MAP3K9	-1392	ATP2B3	-233	SH3KBP1	927	EBF2	2086
EPDR1	-1391	BMPR1B	-232	C1QTNF8	928	S1PR4	2087
FFAR2	-1390	TMEM171	-231	ACSM1	929	ILDR2	2088
EHHADH	-1389	FAM7A2	-230	FLVCR2	930	PIPOX	2089
C22orf36	-1388	DSCAML1	-229	PRKCH	931	CD300A	2090
BRCA2	-1387	RXFP3	-228	C22orf45	932	FAM183A	2091
LOXL1	-1386	RAB26	-227	PTF1A	933	RAB40AL	2092
PRR11	-1385	PCDH11X	-226	ASB9	934	C17orf103	2093
EXOC6B	-1384	C19orf26	-225	TBX18	935	PLXND1	2094
RTBDN	-1383	MYRIP	-224	CCDC64B	936	SLC7A9	2095
MYO5A	-1382	SLC8A2	-223	CALN1	937	FDXR	2096
CD84	-1381	C1QL1	-222	CLEC4G	938	SULT1E1	2097
LRRC56	-1380	APOA2	-221	ENOX1	939	NRIP1	2098
TBX6	-1379	RORB	-220	FBXL7	940	C13orf33	2099
LRRC37A4	-1378	CES7	-219	FAM38A	941	CRABP1	2100
PCDHA6	-1377	LHX9	-218	GIPC3	942	ARHGDIB	2101
SUSD4	-1376	PCDHAC1	-217	CLEC4E	943	LRP5	2102
FLJ42627	-1375	ONECUT2	-216	ATP6V0A4	944	LGI2	2103
NT5C1A	-1374	TAC1	-215	DRD1	945	LMCD1	2104
NRIP3	-1373	PRSS50	-214	GLP2R	946	FAM107B	2105
VSTM2A	-1372	CORO2A	-213	C11orf52	947	FLNC	2106
SCG2	-1371	GLS2	-212	AMZ1	948	LPO	2107
C20orf12	-1370	AHNAK2	-211	HOXD10	949	PDE4B	2108
SLC16A7	-1369	SLC24A2	-210	NLGN3	950	CCDC69	2109
LRIT2	-1368	LHX1	-209	CREB3L1	951	RIN2	2110
ICOS	-1367	CALCB	-208	MDS2	952	HMX1	2111
NMNAT3	-1366	C2orf72	-207	APOL5	953	NCRNA00115	2112
KHDRBS3	-1365	BANK1	-206	GLDC	954	GFI1B	2113
PLAT	-1364	RELN	-205	SCNN1B	955	MXI1	2114
RRH	-1363	KLHL34	-204	RAB40A	956	ECEL1	2115
FER	-1362	CCDC68	-203	KLF14	957	C7orf68	2116
C18orf2	-1361	PALM3	-202	CYP11B1	958	YBX1	2117
LCN1	-1360	C12orf53	-201	BCL11B	959	PDE4A	2118
LOC344595	-1359	FAM92B	-200	NTN4	960	FSTL1	2119
FAM149A	-1358	PAPOLB	-199	ITIH1	961	IFI16	2120
MIPOL1	-1357	MADCAM1	-198	TBX1	962	SEMA6B	2121
MAPT	-1356	SOHLH1	-197	USP2	963	PRSS12	2122
LOXL4	-1355	MGAT4C	-196	DKKL1	964	ARHGEF2	2123
FSCN3	-1354	PLXNB1	-195	TPM2	965	VPREB3	2124
GPR141	-1353	ENPP5	-194	C6orf155	966	SESN2	2125
C16orf11	-1352	CD274	-193	SYDE1	967	FBP1	2126
MFSD4	-1351	DKFZp686O241	-192	GYLTL1B	968	P2RY14	2127
MAP1B	-1350	MPP6	-191	P4HA1	969	TTYH1	2128
SPA17	-1349	LHFPL5	-190	ESR1	970	SYNGR4	2129
CNNM1	-1348	ARHGDIG	-189	ABI3BP	971	GPR182	2130
ACHE	-1347	VGF	-188	BHLHE22	972	FAM26E	2131
LOC151009	-1346	GRIA1	-187	PTH1R	973	MYCT1	2132
TANC2	-1345	RAB39	-186	C10orf10	974	RHBDF2	2133
SFRP4	-1344	C6orf186	-185	CYP2B7P1	975	DUSP5P	2134
SPRY3	-1343	PRSS8	-184	LFNG	976	TAX1BP3	2135
FLJ35390	-1342	SLC22A7	-183	PPP1R14C	977	MYCBP	2136
MCART6	-1341	ZNF157	-182	CCL14	978	NKAIN4	2137
KIAA1244	-1340	PWRN1	-181	TRPM3	979	BST2	2138
DPYSL5	-1339	PRSS3	-180	C7orf54	980	IL6	2139
CA14	-1338	LOC148709	-179	C22orf43	981	CYP4A11	2140
PIGZ	-1337	HRNR	-178	CEACAM22P	982	HAPLN1	2141
GCH1	-1336	ASAM	-177	NKAIN2	983	C1orf115	2142
CYB5R1	-1335	CNTD2	-176	BOC	984	AGXT	2143
CLIP4	-1334	ADCYAP1	-175	FBXW10	985	ZNF860	2144
ALDH5A1	-1333	VSIG2	-174	TSPAN10	986	HMG20B	2145
RFK	-1332	SGK494	-173	RDH16	987	MORN1	2146
NAP1L2	-1331	KCTD16	-172	VWA3A	988	MUSK	2147
NAALADL1	-1330	DHDH	-171	MACROD1	989	ALG6	2148

HIPK3	-1329	DNAJC22	-170	TEAD2	990	LOC100128542	2149
CXCR6	-1328	LOC100126784	-169	CCL16	991	DNAH17	2150
FAM171B	-1327	RET	-168	SEMA5A	992	TGM3	2151
CERCAM	-1326	OSBPL6	-167	CCDC17	993	RNF144B	2152
SCN2A	-1325	CALHM1	-166	PTGDS	994	WWC3	2153
KCTD19	-1324	MARVELD2	-165	TRIP10	995	FSTL4	2154
ST8SIA3	-1323	CNTNAP5	-164	PLD1	996	ZBTB8A	2155
LOC100128292	-1322	TLL2	-163	FAM83A	997	GNB4	2156
HIST1H2AM	-1321	INSRR	-162	ARHGEF10	998	SCGB3A1	2157
DNAJB14	-1320	LRRC50	-161	MYL9	999	POM121L1P	2158
HEY1	-1319	ASTN1	-160	ACAN	1000	MMP23B	2159
RAD54B	-1318	RHEBL1	-159	PKP2	1001	GUCY2D	2160
AKAP4	-1317	HCRTR1	-158	RPA4	1002	TNFRSF10A	2161
JUNB	-1316	PKD1L1	-157	MORC1	1003	DPRXP4	2162
ERC2	-1315	ANKRD43	-156	LOC643008	1004	S1PR1	2163
QRICH2	-1314	C2orf39	-155	FGFR4	1005	WNT5B	2164
HSD17B12	-1313	NCRNA00052	-154	UMODL1	1006	PF4V1	2165
CDH9	-1312	NOS2	-153	PLIN4	1007	C1orf127	2166
CTLA4	-1311	COMP	-152	IL1R2	1008	ISG20	2167
C9orf24	-1310	LASS1	-151	C2CD2	1009	RASGRF2	2168
C6orf1	-1309	C15orf2	-150	FAM9B	1010	SCTR	2169
PPP1R16B	-1308	NLRP14	-149	CERKL	1011	SH3TC2	2170
HBZ	-1307	ZNF536	-148	MATK	1012	SLC6A7	2171
GLB1L3	-1306	C4orf6	-147	TXNDC2	1013	BTBD11	2172
MAP2K6	-1305	MOXD1	-146	POM121L10P	1014	NDN	2173
CDHR1	-1304	ABCG5	-145	SLC9A2	1015	ESPN	2174
TAGLN3	-1303	IL17RD	-144	LOC728819	1016	GIMAP1	2175
TMOD1	-1302	LOC286467	-143	PLLP	1017	PACSIN2	2176
ANK1	-1301	EXPH5	-142	ABP1	1018	EIF4EBP1	2177
C13orf31	-1300	LOC647946	-141	LOC401463	1019	CLEC4C	2178
SLC41A2	-1299	KRT32	-140	WTIP	1020	GADL1	2179
IQCH	-1298	GABRG2	-139	PTCHD2	1021	ADCY1	2180
SYCP3	-1297	MLPH	-138	APOH	1022	GJD4	2181
SPATA18	-1296	APOB	-137	TNMD	1023	MIB2	2182
SHROOM1	-1295	NPHS1	-136	MT2A	1024	SLC12A4	2183
USP27X	-1294	ADAMTS12	-135	PHLDB2	1025	GPX3	2184
HHLA2	-1293	NECAB1	-134	S1PR5	1026	EZR	2185
HAMP	-1292	DRD5	-133	CDH7	1027	WDR78	2186
CCDC108	-1291	C10orf67	-132	ITGA2	1028	LECT1	2187
SGSM1	-1290	NR1I2	-131	LRRC17	1029	RAI14	2188
C9orf139	-1289	PAX5	-130	MLXIPL	1030	ESAM	2189
RACGAP1	-1288	LOC255167	-129	RP1	1031	GPR77	2190
CFLP1	-1287	NRGN	-128	ALPK3	1032	CHP2	2191
RAG1	-1286	GRPR	-127	WIT1	1033	EFEMP2	2192
PRTG	-1285	TPTE2P1	-126	LRP4	1034	HSPB3	2193
C14orf174	-1284	CA10	-125	TBX15	1035	ICK	2194
EGR2	-1283	TLX1	-124	ANGPT2	1036	HS3ST3B1	2195
PSAT1	-1282	SIX3	-123	POLE4	1037	SPDEF	2196
ASAH2	-1281	NR6A1	-122	ST14	1038	SMPDL3B	2197
LOC441455	-1280	GALNT14	-121	IQCA1	1039	C1orf158	2198
TMTC1	-1279	RAG2	-120	NEXN	1040	USP44	2199
CENPE	-1278	KCNB2	-119	MUM1L1	1041	OLFML3	2200
TMEM126B	-1277	PKHD1	-118	COL25A1	1042	MN1	2201
ZC3HAV1L	-1276	TCAP	-117	ALOX12	1043	ZNF593	2202
PHLPP2	-1275	RASGEF1A	-116	LOC145820	1044	CCDC163P	2203
SNPH	-1274	KCNMB2	-115	TPSD1	1045	FAM187B	2204
NEU3	-1273	PMFBP1	-114	RMRP	1046	KITLG	2205
C19orf45	-1272	GPR27	-113	CPB1	1047	PLEKHG1	2206
VAT1L	-1271	CCDC148	-112	KANK1	1048	MS4A8B	2207
TPTE2P3	-1270	PTN	-111	LOC728264	1049	TMEM31	2208
NEIL3	-1269	C8orf34	-110	ADAMTS5	1050	SLC2A9	2209
FAM155A	-1268	SAMD5	-109	SMAGP	1051	GSDMC	2210
PCDHA13	-1267	POU3F2	-108	UGT3A1	1052	CLEC2B	2211
DDX60L	-1266	LRRC55	-107	GPRC5A	1053	TRIM40	2212
CBR3	-1265	KIRREL2	-106	LOC100127888	1054	MAGED4	2213
PCDH9	-1264	MRGPRE	-105	HAS2AS	1055	CUX2	2214
FLJ23867	-1263	EPHA8	-104	PCDH18	1056	FBXO46	2215
LOC641298	-1262	RSPO4	-103	FOXQ1	1057	SERBP1	2216
C4orf48	-1261	C2orf61	-102	SAA1	1058	FAM60A	2217
ENPP4	-1260	FAM181B	-101	CCNJL	1059	SDR16C5	2218
DYNC1I1	-1259	KCNK7	-100	BMP6	1060	LOC642846	2219
MPPED2	-1258	SSTR1	-99	MC2R	1061	OR7D2	2220
APOC4	-1257	C2CD4D	-98	TBXA2R	1062	MS4A15	2221
C2orf40	-1256	ADORA1	-97	SYCE2	1063	TBC1D3B	2222
LOC283267	-1255	PLAC2	-96	SRD5A2	1064	GATA1	2223
PRLR	-1254	CCNO	-95	ZIC4	1065	MLYCD	2224
RGS9BP	-1253	CREB5	-94	LRRC67	1066	CREB3L3	2225
IKZF1	-1252	GREB1L	-93	RCN3	1067	FGR	2226
LOC441208	-1251	CALCA	-92	CLEC11A	1068	BTN2A3	2227
SNORD116-20	-1250	SHANK2	-91	TMEM92	1069	PTCH1	2228
CRLF2	-1249	PP14571	-90	ARPP21	1070	LAMB3	2229
KCNQ2	-1248	SSTR3	-89	FAM198B	1071	TINAGL1	2230
ZFP37	-1247	AKR1C4	-88	CBFA2T3	1072	POU5F1	2231
DOPEY2	-1246	GOLT1A	-87	TRIM29	1073	SPARC	2232
KIAA1984	-1245	C8orf75	-86	MGAT3	1074	VWA1	2233
IPCEF1	-1244	MESTIT1	-85	FABP4	1075	TTC23	2234
KIF5A	-1243	LHCGR	-84	PPP4R1L	1076	C5orf38	2235
RIC3	-1242	BCHE	-83	ITGB1BP3	1077	NHSL1	2236
NKX6-1	-1241	PATE2	-82	CSDAP1	1078	SLC16A3	2237

NAB2	-1240	MAOB	-81	PEG10	1079	ESRRB	2238
CYB561	-1239	RIT2	-80	WLS	1080	GDF5	2239
DBNDD1	-1238	TMC2	-79	C8orf31	1081	IGFBP4	2240
GRIA2	-1237	FAM5B	-78	FOXO1	1082	IGFBP3	2241
FLT3LG	-1236	ISLR2	-77	NEDD9	1083	COL16A1	2242
GDPD1	-1235	HRH3	-76	PGA3	1084	C6orf223	2243
FAM66A	-1234	HAPLN4	-75	SCARNA2	1085	AXL	2244
SPIRE2	-1233	GLT25D2	-74	PKIB	1086	WDR1	2245
TAF1A	-1232	VSX1	-73	XKR4	1087	FAM26D	2246
MLNR	-1231	NEUROG2	-72	OSR1	1088	FOXO4	2247
CAND2	-1230	PPAPDC1A	-71	LOC339524	1089	DNAJB7	2248
AGAP2	-1229	POF1B	-70	GRM8	1090	OR5K1	2249
NKIRAS1	-1228	CES3	-69	OSTalpha	1091	C19orf28	2250
MPV17L	-1227	RBP3	-68	NUPR1	1092	CD36	2251
RGMB	-1226	C3orf55	-67	FRRS1	1093	SEMA3B	2252
DUSP6	-1225	LINGO2	-66	SNED1	1094	SLC16A1	2253
DKK1	-1224	FRMPD4	-65	DPP4	1095	C2orf27A	2254
BCL2L14	-1223	LRRTM4	-64	RBM38	1096	FGFR3	2255
C15orf27	-1222	SCN7A	-63	ESM1	1097	VWA3B	2256
ARL10	-1221	CSMD3	-62	PFKFB1	1098	CDC42EP2	2257
CACNG1	-1220	LOC285401	-61	SPRR1A	1099	CHEK2	2258
GOLGA9P	-1219	RMST	-60	FAM189A2	1100	RPL23P8	2259
EFNA5	-1218	STAT4	-59	PLN	1101	TBC1D3C	2260
PRDM5	-1217	RASEF	-58	DEPDC6	1102	CCDC88B	2261
RUNX1T1	-1216	ISL2	-57	PITPNM2	1103	LDLRAP1	2262
C12orf34	-1215	C21orf62	-56	S100A16	1104	PLIN2	2263
GCET2	-1214	C11orf20	-55	TCF15	1105	TMEM97	2264
RYR1	-1213	CPNE6	-54	ZNF697	1106	PLCG2	2265
C1orf51	-1212	LBXCOR1	-53	NFATC1	1107	PRKAA2	2266
FUT7	-1211	NOX3	-52	FA2H	1108	SKINTL	2267
IL6ST	-1210	PTPRO	-51	CTF1	1109	MST1P9	2268
SIAH3	-1209	AQP6	-50	SLC2A12	1110	SLC22A16	2269
C5	-1208	LGI1	-49	KCNJ2	1111	GIMAP4	2270
PROX1	-1207	ODAM	-48	LOC201651	1112	NAT8	2271
PCDHA2	-1206	SFRP5	-47	EYA4	1113	PRDM1	2272
SNORD116-4	-1205	GDF10	-46	SAMD3	1114	PTTG2	2273
PCDHGB3	-1204	RAB27B	-45	B3GNT7	1115	WBSCR27	2274
C12orf59	-1203	GDNF	-44	KLK14	1116	CASP10	2275
LOC100131193	-1202	PDZK1	-43	LMO4	1117	CDK8	2276
TIGD3	-1201	SHC3	-42	ALDH1A2	1118	FRZB	2277
FLJ10661	-1200	KCNQ3	-41	GABRB2	1119	ABCC9	2278
LOC440356	-1199	NPBWR2	-40	ENAM	1120	TACR1	2279
PRND	-1198	LOC100132111	-39	C13orf15	1121	GPRC5D	2280
CLEC9A	-1197	PPP1R1B	-38	CORIN	1122	RUNX1	2281
DNHD1	-1196	RPH3A	-37	SOBP	1123	SGCD	2282
PARK2	-1195	TMEM45B	-36	RFTN2	1124	KCNK15	2283
RAB39B	-1194	C3orf57	-35	ADARB2	1125	SGSM3	2284
GRK1	-1193	GRIK1	-34	SLC1A6	1126	HS3ST3A1	2285
NCRNA00181	-1192	IFLTD1	-33	C1orf110	1127	MTF2	2286
C1orf226	-1191	IL1RAPL2	-32	ERP27	1128	FAM83F	2287
C14orf33	-1190	C13orf30	-31	KIAA1522	1129	FAM46B	2288
ARNTL2	-1189	STRA6	-30	GSDMD	1130	ACYP1	2289
AP3B2	-1188	CAPN13	-29	PDGFC	1131	FCGBP	2290
SHC1	-1187	ABCG8	-28	PCP2	1132	MEX3A	2291
NKAPL	-1186	CCDC33	-27	GALNTL2	1133	MCTP1	2292
CLDN9	-1185	DOK5	-26	TBL1X	1134	LOC283761	2293
TTBK2	-1184	JAKMIP1	-25	RPPH1	1135	CFHR3	2294
OASL	-1183	KCNK4	-24	MFAP2	1136	PLAC1	2295
CSN2	-1182	SPRED3	-23	HSBP1L1	1137	LPAR6	2296
LHFPL4	-1181	THEM5	-22	GJB2	1138	TNFSF9	2297
ZNF678	-1180	TMEM132D	-21	ST6GAL2	1139	BFSP1	2298
ZNF643	-1179	NPY2R	-20	TMEM125	1140	NCRNA00085	2299
MBNL3	-1178	LOC150568	-19	DSCAM	1141	TM4SF1	2300
LOC728723	-1177	ATP8A2	-18	POU5F1B	1142	FOLR4	2301
GPR19	-1176	LOC283731	-17	LHB	1143	MMP14	2302
KREMEN2	-1175	GNG8	-16	FAM159A	1144	SEMA3C	2303
CALM2	-1174	C4orf50	-15	GNRH2	1145	CDYL2	2304
DNAH14	-1173	NGB	-14	ZAK	1146	CTNNA3	2305
CYP2U1	-1172	USH2A	-13	KCNC2	1147	FGFBP2	2306
ARL6	-1171	MC4R	-12	C1orf203	1148	CPO	2307
C20orf94	-1170	TMEM114	-11	GDF3	1149	HNF4A	2308
CD200R1	-1169	KIAA0125	-10	C20orf54	1150	C2orf58	2309
ZNF486	-1168	PNMT	-9	FAM3D	1151	C10orf72	2310
NEURL	-1167	PROK2	-8	FJX1	1152	BNC2	2311
GOLGA7B	-1166	MGC14436	-7	ATHL1	1153	GFRA1	2312
IRS2	-1165	GLP1R	-6	PRRG2	1154	OSBPL1A	2313
CD83	-1164	POU3F3	-5	HSD17B6	1155	ROR1	2314
TSPYL3	-1163	NPFFR2	-4	ACSM3	1156	KCNJ16	2315
RFC3	-1162	SCRT2	-3	C9orf171	1157	NSMCE1	2316
FAM161A	-1161	OPRM1	-2	CLDN11	1158	NAPRT1	2317
ZIM2	-1160	SALL4	-1	RAB31	1159	AKR1D1	2318
AKAP6	-1159	SH3GL3	1	MOCOS	1160	STARD8	2319
CYP27A1	-1158	SP5	2	GABRA3	1161	TSG1	2320
DDX58	-1157	FGF9	3	SLC43A1	1162	LOC723809	2321
S1PR2	-1156	VIPR2	4	LOC400759	1163	OTX2	2322
HPX	-1155	KIAA1751	5	TMSB15A	1164	C12orf70	2323
AP1S2	-1154	DNAH11	6	ANKRD55	1165	HAPLN3	2324
C16orf89	-1153	C9orf135	7	TCL6	1166	SH2D4B	2325
ABLIM2	-1152	SCGN	8	CD79B	1167	B3GALT6	2326

CEP170L	-1151	CLCNKA	9	NOV	1168	FIGN	2327
SPINT2	-1150	FIBCD1	10	ID3	1169	CRYAB	2328
IFIT1	-1149	DGCR5	11	CDCA7	1170	SIGIRR	2329
SGTB	-1148	CLCNKB	12	CARD10	1171	C3orf59	2330
P2RY10	-1147	CDH23	13	TMEM204	1172	PNOC	2331
RUFY4	-1146	MYO3A	14	FAM71F1	1173	SPON2	2332
HAGHL	-1145	LOC440925	15	CASP9	1174	SNCA	2333
SFTPD	-1144	KCNG3	16	ABRA	1175	INSR	2334
KATNAL2	-1143	ODZ2	17	HPD	1176	CX3CL1	2335
HOMER1	-1142	NRTN	18	RASL11A	1177	LAMA5	2336
LOC730668	-1141	EFCAB1	19	C10orf47	1178	RHOJ	2337
PPM1H	-1140	TCEA3	20	C21orf34	1179	GRAPL	2338
PTAFR	-1139	PPP1R1A	21	NPY5R	1180	CALB1	2339
IGSF5	-1138	HPGD	22	MMP28	1181	ANKRD9	2340
CHRNB4	-1137	GAD1	23	LOC100131726	1182	NR1H4	2341
GUSBL1	-1136	SLC44A4	24	EFNA1	1183	LOC100128288	2342
TMEM180	-1135	SCUBE1	25	POM121L9P	1184	SFTA2	2343
LRRTM1	-1134	NDUFA4L2	26	CSDC2	1185	CDH22	2344
NHLRC1	-1133	LOC154822	27	CLCF1	1186	FOXJ3	2345
SLC22A20	-1132	TMEM163	28	JUB	1187	MRC2	2346
CCR2	-1131	CCBE1	29	NMUR2	1188	RAP2B	2347
CLVS1	-1130	MT3	30	VSTM2B	1189	THBS1	2348
TCEB3B	-1129	WFDC2	31	MFSD7	1190	EIF4EBP3	2349
ATP1A3	-1128	NEUROD1	32	C1orf94	1191	KIF7	2350
SLC10A4	-1127	NECAB2	33	IZUMO1	1192	HEPACAM	2351
SLAMF1	-1126	TMEM52	34	CCDC147	1193	EIF3I	2352
MBOAT2	-1125	PCSK6	35	OLIG2	1194	CD101	2353
CDKL4	-1124	GPR98	36	LPA	1195	NR2F1	2354
FSHR	-1123	HSPB7	37	MGC12916	1196	ARHGAP25	2355
IL19	-1122	TWIST1	38	FGL1	1197	ZNF444	2356
RNF165	-1121	C1QL3	39	MYL4	1198	CSDA	2357
MYO15A	-1120	LANCL3	40	REM1	1199	CYMP	2358
HTR1A	-1119	CABP7	41	SGCG	1200	LRRC4	2359
PTPRJ	-1118	INHA	42	ELSPBP1	1201	CLDN14	2360
UBE2CBP	-1117	ANXA8	43	DCN	1202	AQP10	2361
ARSG	-1116	NXPH4	44	ECSCR	1203	PPBP	2362
CXorf57	-1115	SMTNL2	45	THBS4	1204	CELSR1	2363
ACTC1	-1114	SLC22A3	46	ITGA5	1205	OLFML2A	2364
PTPRU	-1113	ID2B	47	MYB	1206	GSC	2365
GPR123	-1112	ADAMTS17	48	DAAM2	1207	LOC100130581	2366
LOC285696	-1111	SLC44A5	49	LOC100134229	1208	CRIM1	2367
GAS2L2	-1110	RPRM	50	C15orf26	1209	PECAM1	2368
TP73	-1109	PYY2	51	TXLNB	1210	APOL4	2369
LCOR	-1108	POU3F1	52	TNN	1211	XIRP2	2370
C9orf172	-1107	GLI1	53	TEX11	1212	GABRP	2371
IDS	-1106	SCGB1D2	54	RGS6	1213	SLC12A2	2372
ZDHHC11	-1105	LOC100240726	55	STXBP2	1214	TBC1D3H	2373
PROKR1	-1104	EMID2	56	IL5RA	1215	EXOC3L	2374
TMEM191A	-1103	DMKN	57	MPST	1216	XG	2375
RAD18	-1102	TFAP2C	58	KDR	1217	ADRB2	2376
PLCL1	-1101	LRFN5	59	TFAMP1	1218	ZNF275	2377
TCEAL2	-1100	LUZP2	60	FOXC2	1219	CDC42EP1	2378
SMPX	-1099	DACT2	61	CXCR4	1220	TAGLN	2379
DNAJC28	-1098	MB	62	TMEM91	1221	PREX1	2380
ALS2CL	-1097	LRTM2	63	LOC643763	1222	RANBP3L	2381
UBE2T	-1096	MGC16121	64	ELOVL3	1223	PRSS23	2382
PI15	-1095	MT1F	65	SNCAIP	1224	HIST1H3C	2383
GAB2	-1094	MAPK4	66	HSPB2	1225	IFITM3	2384
GPR45	-1093	KIAA1210	67	PADI1	1226	CXorf1	2385
PCDHA4	-1092	TESC	68	TPTE2	1227	SLC25A37	2386
KILLIN	-1091	NTSR1	69	LOC100133893	1228	SNORA10	2387
IL13RA2	-1090	MASP1	70	INHBB	1229	S100A10	2388
SNURF	-1089	UST	71	RBM24	1230	NUP210L	2389
BEST4	-1088	LRRC3	72	LPAR3	1231	GIMAP8	2390
TDRKH	-1087	SLC6A9	73	ARHGAP29	1232	C22orf13	2391
LRRIQ1	-1086	EMID1	74	C5orf23	1233	RILPL2	2392
FIGF	-1085	LOC284276	75	RFPL3S	1234	KCNN2	2393
PKD1	-1084	FITM1	76	CCDC3	1235	CACNG6	2394
IL17RE	-1083	HYMAI	77	TSSK3	1236	PRX	2395
EIF2AK2	-1082	COL6A6	78	ANXA11	1237	ZNF572	2396
HHLA1	-1081	KLK4	79	ARHGAP42	1238	CLDN23	2397
APOOL	-1080	SLC16A11	80	LOC157627	1239	RASL10A	2398
C1orf230	-1079	KRT17	81	LRRC70	1240	ROBO4	2399
TTC39B	-1078	TDO2	82	SMOX	1241	C20orf202	2400
IMPG2	-1077	CYP4B1	83	HSD3B1	1242	BLMH	2401
BCAN	-1076	KRT80	84	SYN3	1243	FMOD	2402
CRYBA4	-1075	SSTR5	85	H6PD	1244	DLX6AS	2403
CDKL2	-1074	MGC12982	86	HLX	1245	RPL11	2404
SLC45A2	-1073	FAM131C	87	SDC3	1246	KCNJ6	2405
SCXB	-1072	IL17B	88	PPP1R3C	1247	PCP4	2406
ZFPM1	-1071	KCNK2	89	CDH20	1248	GPAA1	2407
LOC100130386	-1070	CYP4F12	90	TGFB2	1249	HVCN1	2408
C12orf75	-1069	FGF12	91	GPIHBP1	1250	CCNL2	2409
KIAA1614	-1068	C11orf86	92	CYP1B1	1251	PSTPIP2	2410
FAM151B	-1067	HPSE2	93	SLC35C2	1252	HES4	2411
ENDOD1	-1066	CGNL1	94	GLIS3	1253	HUS1B	2412
RAB11FIP4	-1065	COL13A1	95	CYP3A4	1254	KCNV1	2413
DPF1	-1064	STXBP6	96	KCNA4	1255	TSPAN19	2414
IFIH1	-1063	PRMT8	97	SPTLC3	1256	DIO3OS	2415

FAM134B	-1062	HK2	98	CSRP2	1257	SFRP2	2416
ANKRD36B	-1061	EMX2OS	99	CA4	1258	NADK	2417
SLC32A1	-1060	ST8SIA1	100	CTRB2	1259	CCL15	2418
ZSCAN23	-1059	LOC146336	101	NRIP2	1260	DENND2D	2419
CKS2	-1058	HIF3A	102	CYP4Z2P	1261	POLR3H	2420
CDH4	-1057	SLC13A3	103	GPR160	1262	SNORA38	2421
CCDC144B	-1056	AGXT2L1	104	MT1X	1263	SERPINH1	2422
TUBB2A	-1055	THPO	105	ITGA10	1264	CYP2S1	2423
SUSD1	-1054	WIF1	106	SLC38A3	1265	LAMA3	2424
IL18BP	-1053	SLPI	107	KLHL30	1266	LOC389332	2425
TLE6	-1052	OCA2	108	ZNF703	1267	FLJ42875	2426
PCDHGA11	-1051	NPAS2	109	KRT28	1268	GSG1	2427
LDHD	-1050	SYT6	110	LY6H	1269	CHI3L2	2428
RGS22	-1049	IGF2BP2	111	KIAA1644	1270	LYPLA2	2429
BARHL2	-1048	LOC284749	112	TMEM61	1271	ATP2C2	2430
HELLS	-1047	C10orf105	113	PLA2G4F	1272	ZNF560	2431
CCDC87	-1046	KRT71	114	TSPAN1	1273	PLAU	2432
DSCR9	-1045	BIK	115	RASIP1	1274	C14orf178	2433
CACNA1A	-1044	APCDD1	116	PTP4A3	1275	NR2F2	2434
CYP1A1	-1043	FZD10	117	SERPINA5	1276	VPS37D	2435
TCEAL5	-1042	FAM19A5	118	PPIC	1277	HSPBAP1	2436
ZNF81	-1041	RYR2	119	EDNRB	1278	OSBPL5	2437
SEZ6L	-1040	EMX2	120	ADAMTS8	1279	TXNDC11	2438
XPR1	-1039	C19orf51	121	PAX7	1280	C3orf42	2439
RBMXL2	-1038	NKD1	122	RERGL	1281	FZD4	2440
PRKCB	-1037	CLEC4GP1	123	GSTA2	1282	CITED2	2441
LOC151174	-1036	C20orf200	124	CDH13	1283	C5orf47	2442
ANKRD44	-1035	PLAGL1	125	IRGM	1284	OR13J1	2443
IPMK	-1034	ADRA1B	126	CGB7	1285	SUMO1P1	2444
RGL3	-1033	ELFN1	127	SOX8	1286	MAP3K8	2445
LOC730811	-1032	GPR139	128	C21orf121	1287	C12orf54	2446
CCDC144A	-1031	COL17A1	129	LOC388242	1288	IL11	2447
NAAA	-1030	WNT4	130	C1QTNF6	1289	MIF	2448
NBPF16	-1029	RSPO3	131	CAV2	1290	OR3A2	2449
B3GNT8	-1028	NR0B2	132	C8orf73	1291	CCDC134	2450
ProSAPiP1	-1027	SYPL2	133	LOC149134	1292	MXRA8	2451
LOC729799	-1026	KCNQ1	134	ASMT	1293	PSPN	2452
FLJ45983	-1025	GREM2	135	ATP2A3	1294	IGFBP5	2453
RGS5	-1024	GCOM1	136	ADCY4	1295	NGF	2454
NEBL	-1023	FCN3	137	TPH1	1296	DSC2	2455
LIN28B	-1022	CHMP4C	138	MRVI1	1297	FLJ40292	2456
C1orf92	-1021	ST8SIA5	139	SH3BP5	1298	CEACAM4	2457
LOC401431	-1020	SMPDL3A	140	LGALS12	1299	DNAJC12	2458
TIAM1	-1019	MYLK3	141	TMEM233	1300	PAX2	2459
LOC387646	-1018	HECW1	142	TMTC4	1301	TMEM50A	2460
LOC100129726	-1017	TSKU	143	CHN2	1302	HGD	2461
FAM13C	-1016	C1orf228	144	BDKRB2	1303	AKR7L	2462
SVOP	-1015	DLX3	145	GRAMD4	1304	INSL5	2463
SPOCD1	-1014	FOXD2	146	HAO2	1305	TNC	2464
BSN	-1013	SLC5A8	147	NPY6R	1306	PCDH12	2465
LOC653653	-1012	PDE2A	148	IGLL3	1307	C8orf56	2466
GAS2L3	-1011	GATA5	149	C20orf46	1308	FAM23A	2467
LOC100240734	-1010	FOXD1	150	SLC7A11	1309	HES1	2468
AKR1C1	-1009	HRASLS	151	CRYBB3	1310	C1R	2469
PNPLA5	-1008	FAM153B	152	EMP2	1311	MALL	2470
KLF7	-1007	KCNS1	153	C7orf58	1312	GLIS1	2471
GOLGA6L6	-1006	SH3RF3	154	MYH11	1313	CTHRC1	2472
SPOCK2	-1005	GPR143	155	GINS4	1314	LIPG	2473
SPAG1	-1004	CELA2A	156	ALOXE3	1315	FZD2	2474
PFN4	-1003	C6orf141	157	C8orf58	1316	MICAL2	2475
KIAA1383	-1002	TMEM132C	158	PSD3	1317	LOC100216001	2476
ZNF454	-1001	MCOLN3	159	FAM83H	1318	NMU	2477
CLYBL	-1000	EGFR	160	GAD2	1319	ZDHHC9	2478
NR1I3	-999	KLHL4	161	CAMK1G	1320	SMARCD3	2479
IRX5	-998	ZIC3	162	GPX8	1321	TMCO4	2480
TCHH	-997	SLC38A4	163	CDC42EP5	1322	C3orf47	2481
ZNF382	-996	KLK15	164	AADAC	1323	C10orf116	2482
USP49	-995	SYCE1L	165	C5orf13	1324	PPARGC1A	2483
TRIM21	-994	KAZ	166	KIAA1217	1325	PLEKHO1	2484
SALL2	-993	TTLL2	167	CAT	1326	RRP7B	2485
NLRP4	-992	CHRNA2	168	SLC7A10	1327	SNORA68	2486
NUDT8	-991	APOBEC2	169	SPINK13	1328	OMD	2487
PAG1	-990	WNT9A	170	ADAP1	1329	AP2A1	2488
KLHL8	-989	ZNF385B	171	LCNL1	1330	S100A8	2489
RTL1	-988	FAM153A	172	CGB2	1331	APCDD1L	2490
PRSS22	-987	CIB4	173	SOCS3	1332	GJA4	2491
BEAN	-986	MAP3K15	174	CNN1	1333	SNORA71D	2492
KCNC1	-985	FAM110C	175	PTGDR	1334	FAM181A	2493
ZNF792	-984	MGC2889	176	GKN1	1335	MPL	2494
PCDHA9	-983	AQP2	177	GPR32	1336	CLIC6	2495
MAN1C1	-982	TSPAN12	178	TNXB	1337	KCNQ4	2496
CPT1C	-981	C1orf126	179	CSMD1	1338	MGP	2497
C6	-980	KCNK13	180	SLC22A23	1339	COL21A1	2498
FAM190A	-979	AFP	181	SHMT2	1340	MTUS1	2499
UNC13C	-978	KRT16	182	GALNT9	1341	RHOU	2500
PRDM11	-977	GNA14	183	MATN2	1342	HSD17B13	2501
CYP2J2	-976	HHIP	184	ITPRIP	1343	TMPRSS3	2502
KIAA0664P3	-975	FAM153C	185	TSPO	1344	CCL27	2503
KIRREL3	-974	VTCN1	186	SLURP1	1345	SNORD22	2504

MMP16	-973	MAGEL2	187	TMEM139	1346	CCL28	2505
C17orf107	-972	EFNA4	188	ABCA4	1347	C5orf33	2506
RTN1	-971	RAB37	189	LOC100271722	1348	CTSK	2507
PM20D1	-970	PCOLCE2	190	ODF3B	1349	LOC400891	2508
TCEAL7	-969	PAWR	191	C4orf47	1350	CD72	2509
CDO1	-968	DIO3	192	BEST3	1351	RGS8	2510
ANKRD34B	-967	ZNF804A	193	RNF148	1352	FAM71A	2511
SLC30A4	-966	PRRX2	194	WNT7B	1353	C10orf50	2512
CCNI2	-965	HSD11B2	195	ITPKB	1354	CYBRD1	2513
PLCB4	-964	CYP39A1	196	GPR149	1355	PYDC1	2514
PAK3	-963	TMEM184A	197	CACNA1E	1356	CD97	2515
ENTPD3	-962	ABO	198	SLCO6A1	1357	PTH2R	2516
CCR5	-961	KIF17	199	MMD2	1358	JAG1	2517
APOA5	-960	NOX4	200	CPA1	1359	EPS8	2518
C10orf128	-959	FXYD7	201	LMX1A	1360	NUDT18	2519
EME2	-958	C9orf66	202	SHH	1361	SNX33	2520
PRLH	-957	C6orf142	203	SGK223	1362	CEBPB	2521
STARD4	-956	HFM1	204	MUC1	1363	CHST13	2522
ROR2	-955	DARC	205	FLJ13224	1364	MCHR2	2523
MX2	-954	HOXA10	206	C3orf72	1365	UQCRHL	2524
FAAH2	-953	ANXA8L2	207	TBC1D3G	1366	DNAJB4	2525
ELMOD1	-952	FMO1	208	ID4	1367	P2RX1	2526
ASFMR1	-951	HPN	209	PRODH	1368	PASD1	2527
ZNF214	-950	C11orf88	210	C1QTNF7	1369	EPHA2	2528
C17orf57	-949	NCRNA00092	211	CTSG	1370	MYO5C	2529
BAAT	-948	DDIT4L	212	MYO10	1371	CTSC	2530
LOC645166	-947	PLA2G1B	213	COLEC12	1372	MFNG	2531
CTAG1B	-946	CLDN1	214	LOC100144604	1373	RRAS	2532
RS1	-945	PHACTR3	215	TEX14	1374	HTR1E	2533
ALCAM	-944	C4orf31	216	GPC3	1375	HIST1H4J	2534
AKR1E2	-943	TPSG1	217	HSPC072	1376	RBMS2	2535
IGSF21	-942	KCTD8	218	CABP4	1377	SLC39A14	2536
TPP1	-941	CITED4	219	SLCO1B3	1378	IGF2AS	2537
GOLGA2B	-940	ZP1	220	BAIAP2L2	1379	C12orf39	2538
NAV1	-939	HOXA9	221	SLC26A7	1380	GPM6B	2539
SCN3A	-938	PROK1	222	C6orf124	1381	SOX30	2540
ZNF30	-937	C10orf11	223	GALNT10	1382	C10orf79	2541
MYO1B	-936	FBXL21	224	LOC440461	1383	CPT2	2542
HIST1H2BG	-935	CNFN	225	PLVAP	1384	CACNA2D4	2543
IFIT1B	-934	GATA4	226	C9orf47	1385	DHRS3	2544
EGFLAM	-933	KLK3	227	EPHB4	1386	CHST2	2545
CARD9	-932	TRIM74	228	OR7E37P	1387	DIO2	2546
ADD3	-931	GABRR1	229	LOC729603	1388	CRYGS	2547
DCT	-930	GPRIN2	230	SRPX	1389	TRNAU1AP	2548
TH	-929	ADAMTS2	231	RAMP2	1390	HGF	2549
RINL	-928	FLJ43390	232	C9orf167	1391	TOP1MT	2550
F2RL2	-927	PCDH8	233	KCNA1	1392	ADAMTS7	2551
MAPK13	-926	CNGA1	234	LRRTM3	1393	PRINS	2552
BATF2	-925	TPD52L1	235	AOC3	1394	EMX1	2553
GATA3	-924	PPP1R3G	236	OSBPL10	1395	RASL10B	2554
BIRC3	-923	SLC26A4	237	TDGF3	1396	SEMA3E	2555
ZMYND10	-922	SPINK5	238	RGS9	1397	C16orf82	2556
TPPP	-921	C11orf92	239	LSR	1398	RAB20	2557
INTU	-920	BCL11A	240	GGT5	1399	HSPC157	2558
C16orf45	-919	ADRB1	241	ASB4	1400	RPL35A	2559
ANKRD20A3	-918	CRTAC1	242	FLJ37453	1401	PAQR8	2560
TTBK1	-917	OPCML	243	PXDN	1402	CTNNA1	2561
PARD3B	-916	PLEK2	244	BACE2	1403	NUDT1	2562
BCAS1	-915	MYH14	245	RASGRP2	1404	KSR1	2563
PALM2	-914	SLITRK1	246	CPN2	1405	CASQ2	2564
C8ORFK29	-913	TFCP2L1	247	DPP10	1406	KRT23	2565
IGSF9B	-912	SOX2	248	C4A	1407	ADAMTS16	2566
STAC	-911	GIPC2	249	CXorf59	1408	MDFIC	2567
DCLK1	-910	OXER1	250	PCP4L1	1409	TREML3	2568
BMP7	-909	DUOXA2	251	CYP8B1	1410	ACADVL	2569
DYX1C1	-908	SLITRK5	252	GBP2	1411	FXYD6	2570
ISL1	-907	BAIAP2L1	253	AQP8	1412	TMEM229A	2571
ATP9A	-906	CYP2W1	254	LOC401127	1413	ECM2	2572
ANK2	-905	SYDE2	255	NFE2	1414	RABGGTB	2573
MAGEE2	-904	CACNA1I	256	psiTPTE22	1415	E2F2	2574
RGS17	-903	SLCO5A1	257	C22orf31	1416	C1orf130	2575
STOX2	-902	LOC158696	258	B3GALTL	1417	E2F7	2576
PLEKHA6	-901	LRRC1	259	NXF2	1418	LBP	2577
EPHX3	-900	TMEFF2	260	CC2D2B	1419	SEL1L2	2578
LOC283392	-899	ATAD3C	261	CCDC102B	1420	MFAP5	2579
C10orf93	-898	DAND5	262	CETP	1421	FAM74A3	2580
SNORD116-28	-897	BTNL8	263	ENPP3	1422	ANKRD2	2581
TMEM232	-896	ZSCAN1	264	DAPL1	1423	GRIN2D	2582
TLX2	-895	COX4I2	265	CLDN7	1424	TBC1D10A	2583
SFMBT2	-894	LOC284233	266	RDH5	1425	PES1	2584
CDKN3	-893	EGFL6	267	ANPEP	1426	C20orf141	2585
LCN10	-892	CTRC	268	PDGFRB	1427	RPL22	2586
ASTN2	-891	NPR1	269	LPHN2	1428	PER1	2587
BRIP1	-890	ALOX12B	270	CEBPD	1429	SULT2B1	2588
CAPN5	-889	SELENBP1	271	HHLA3	1430	CNN2	2589
ZNF488	-888	CYP21A2	272	SLC7A2	1431	KIAA1462	2590
FAM40B	-887	PAGE4	273	PDE6H	1432	ZBTB17	2591
UHMK1	-886	PTPRQ	274	LOC100124692	1433	MITF	2592
SOX9	-885	KLK1	275	NR5A1	1434	POR	2593

BASP1	-884	MT1E	276	DOK7	1435	PPIF	2594
PRSS27	-883	OSR2	277	G6PC2	1436	GABRB1	2595
PRCD	-882	LMAN1L	278	GHRHR	1437	EXOC3L2	2596
LOC441046	-881	LY6D	279	SLC18A3	1438	MKNK1	2597
FAM161B	-880	PLEKHG6	280	GIPR	1439	ATOH7	2598
LHFPL1	-879	RAET1G	281	NTN1	1440	NXNL2	2599
CD207	-878	MEOX2	282	SLC35F1	1441	PPY2	2600
OR2C1	-877	BDKRB1	283	NAT2	1442	RAB43	2601
C12orf29	-876	TRPM6	284	ENO1	1443	SLC6A4	2602
MYO18B	-875	ECHDC3	285	ARHGEF15	1444	MIR155HG	2603
HIST1H4I	-874	AKAP3	286	LOC257358	1445	MEF2C	2604
RNF112	-873	SLC4A10	287	DNAH9	1446	CLDN15	2605
ZNF608	-872	PHYHD1	288	METTL7B	1447	CNR1	2606
TSPYL6	-871	ANGPTL1	289	LGR6	1448	FES	2607
HOXB5	-870	RFX8	290	ABCG2	1449	LPAR4	2608
GARNL3	-869	TMEM196	291	PDK4	1450	CDC20	2609
GFOD1	-868	NTRK2	292	FBXL13	1451	OR5K2	2610
FBXL2	-867	SLC30A10	293	CCL25	1452	COL5A3	2611
SLC25A43	-866	C1orf172	294	IFITM2	1453	RNASE1	2612
PLCXD3	-865	PTER	295	RAB3B	1454	ZPLD1	2613
TOX3	-864	SLC13A5	296	VIT	1455	GRASP	2614
SRGAP1	-863	CASP12	297	CHODL	1456	IRX2	2615
KIAA0319	-862	TNNT2	298	C20orf160	1457	GALE	2616
FBXO17	-861	COL27A1	299	MYLK	1458	ROPN1L	2617
NEFL	-860	DSP	300	TCERG1L	1459	SH2D3A	2618
FCGR2B	-859	GCK	301	MAPK15	1460	C9orf84	2619
GPR61	-858	MSX2	302	C17orf82	1461	ATAD3B	2620
KCNB1	-857	RYR3	303	PLA2G5	1462	PAPPA2	2621
CLDN10	-856	GPR133	304	CCL21	1463	NPHS2	2622
GGN	-855	FATE1	305	SPNS3	1464	SH3GLB1	2623
KCNG4	-854	GPR56	306	LRRC15	1465	KRT12	2624
C17orf54	-853	FLJ11235	307	C20orf203	1466	CAPZB	2625
NLN	-852	GPER	308	KCNE4	1467	TRIAP1	2626
RELL2	-851	TFPI2	309	LARGE	1468	RNF39	2627
CD44	-850	TLL1	310	CARD16	1469	SARDH	2628
FAM169A	-849	SOX3	311	FAM176B	1470	CXorf22	2629
NUDT17	-848	DSG2	312	REN	1471	TBX4	2630
LRFN1	-847	FOXP2	313	CCKBR	1472	RGS4	2631
RHOF	-846	CMTM8	314	TMEM63A	1473	HOXA5	2632
IKZF3	-845	GABRD	315	C15orf54	1474	FGF21	2633
GAL3ST2	-844	NSUN7	316	PHKG1	1475	PPP3CC	2634
FAM63B	-843	OIT3	317	ATP11A	1476	MGC72080	2635
SRD5A1	-842	FAM196A	318	OTOF	1477	C7orf10	2636
FAM106C	-841	ZMAT4	319	SPNS2	1478	EVPLL	2637
TCP11L1	-840	CLRN1	320	SCARB1	1479	TNFSF11	2638
PACSIN3	-839	TMEM30B	321	MPP7	1480	TMPRSS2	2639
PSG9	-838	RHBG	322	TES	1481	KLHDC8B	2640
SIDT1	-837	GPR144	323	WNT2B	1482	PARS2	2641
CMPK2	-836	TST	324	AK3L1	1483	C22orf32	2642
SYT14	-835	SPINK4	325	FOXS1	1484	CCT6B	2643
FAM66C	-834	CACHD1	326	TESK2	1485	MYOF	2644
TDRD7	-833	SLCO4A1	327	ADAT3	1486	AVPR2	2645
EGR1	-832	KCNH7	328	MYOM3	1487	FCER2	2646
ANO5	-831	PPAP2C	329	GPR6	1488	RPLP0	2647
RGPD8	-830	ACPT	330	ACSS1	1489	LAMB1	2648
HIST1H2BL	-829	SNTB1	331	RTP2	1490	CRYGN	2649
GUCA1A	-828	SLITRK4	332	CYP4Z1	1491	CCRL2	2650
NCRNA00202	-827	LOC286002	333	GALM	1492	GPATCH3	2651
ZNF883	-826	RAB38	334	MMP1	1493	RPL12	2652
ALK	-825	PDGFRL	335	CXorf36	1494	CMPK1	2653
ASB14	-824	FBN2	336	RASGRP3	1495	MKX	2654
PRICKLE1	-823	FLJ42289	337	ANGPTL4	1496	MEIS3P1	2655
PLCH2	-822	BCL2L12	338	ADAM33	1497	LIF	2656
ZDHHC21	-821	KRT5	339	HSD11B1	1498	ORAI1	2657
PODNL1	-820	MEIS3	340	OR7C1	1499	TEKT4	2658
CSRNP3	-819	ASS1	341	BCAT1	1500	DPCR1	2659
LOC344967	-818	GABRA4	342	PGM5	1501	FAM19A2	2660
LOC145837	-817	NOTUM	343	CCDC141	1502	SIGLEC12	2661
PAQR6	-816	SLC12A1	344	EIF3IP1	1503	CPS1	2662
PON1	-815	CCRL1	345	PNPLA3	1504	LRP2BP	2663
MYH15	-814	TEKT1	346	TMC4	1505	MAFK	2664
POLR3G	-813	PRSS35	347	C1orf95	1506	LAMB4	2665
SLC1A1	-812	LPHN3	348	CPLX3	1507	PDLIM2	2666
CASC1	-811	CACNA1H	349	RNF122	1508	KSR2	2667
PWRN2	-810	GNG11	350	ARID5A	1509	C4orf26	2668
TRHDE	-809	C18orf34	351	DOK6	1510	VGLL2	2669
KCNH5	-808	CNTNAP3	352	TM4SF18	1511	NADSYN1	2670
LDHAL6A	-807	FAM126A	353	CIDEC	1512	ZNF702P	2671
SYT14L	-806	MT1G	354	EGFL7	1513	FAM107A	2672
PRKG2	-805	PTPRD	355	LRRC39	1514	SGK1	2673
CMTM5	-804	MYOM1	356	IL15RA	1515	PRKCDBP	2674
RSPO2	-803	TRIM55	357	SLFNL1	1516	CRHR2	2675
CARTPT	-802	ATP4A	358	ACMSD	1517	WSB1	2676
LOXHD1	-801	TDGF1	359	LYG1	1518	EPS8L1	2677
BRSK2	-800	PROM1	360	ZIC1	1519	HCG22	2678
ZNF727	-799	CLDN2	361	ASXL3	1520	HABP2	2679
ZSWIM2	-798	AIRE	362	TNFAIP6	1521	IRF3	2680
GPR39	-797	KRT8	363	VAMP5	1522	NDRG2	2681
TFAP2B	-796	CILP	364	ADAMTS14	1523	ABCA9	2682

XKR5	-795	NGEF	365	P2RY8	1524	COX6A2	2683
PIRT	-794	GRP	366	ID1	1525	RPL13AP6	2684
CDC14C	-793	AMHR2	367	KCNE3	1526	PRKAG3	2685
RASSF6	-792	FAM166B	368	CD22	1527	SLC16A2	2686
FAM133A	-791	SLC6A16	369	ARHGAP6	1528	YPEL2	2687
FLJ37307	-790	CADM3	370	C21orf130	1529	LOC441869	2688
FEZ1	-789	FAT1	371	CYP17A1	1530	TBL1Y	2689
GPR155	-788	SYTL4	372	L1TD1	1531	C1orf144	2690
HES3	-787	PAQR5	373	GABRQ	1532	KLF4	2691
CACNB4	-786	C21orf29	374	HTRA3	1533	ARFGAP3	2692
WDR38	-785	GPR63	375	PTPRE	1534	C20orf123	2693
DUSP2	-784	GCKR	376	LOC644165	1535	C1QL2	2694
SRGAP3	-783	SCARNA7	377	ITGB1BP2	1536	FTCD	2695
LOC145783	-782	PKNOX2	378	SNHG5	1537	MST1P2	2696
SHOX2	-781	FOXL2	379	ST8SIA4	1538	KCNK1	2697
FSD1L	-780	ABCC6P1	380	DUOX2	1539	SNORA5A	2698
ANKRD53	-779	CTSL2	381	WT1	1540	C2orf82	2699
CGREF1	-778	THRB	382	GIMAP7	1541	KLK6	2700
PABPC1L2B	-777	OBSCN	383	LIMS2	1542	TP53I11	2701
NTNG1	-776	CPM	384	IL4R	1543	UTS2D	2702
RFPL1S	-775	TMEM37	385	GUCY1A2	1544	OR4F29	2703
LRP8	-774	KRT9	386	TREM1	1545	DCAF12L1	2704
NCRNA00167	-773	KIAA1024	387	ITGB5	1546	PALM2-AKAP2	2705
LOC100128977	-772	HOPX	388	LRRC7	1547	SECTM1	2706
FOXE1	-771	GPRC5C	389	PLA2G4A	1548	RTP1	2707
CYGB	-770	PRSS30P	390	CDC6	1549	WDTC1	2708
CYP26C1	-769	LOC121838	391	CLDN4	1550	KCNK3	2709
HIST1H2AG	-768	ADRA2C	392	HEPH	1551	DVWA	2710
C6orf208	-767	FGFRL1	393	TGIF2	1552	GCNT4	2711
GXYLT2	-766	TSPAN6	394	WWTR1	1553	C9orf43	2712
RBM43	-765	TPSB2	395	NIPAL1	1554	C22orf40	2713
RPS6KA6	-764	GRB14	396	LOC646999	1555	GPR146	2714
VN1R1	-763	SP6	397	NFATC4	1556	SLC13A2	2715
AQP4	-762	TWIST2	398	GSG1L	1557	SOX2OT	2716
SPTB	-761	COL6A4P2	399	RPS6KA1	1558	HPCA	2717
CNKSR3	-760	MDFI	400	TAL1	1559	PKD2L1	2718
PBX4	-759	UPK2	401	ODF3L1	1560	TAAR1	2719
TMEM20	-758	IL1RL1	402	CXADRP3	1561	UBE2J1	2720
ULBP1	-757	CALB2	403	RBM47	1562	ABCC6	2721
KIAA1409	-756	FOXE3	404	SCARNA10	1563	ASB2	2722
PLXNB3	-755	ZNF503	405	ADAMTSL3	1564	VASP	2723
NLGN1	-754	ACTG2	406	RNF152	1565	SLC17A4	2724
NOTO	-753	NTNG2	407	ANTXR1	1566	OPN1SW	2725
PELI1	-752	NPNT	408	KALRN	1567	FIBIN	2726
CADM1	-751	HSD3B2	409	ERG	1568	ITIH5	2727
FAM168A	-750	SLCO1C1	410	GPR21	1569	MLC1	2728
PKLR	-749	FADS6	411	COL9A3	1570	TUBB6	2729
NEK10	-748	SMO	412	DOCK1	1571	UROC1	2730
LOC100133991	-747	SLC27A6	413	ZNF280A	1572	EIF3D	2731
PITX3	-746	AVPR1A	414	CLIC3	1573	ZFP57	2732
VAX2	-745	A4GALT	415	FGF3	1574	PON2	2733
SYCP2	-744	CD300LG	416	SNORA7B	1575	RNF5	2734
LASS6	-743	AGMAT	417	YAP1	1576	ZNF436	2735
PDE1C	-742	XKRX	418	FLJ26850	1577	SYTL2	2736
KLHL13	-741	TMEM98	419	OPLAH	1578	INPP5B	2737
RAB3C	-740	C11orf93	420	PRR15	1579	CMA1	2738
ANKRD34A	-739	LOC388387	421	HHEX	1580	GYS1	2739
NKX2-8	-738	CHST4	422	ITPRIPL2	1581	POLR2H	2740
PPEF1	-737	SEMA3A	423	SCUBE2	1582	BCL9L	2741
TIMP4	-736	ARHGEF19	424	RPL13AP3	1583	FBXO24	2742
OLR1	-735	CDH12	425	B4GALT2	1584	TGM5	2743
NTRK1	-734	LOC100129055	426	TMPRSS13	1585	IFT20	2744
TMEM130	-733	B4GALNT3	427	SYTL5	1586	IMPDH1	2745
PHYHIPL	-732	TFF3	428	NPY1R	1587	REST	2746
B3GAT1	-731	COL23A1	429	AGPAT2	1588	MRPL37	2747
DRD2	-730	FAM138F	430	GPM6A	1589	SCARA5	2748
HMP19	-729	SLC16A10	431	CLDN16	1590	RHPN1	2749
HIST1H3H	-728	DSCR6	432	FKBP10	1591	CNN3	2750
A2BP1	-727	C22orf42	433	MXRA5	1592	LBH	2751
KIAA1045	-726	ATP10A	434	RDH12	1593	LMF1	2752
ZNF683	-725	LLGL2	435	UPK1A	1594	GRK5	2753
S100B	-724	CA2	436	PBXIP1	1595	SKAP1	2754
IGFL4	-723	TMEM45A	437	ACRC	1596	FST	2755
MYCNOS	-722	EIF4E1B	438	MRS2P2	1597	KRT84	2756
DPYS	-721	AZGP1	439	ASB5	1598	SNORA57	2757
NHS	-720	FOXL1	440	ABI3	1599	LAMB2	2758
KCNJ5	-719	C10orf82	441	LRRC32	1600	SRCRB4D	2759
LOC84856	-718	NAGS	442	NUAK1	1601	CALCR	2760
GALNT13	-717	GDF6	443	BID	1602	ATP6AP1L	2761
HMGCLL1	-716	CCDC48	444	INPP5A	1603	LAPTM4B	2762
SPRY4	-715	SCNN1A	445	TNFAIP8L1	1604	DET1	2763
DDX60	-714	MT1M	446	STON1	1605	SELE	2764
DNM1	-713	VDR	447	MATN3	1606	C14orf53	2765
TMEM38A	-712	CPA3	448	BARX2	1607	UGT1A6	2766
SLC5A2	-711	KIF19	449	FAM101B	1608	C9orf96	2767
CDH18	-710	VWA5B1	450	RASSF2	1609	RYK	2768
SLC39A2	-709	HTR6	451	MFGE8	1610	HMSD	2769
PRKCE	-708	BMP1	452	F2RL1	1611	PNPLA1	2770
CMAH	-707	C17orf44	453	GCNT2	1612	C19orf22	2771

DDX25	-706	SLC22A11	454	PKP1	1613	GPR128	2772
ANGPT1	-705	C1orf170	455	SOD3	1614	JUP	2773
GIP	-704	LOC389493	456	AKR7A3	1615	LTC4S	2774
ABHD1	-703	TNFAIP8L3	457	GPR50	1616	SPTBN5	2775
DLG2	-702	LEF1	458	SORCS2	1617	FILIP1	2776
CCL3L3	-701	FBXO32	459	EPHA4	1618	CLIC5	2777
GCNT1	-700	SMAD6	460	KRT1	1619	LIMS3	2778
AMOTL1	-699	APOD	461	CNTN6	1620	AMY1A	2779
MUC5B	-698	DGCR9	462	EPAS1	1621	FAM101A	2780
BMP5	-697	HAAO	463	UBL4B	1622	NCRNA00028	2781
DAB1	-696	ASAP3	464	WNT11	1623	LOC654433	2782
LINGO4	-695	LOC158376	465	CXCL12	1624	UBE4B	2783
SIX6	-694	RBPMS2	466	PDZRN4	1625	CHSY3	2784
C2orf66	-693	FLRT2	467	RASL12	1626	ANXA1	2785
FBXO27	-692	WFDC1	468	NFIB	1627	CCDC88C	2786
AMPH	-691	ZG16B	469	TEX15	1628	NID2	2787
SORL1	-690	TNNC1	470	BTBD16	1629	GPR20	2788
LCN6	-689	CYP4X1	471	CCDC81	1630	OR11A1	2789
CAPSL	-688	CDH3	472	GNAL	1631	ALDH3B2	2790
IL1A	-687	STAB2	473	PYY	1632	ARPM1	2791
JPH4	-686	KCNIP3	474	COX6B2	1633	STK38	2792
TRIM34	-685	DGCR10	475	CXCR7	1634	LOC388428	2793
GABRA1	-684	ALDH1L1	476	GLT8D2	1635	MEF2B	2794
ZNF233	-683	DYNLRB2	477	RHBDL3	1636	NCRNA00158	2795
FOXN4	-682	RTN4R	478	TSPAN14	1637	KLHL38	2796
PABPC1L2A	-681	CA9	479	EMCN	1638	SSR2	2797
SPEF2	-680	DPEP1	480	SEMA3F	1639	LOC100288778	2798
IRF6	-679	NDP	481	MMP19	1640	BCMO1	2799
ZBTB37	-678	C4orf38	482	NKAIN3	1641	EFNB2	2800
CLDN20	-677	CHRD	483	TBX19	1642	PAX9	2801
THSD7B	-676	PDZK1IP1	484	C1orf135	1643	ASIP	2802
HMX2	-675	MS4A2	485	GHSR	1644	TH1L	2803
FGF14	-674	KLF17	486	AMY2B	1645	TNFAIP3	2804
ANKRD31	-673	PAPSS2	487	CCIN	1646	SC65	2805
LMX1B	-672	PDE4C	488	MLN	1647	AR	2806
RGS11	-671	IL17D	489	PSD4	1648	ZFP36L1	2807
RLTPR	-670	ZDHHC19	490	HSD17B2	1649	ALLC	2808
SYT9	-669	NTS	491	KANK4	1650	C1orf122	2809
C20orf151	-668	CLVS2	492	SULF2	1651	MRPL45	2810
ZNF365	-667	CDA	493	ARRDC2	1652	VAMP8	2811
IL9R	-666	EFHC2	494	PITPNM3	1653	TNS1	2812
NEFM	-665	CELA1	495	SIX1	1654	GCAT	2813
FRMD3	-664	CCL26	496	KANK3	1655	RPLP0P2	2814
LMBRD2	-663	FAM65C	497	ITIH2	1656	C19orf48	2815
CADPS	-662	MAL2	498	ADIPOQ	1657	BMP8A	2816
PROC	-661	TRPM5	499	CAV1	1658	OLFML1	2817
ADAMTS19	-660	GABRE	500	CPT1B	1659	TNFRSF8	2818
MFAP3L	-659	TOX2	501	GPR88	1660	GGTLC1	2819
ASRGL1	-658	POU2F3	502	ODF3L2	1661	FUCA2	2820
ANKS1B	-657	TMEM132E	503	USHBP1	1662	C10orf108	2821
NRSN1	-656	OAF	504	CECR4	1663	ACVRL1	2822
ACCN2	-655	CYP7B1	505	ASCL2	1664	SEC14L2	2823
APBA2	-654	MTP18	506	NPC1L1	1665	SIGLEC11	2824
SHROOM3	-653	PPP1R13L	507	RASL11B	1666	TCF7L2	2825
AKR1B15	-652	SIX5	508	PRRG3	1667	HYAL2	2826
NAV2	-651	C9orf170	509	ATP13A5	1668	ZMAT5	2827
BCL8	-650	IGDCC4	510	PHC2	1669	GLI4	2828
C1orf192	-649	KCND3	511	FAM167B	1670	WFIKKN2	2829
CNTN3	-648	RTN4RL1	512	NPAS1	1671	ZC3H12A	2830
TRIM7	-647	ARSJ	513	PTRF	1672	BMX	2831
DNAJC25-GNG1	-646	FREM1	514	GRAP	1673	HIC1	2832
ULBP2	-645	COL4A3	515	PTHLH	1674	CDH5	2833
HTN1	-644	PXMP2	516	TTLL12	1675	GALNT3	2834
C5orf40	-643	GP9	517	GLI3	1676	MYBPC1	2835
SCN3B	-642	SLC30A2	518	C21orf90	1677	PPIAL4D	2836
GDPD4	-641	NPPC	519	GPR124	1678	RAP1A	2837
D4S234E	-640	SLC2A4	520	PYGL	1679	HYAL4	2838
FLG2	-639	CD9	521	NXN	1680	AGTRAP	2839
TUB	-638	CCL23	522	PTCH2	1681	SNORD10	2840
EFS	-637	CLUL1	523	C8orf79	1682	RPL13AP20	2841
LOC151658	-636	CYSLTR2	524	FERMT1	1683	ANKRD13C	2842
ETV1	-635	KLKP1	525	PAX8	1684	POLR2J2	2843
ADAM32	-634	KCNT2	526	WWC2	1685	SNORA20	2844
RAB9B	-633	MMEL1	527	DYNLT1	1686	PRDM6	2845
PPARGC1B	-632	BAMBI	528	APOLD1	1687	WSCD2	2846
B3GALT5	-631	LRIG3	529	C1orf150	1688	RALGPS2	2847
FOXP3	-630	C8orf51	530	LOC150381	1689	UBAC2	2848
MYCN	-629	SLC45A3	531	RIPPLY1	1690	TNIP1	2849
FAM105A	-628	LEMD1	532	COBLL1	1691	BCL10	2850
MRGPRX3	-627	FOLR1	533	C22orf34	1692	KIAA0040	2851
CACNA1B	-626	IL18R1	534	KLHL14	1693	SCN4A	2852
CRYBB1	-625	NETO1	535	GABRR2	1694	FNDC3B	2853
PRR16	-624	LOC401093	536	PLEKHG2	1695	ABCC5	2854
CADM2	-623	CD1D	537	G0S2	1696	LRRC25	2855
GPR68	-622	PGR	538	LRG1	1697	LOC152225	2856
GPR62	-621	EFHD2	539	CXCL14	1698	PPIH	2857
PRSS45	-620	C1orf186	540	RPL39L	1699	R3HCC1	2858
VIP	-619	AQP1	541	NCRNA00162	1700	HIST1H4B	2859
DOC2A	-618	COL9A1	542	FOXF1	1701	REEP4	2860

CPEB1	-617	C5orf62	543	CIDEA	1702	DSCR8	2861
DTX1	-616	EDNRA	544	CDH1	1703	PPCS	2862
KLHL35	-615	PLIN5	545	NFKB2	1704	CEACAM21	2863
BAI3	-614	SPESP1	546	OIP5	1705	DLL4	2864
AKR1C3	-613	LOC100129066	547	CLEC18A	1706	HSPA2	2865
DIRAS2	-612	TMEM133	548	LOC340508	1707	PYGM	2866
RP1-177G6.2	-611	SYT12	549	LHFP	1708	RERE	2867
CCDC67	-610	GATA6	550	FBLN1	1709	PPFIA4	2868
