# Supplementary material for: Identification of Clinical Relevant Molecular Subtypes of Pheochromocytoma
Source: Front Endocrinol (Lausanne). 2021 Jun 21;12:605797. doi: 10.3389/fendo.2021.605797 (PMC8256389; doi:10.3389/fendo.2021.605797)
Supplement: Supplementary file 3 [file DataSheet_3.pdf]

**Table 3. Biological process enriched in each subtypes of PCC. Enriched GO BP terms by genes over-expressed in Subtype I PCC**

| Category | Term | Count | % | PValue | Genes | List Total | Pop Hits | Pop Total | Fold Enric | Bonferror | Benjamini | FDR |
|---|---|---|---|---|---|---|---|---|---|---|---|---|
| GOTERM_BP_DIRECT | GO:0007399~nervous system development | 45 | 0.038674 | 1.14E-13 | GDA, FUT9, GRIK1, PCDHA2, PCI | 714 | 287 | 16792 | 3.687524 | 2.86E-10 | 2.86E-10 | 2.01E-10 |
| GOTERM_BP_DIRECT | GO:0007268~chemical synaptic transmission | 39 | 0.033517 | 2.03E-12 | OPRM1, GRIK1, SLC6A2, NPY2R, | 714 | 240 | 16792 | 3.821709 | 5.10E-09 | 2.55E-09 | 3.59E-09 |
| GOTERM_BP_DIRECT | GO:0007218~neuropeptide signaling pathway | 24 | 0.020626 | 2.76E-11 | OPRM1, NPB, GLRA1, OPRL1, RX | 714 | 101 | 16792 | 5.588485 | 6.92E-08 | 2.31E-08 | 4.87E-08 |
| GOTERM_BP_DIRECT | GO:0034765~regulation of ion transmembrane | 22 | 0.018907 | 7.28E-09 | KCNH1, CALHM1, SCN3A, KCNB | 714 | 111 | 16792 | 4.661266 | 1.83E-05 | 4.57E-06 | 1.28E-05 |
| GOTERM_BP_DIRECT | GO:0060078~regulation of postsynaptic membr | 10 | 0.008594 | 1.29E-07 | SCN2B, SCN3A, FGF14, SCN3B, S | 714 | 22 | 16792 | 10.69009 | 3.24E-04 | 6.47E-05 | 2.28E-04 |
| GOTERM_BP_DIRECT | GO:0019233~sensory perception of pain | 14 | 0.012032 | 1.79E-07 | OPRM1, GIP, SCN3B, OPRL1, KCI | 714 | 52 | 16792 | 6.331825 | 4.50E-04 | 7.50E-05 | 3.17E-04 |
| GOTERM_BP_DIRECT | GO:0060291~long-term synaptic potentiation | 11 | 0.009454 | 2.87E-06 | GIP, SLC8A2, S100B, TNR, SLC24 | 714 | 38 | 16792 | 6.807902 | 0.007175 | 0.001028 | 0.005065 |
| GOTERM_BP_DIRECT | GO:0071805~potassium ion transmembrane tra | 19 | 0.016329 | 3.43E-06 | KCNH1, KCNC1, SLC9A5, KCNAB | 714 | 121 | 16792 | 3.692942 | 0.008573 | 0.001076 | 0.006056 |
| GOTERM_BP_DIRECT | GO:0007612~learning | 13 | 0.011172 | 3.82E-06 | JPH4, ARC, SLC8A2, BCHE, NRXN | 714 | 57 | 16792 | 5.363802 | 0.00953 | 0.001063 | 0.006735 |
| GOTERM_BP_DIRECT | GO:0008306~associative learning | 9 | 0.007735 | 4.08E-06 | DRD2, TNR, DRD5, NEUROD2, T/ | 714 | 24 | 16792 | 8.819328 | 0.010182 | 0.001023 | 0.007199 |
| GOTERM_BP_DIRECT | GO:0050808~synapse organization | 10 | 0.008594 | 6.57E-06 | ATP2B2, RAB39B, LRRTM4, PAK | 714 | 33 | 16792 | 7.126729 | 0.01635 | 0.001498 | 0.011595 |
| GOTERM_BP_DIRECT | GO:0007417~central nervous system developme | 18 | 0.015469 | 1.24E-05 | HAPLN4, GRIK1, BCAN, DSCAML | 714 | 120 | 16792 | 3.527731 | 0.030717 | 0.002597 | 0.021943 |
| GOTERM_BP_DIRECT | GO:0086010~membrane depolarization during | 9 | 0.007735 | 1.48E-05 | SCN3A, SCN3B, SCN2A, SCN9A, | 714 | 28 | 16792 | 7.559424 | 0.036507 | 0.002857 | 0.026156 |
| GOTERM_BP_DIRECT | GO:0007631~feeding behavior | 10 | 0.008594 | 2.30E-05 | CALCA, HCRTR1, NPY, DRD2, NP | 714 | 38 | 16792 | 6.189002 | 0.055965 | 0.004105 | 0.040502 |
| GOTERM_BP_DIRECT | GO:0007626~locomotory behavior | 14 | 0.012032 | 5.06E-05 | OPRM1, DRD2, NPY2R, TH, ASTN | 714 | 84 | 16792 | 3.919701 | 0.119177 | 0.008424 | 0.089221 |
| GOTERM_BP_DIRECT | GO:0007613~memory | 12 | 0.010313 | 5.26E-05 | HRH1, GIP, SLC8A2, S100B, SLC2 | 714 | 62 | 16792 | 4.551911 | 0.123693 | 0.008218 | 0.092833 |
| GOTERM_BP_DIRECT | GO:0007409~axonogenesis | 15 | 0.012891 | 6.52E-05 | DRD2, NKX2-8, SPTBN4, NTNG1, | 714 | 98 | 16792 | 3.599726 | 0.150955 | 0.00958 | 0.11504 |
| GOTERM_BP_DIRECT | GO:0007187~G-protein coupled receptor signali | 10 | 0.008594 | 1.14E-04 | OPRM1, SSTR3, NPY, HRH3, SSTI | 714 | 46 | 16792 | 5.112654 | 0.24966 | 0.015831 | 0.201832 |
| GOTERM_BP_DIRECT | GO:0019228~neuronal action potential | 8 | 0.006875 | 1.31E-04 | SCN3A, KCNA2, SCN2A, SCN9A, | 714 | 28 | 16792 | 6.719488 | 0.280006 | 0.017142 | 0.230808 |
| GOTERM_BP_DIRECT | GO:0006813~potassium ion transport | 13 | 0.011172 | 1.69E-04 | KCNH1, KCNJ5, KCNC1, KCNQ3, | 714 | 82 | 16792 | 3.728496 | 0.346281 | 0.02103 | 0.298552 |
| GOTERM_BP_DIRECT | GO:0007269~neurotransmitter secretion | 10 | 0.008594 | 2.62E-04 | SLC32A1, PPFIA2, SYN1, HRH3, N | 714 | 51 | 16792 | 4.611413 | 0.481256 | 0.030771 | 0.460607 |
| GOTERM_BP_DIRECT | GO:0071395~cellular response to jasmonic acid | 4 | 0.003438 | 2.95E-04 | AKR1C3, AKR1C2, AKR1C4, AKR1 | 714 | 4 | 16792 | 23.51821 | 0.523378 | 0.033122 | 0.519883 |
| GOTERM_BP_DIRECT | GO:0007156~homophilic cell adhesion via plasm | 18 | 0.015469 | 4.02E-04 | RET, CADM4, PCDHA9, PCDHA2, | 714 | 158 | 16792 | 2.679289 | 0.635064 | 0.042881 | 0.706541 |
| GOTERM_BP_DIRECT | GO:0007158~neuron cell-cell adhesion | 6 | 0.005156 | 4.02E-04 | NCAM2, RET, NRXN3, TNR, NLGI | 714 | 16 | 16792 | 8.819328 | 0.63544 | 0.041173 | 0.707261 |
| GOTERM_BP_DIRECT | GO:0048265~response to pain | 6 | 0.005156 | 5.50E-04 | CALCA, RET, SLC6A2, TAC1, RELN | 714 | 17 | 16792 | 8.300544 | 0.748406 | 0.053702 | 0.965949 |
| GOTERM_BP_DIRECT | GO:0001508~action potential | 6 | 0.005156 | 5.50E-04 | GJD2, GLRA1, KCNB1, CHRNB4, | 714 | 17 | 16792 | 8.300544 | 0.748406 | 0.053702 | 0.965949 |
| GOTERM_BP_DIRECT | GO:0017158~regulation of calcium ion-depende | 8 | 0.006875 | 5.74E-04 | DOC2A, SYT2, SYT14, SYT9, C2CI | 714 | 35 | 16792 | 5.37559 | 0.763283 | 0.053911 | 1.0084 |
| GOTERM_BP_DIRECT | GO:0051968~positive regulation of synaptic trar | 6 | 0.005156 | 9.63E-04 | TNR, NTRK1, NLGN1, RELN, SHA | 714 | 19 | 16792 | 7.426802 | 0.910791 | 0.085621 | 1.685587 |
| GOTERM_BP_DIRECT | GO:0035725~sodium ion transmembrane trans | 11 | 0.009454 | 9.89E-04 | SLC8A2, SCN2B, SCN3A, SCN3B, | 714 | 73 | 16792 | 3.543839 | 0.916481 | 0.08485 | 1.73115 |
| GOTERM_BP_DIRECT | GO:0048791~calcium ion-regulated exocytosis o | 8 | 0.006875 | 0.001133 | DOC2A, SYT2, SYT14, SYT9, C2CI | 714 | 39 | 16792 | 4.824248 | 0.941888 | 0.093457 | 1.981537 |
| GOTERM_BP_DIRECT | GO:0050890~cognition | 8 | 0.006875 | 0.001133 | GPR155, HRH3, LHCGR, CHRNA7 | 714 | 39 | 16792 | 4.824248 | 0.941888 | 0.093457 | 1.981537 |
| GOTERM_BP_DIRECT | GO:0051965~positive regulation of synapse ass | 10 | 0.008594 | 0.001152 | FLRT3, LINGO2, FLRT1, CLSTN2, | 714 | 62 | 16792 | 3.793259 | 0.944478 | 0.091868 | 2.012969 |
| GOTERM_BP_DIRECT | GO:0008344~adult locomotory behavior | 9 | 0.007735 | 0.001461 | GIP, ATP1A3, INPP5F, ADAM22, | 714 | 52 | 16792 | 4.070459 | 0.974453 | 0.111568 | 2.546531 |
| GOTERM_BP_DIRECT | GO:0001764~neuron migration | 13 | 0.011172 | 0.001645 | NAV1, DYX1C1, MDGA1, TBX20, | 714 | 105 | 16792 | 2.911778 | 0.98392 | 0.121086 | 2.863378 |
| GOTERM_BP_DIRECT | GO:0035418~protein localization to synapse | 5 | 0.004297 | 0.001695 | LRRTM1, NPHS1, NLGN1, BSN, R | 714 | 13 | 16792 | 9.045464 | 0.985829 | 0.121015 | 2.949695 |
| GOTERM_BP_DIRECT | GO:0007628~adult walking behavior | 7 | 0.006016 | 0.001701 | DAB1, GLRA1, DRD2, SPTBN4, CI | 714 | 31 | 16792 | 5.310563 | 0.986044 | 0.11807 | 2.960123 |
| GOTERM_BP_DIRECT | GO:0051480~regulation of cytosolic calcium ion | 7 | 0.006016 | 0.00202 | CALCA, HCRTR1, ATP2B2, TRPC5 | 714 | 32 | 16792 | 5.144608 | 0.993733 | 0.134915 | 3.505061 |
| GOTERM_BP_DIRECT | GO:0035235~ionotropic glutamate receptor sigr | 6 | 0.005156 | 0.002952 | GRIA2, GRIK1, GRIA1, GRID2, AT | 714 | 24 | 16792 | 5.879552 | 0.999399 | 0.18619 | 5.083373 |
| GOTERM_BP_DIRECT | GO:0036158~outer dynein arm assembly | 5 | 0.004297 | 0.003024 | LRRC6, DNAI1, DYX1C1, ZMYND | 714 | 15 | 16792 | 7.839402 | 0.999499 | 0.185656 | 5.204632 |
| GOTERM_BP_DIRECT | GO:0007215~glutamate receptor signaling path | 5 | 0.004297 | 0.003024 | GRIK1, SSTR1, KCNB1, GRID2, GI | 714 | 15 | 16792 | 7.839402 | 0.999499 | 0.185656 | 5.204632 |
| GOTERM_BP_DIRECT | GO:0007411~axon guidance | 16 | 0.013751 | 0.003198 | FLRT3, NRXN3, KIF5A, SPTBN4, F | 714 | 159 | 16792 | 2.366612 | 0.999677 | 0.190622 | 5.496095 |
| GOTERM_BP_DIRECT | GO:0033344~cholesterol efflux | 6 | 0.005156 | 0.003563 | ABCG8, APOA2, APOB, ABCG5, | 714 | 25 | 16792 | 5.64437 | 0.999871 | 0.205156 | 6.104492 |
| GOTERM_BP_DIRECT | GO:0044597~daunorubicin metabolic process | 4 | 0.003438 | 0.003637 | AKR1C3, AKR1C2, AKR1C4, AKR1 | 714 | 8 | 16792 | 11.7591 | 0.999893 | 0.204299 | 6.227584 |
| GOTERM_BP_DIRECT | GO:0044598~doxorubicin metabolic process | 4 | 0.003438 | 0.003637 | AKR1C3, AKR1C2, AKR1C4, AKR1 | 714 | 8 | 16792 | 11.7591 | 0.999893 | 0.204299 | 6.227584 |
| GOTERM_BP_DIRECT | GO:0070588~calcium ion transmembrane trans | 13 | 0.011172 | 0.004678 | OPRM1, CALHM1, SLC8A2, TRPC | 714 | 119 | 16792 | 2.569216 | 0.999992 | 0.249464 | 7.942741 |
| GOTERM_BP_DIRECT | GO:0097477~lateral motor column neuron migr | 3 | 0.002578 | 0.005249 | DAB1, LHX1, RELN | 714 | 3 | 16792 | 23.51821 | 0.999998 | 0.269766 | 8.869645 |
| GOTERM_BP_DIRECT | GO:0046426~negative regulation of JAK-STAT ca | 7 | 0.006016 | 0.006421 | FLRT3, FLRT1, PODNL1, DAB1, LI | 714 | 40 | 16792 | 4.115686 | 1 | 0.313292 | 10.74554 |
| GOTERM_BP_DIRECT | GO:0090129~positive regulation of synapse mat | 4 | 0.003438 | 0.007312 | NRXN3, NEUROD2, RELN, CAMK | 714 | 10 | 16792 | 9.407283 | 1 | 0.341965 | 12.14856 |
| GOTERM_BP_DIRECT | GO:0019732~antifungal humoral response | 4 | 0.003438 | 0.007312 | CALCA, VIP, NPY, TAC1 | 714 | 10 | 16792 | 9.407283 | 1 | 0.341965 | 12.14856 |
| GOTERM_BP_DIRECT | GO:0007155~cell adhesion | 32 | 0.027501 | 0.007529 | PLXNC1, PCDHA2, PCDHA3, BCA | 714 | 459 | 16792 | 1.639614 | 1 | 0.343853 | 12.48641 |
| GOTERM_BP_DIRECT | GO:0006811~ion transport | 13 | 0.011172 | 0.007814 | SLC32A1, LHFPL5, SLC8A2, TMC | 714 | 127 | 16792 | 2.407376 | 1 | 0.348119 | 12.92937 |
| GOTERM_BP_DIRECT | GO:0045776~negative regulation of blood press | 6 | 0.005156 | 0.008025 | CALCA, OPRL1, DRD2, DRD5, NO | 714 | 30 | 16792 | 4.703641 | 1 | 0.349577 | 13.25525 |
| GOTERM_BP_DIRECT | GO:0034220~ion transmembrane transport | 18 | 0.015469 | 0.008323 | GABRG2, GABRG3, GLRA1, GRIK | 714 | 210 | 16792 | 2.015846 | 1 | 0.353943 | 13.71377 |
| GOTERM_BP_DIRECT | GO:0048149~behavioral response to ethanol | 4 | 0.003438 | 0.00974 | OPRM1, DRD2, CHRNA7, CHRFA | 714 | 11 | 16792 | 8.552075 | 1 | 0.394191 | 15.86461 |
| GOTERM_BP_DIRECT | GO:0003272~endocardial cushion formation | 3 | 0.002578 | 0.010203 | TBX20, BMP7, BMP5 | 714 | 4 | 16792 | 17.63866 | 1 | 0.40226 | 16.55511 |
| GOTERM_BP_DIRECT | GO:0021702~cerebellar Purkinje cell differentiat | 4 | 0.003438 | 0.012582 | ATP2B2, LHX1, CEND1, CACNA1 | 714 | 12 | 16792 | 7.839402 | 1 | 0.463632 | 20.02588 |
| GOTERM_BP_DIRECT | GO:0048666~neuron development | 7 | 0.006016 | 0.012653 | ISL2, PACSIN1, ATP8A2, NEUROI | 714 | 46 | 16792 | 3.578858 | 1 | 0.459031 | 20.12639 |
| GOTERM_BP_DIRECT | GO:0010842~retina layer formation | 5 | 0.004297 | 0.012805 | LHX1, TFAP2B, ATP8A2, ARL6, F | 714 | 22 | 16792 | 5.345047 | 1 | 0.456708 | 20.3437 |
| GOTERM_BP_DIRECT | GO:0010976~positive regulation of neuron proje | 10 | 0.008594 | 0.013159 | RET, STMN2, NTRK1, PTK6, ATP | 714 | 89 | 16792 | 2.642495 | 1 | 0.459614 | 20.84623 |
| GOTERM_BP_DIRECT | GO:0042391~regulation of membrane potential | 9 | 0.007735 | 0.014005 | KCNH1, SLC26A5, GLRA1, CHRN | 714 | 75 | 16792 | 2.822185 | 1 | 0.474505 | 22.0355 |
| GOTERM_BP_DIRECT | GO:0007416~synapse assembly | 8 | 0.006875 | 0.01442 | FLRT3, NRXN3, DRD2, NLGN1, B | 714 | 61 | 16792 | 3.084355 | 1 | 0.478365 | 22.61263 |
| GOTERM_BP_DIRECT | GO:0030509~BMP signaling pathway | 9 | 0.007735 | 0.01509 | ZCCHC12, GDF1, GDF10, ZCCHC | 714 | 76 | 16792 | 2.785051 | 1 | 0.48793 | 23.53557 |
| GOTERM_BP_DIRECT | GO:0001878~response to yeast | 4 | 0.003438 | 0.015849 | CALCA, VIP, NPY, TAC1 | 714 | 13 | 16792 | 7.236371 | 1 | 0.498966 | 24.5682 |
| GOTERM_BP_DIRECT | GO:0007600~sensory perception | 4 | 0.003438 | 0.015849 | OPRM1, PENK, OPRL1, PDYN | 714 | 13 | 16792 | 7.236371 | 1 | 0.498966 | 24.5682 |
| GOTERM_BP_DIRECT | GO:0050996~positive regulation of lipid catabol | 3 | 0.002578 | 0.016528 | APOA2, APOA5, PRKCE | 714 | 5 | 16792 | 14.11092 | 1 | 0.507729 | 25.48162 |
| GOTERM_BP_DIRECT | GO:0035336~long-chain fatty-acyl-CoA metabol | 3 | 0.002578 | 0.016528 | FAR2, DGAT2, THEM5 | 714 | 5 | 16792 | 14.11092 | 1 | 0.507729 | 25.48162 |
| GOTERM_BP_DIRECT | GO:0045085~negative regulation of interleukin- | 3 | 0.002578 | 0.016528 | SFTPD, FOXP3, LAG3 | 714 | 5 | 16792 | 14.11092 | 1 | 0.507729 | 25.48162 |
| GOTERM_BP_DIRECT | GO:0006874~cellular calcium ion homeostasis | 10 | 0.008594 | 0.017172 | CALCB, ATP2B3, SLC8A2, ANK2, | 714 | 93 | 16792 | 2.528839 | 1 | 0.515343 | 26.33841 |
| GOTERM_BP_DIRECT | GO:0051924~regulation of calcium ion transpor | 5 | 0.004297 | 0.017399 | ANK2, CAMK2B, CACNA2D3, OP | 714 | 24 | 16792 | 4.899627 | 1 | 0.5142 | 26.63864 |
| GOTERM_BP_DIRECT | GO:0051260~protein homooligomerization | 15 | 0.012891 | 0.018856 | CALHM1, KCNC1, GLRA1, KCNB2 | 714 | 177 | 16792 | 1.993068 | 1 | 0.537152 | 28.53466 |
| GOTERM_BP_DIRECT | GO:0021549~cerebellum development | 6 | 0.005156 | 0.019259 | KCNC1, SSTR3, SSTR1, LHX1, PTI | 714 | 37 | 16792 | 3.813763 | 1 | 0.539065 | 29.0512 |
| GOTERM_BP_DIRECT | GO:0007271~synaptic transmission, cholinergic | 6 | 0.005156 | 0.019259 | CHRM5, CHRM4, HRH3, CHRNB | 714 | 37 | 16792 | 3.813763 | 1 | 0.539065 | 29.0512 |
| GOTERM_BP_DIRECT | GO:0071277~cellular response to calcium ion | 7 | 0.006016 | 0.020378 | KCNH1, AKR1C3, GUCA1A, CPNE | 714 | 51 | 16792 | 3.227989 | 1 | 0.553869 | 30.46602 |
| GOTERM_BP_DIRECT | GO:0051930~regulation of sensory perception o | 5 | 0.004297 | 0.022903 | OPRM1, NPY2R, TMEM100, ADC | 714 | 26 | 16792 | 4.522732 | 1 | 0.591127 | 33.56226 |
| GOTERM_BP_DIRECT | GO:0042755~eating behavior | 5 | 0.004297 | 0.022903 | OPRM1, OPRL1, TH, ATP8A2, OF | 714 | 26 | 16792 | 4.522732 | 1 | 0.591127 | 33.56226 |
| GOTERM_BP_DIRECT | GO:0001964~startle response | 4 | 0.003438 | 0.023676 | KCNH1, GLRA1, PENK, NRG1 | 714 | 15 | 16792 | 6.271522 | 1 | 0.597823 | 34.48302 |
| GOTERM_BP_DIRECT | GO:0086015~SA node cell action potential | 3 | 0.002578 | 0.024099 | ANK2, SCN3B, SCN5A | 714 | 6 | 16792 | 11.7591 | 1 | 0.598891 | 34.98297 |
| GOTERM_BP_DIRECT | GO:0014061~regulation of norepinephrine secre | 3 | 0.002578 | 0.024099 | HRH3, CHRNA7, CHRFAM7A | 714 | 6 | 16792 | 11.7591 | 1 | 0.598891 | 34.98297 |
| GOTERM_BP_DIRECT | GO:0021517~ventral spinal cord development | 3 | 0.002578 | 0.024099 | DAB1, LHX1, RELN | 714 | 6 | 16792 | 11.7591 | 1 | 0.598891 | 34.98297 |
| GOTERM_BP_DIRECT | GO:0007601~visual perception | 16 | 0.013751 | 0.024671 | GJD2, GUCA1A, SLC45A2, GLRA1 | 714 | 201 | 16792 | 1.872096 | 1 | 0.602165 | 35.6522 |
| GOTERM_BP_DIRECT | GO:0003341~cilium movement | 5 | 0.004297 | 0.026009 | LRRC6, HYDIN, DNAI1, DYX1C1, | 714 | 27 | 16792 | 4.355224 | 1 | 0.616438 | 37.19168 |
| GOTERM_BP_DIRECT | GO:0043547~positive regulation of GTPase activ | 35 | 0.03008 | 0.026171 | ARHGDIG, ODAM, FGF16, RASGI | 714 | 565 | 16792 | 1.45688 | 1 | 0.613472 | 37.37656 |
| GOTERM_BP_DIRECT | GO:0009566~fertilization | 6 | 0.005156 | 0.02622 | APOB, TDRKH, SPTBN4, SPEF2, S | 714 | 40 | 16792 | 3.527731 | 1 | 0.608953 | 37.43172 |
| GOTERM_BP_DIRECT | GO:0060292~long term synaptic depression | 4 | 0.003438 | 0.02824 | GRIA1, SLC24A2, DRD5, SHANK2 | 714 | 16 | 16792 | 5.879552 | 1 | 0.631473 | 39.68298 |
| GOTERM_BP_DIRECT | GO:1900006~positive regulation of dendrite dev | 4 | 0.003438 | 0.02824 | PACSIN1, BMP7, SEZ6, BMP5 | 714 | 16 | 16792 | 5.879552 | 1 | 0.631473 | 39.68298 |
| GOTERM_BP_DIRECT | GO:0007616~long-term memory | 5 | 0.004297 | 0.029354 | CAMK4, DRD2, GRIA1, TAC1, REI | 714 | 28 | 16792 | 4.19968 | 1 | 0.640849 | 40.8924 |
| GOTERM_BP_DIRECT | GO:0016358~dendrite development | 5 | 0.004297 | 0.029354 | FLRT1, DAB1, PAK3, RELN, BMP7 | 714 | 28 | 16792 | 4.19968 | 1 | 0.640849 | 40.8924 |
| GOTERM_BP_DIRECT | GO:0007420~brain development | 15 | 0.012891 | 0.032158 | DLX2, HMX2, ECE2, CA10, CADM | 714 | 190 | 16792 | 1.856701 | 1 | 0.669867 | 43.83433 |
| GOTERM_BP_DIRECT | GO:0038003~opioid receptor signaling pathway | 3 | 0.002578 | 0.0328 | OPRL1, NPBWR2, OPRD1 | 714 | 7 | 16792 | 10.07923 | 1 | 0.672309 | 44.48834 |
| GOTERM_BP_DIRECT | GO:0000160~phosphorelay signal transduction | 3 | 0.002578 | 0.0328 | KCNH1, KCNH4, KCNH5 | 714 | 7 | 16792 | 10.07923 | 1 | 0.672309 | 44.48834 |
| GOTERM_BP_DIRECT | GO:0003344~pericardium morphogenesis | 3 | 0.002578 | 0.0328 | TBX20, BMP7, BMP5 | 714 | 7 | 16792 | 10.07923 | 1 | 0.672309 | 44.48834 |
| GOTERM_BP_DIRECT | GO:0007197~adenylate cyclase-inhibiting G-pro | 3 | 0.002578 | 0.0328 | CHRM5, CHRM4, HRH3 | 714 | 7 | 16792 | 10.07923 | 1 | 0.672309 | 44.48834 |
| GOTERM_BP_DIRECT | GO:0001662~behavioral fear response | 5 | 0.004297 | 0.032943 | PENK, NPY2R, NEUROD2, MDK, | 714 | 29 | 16792 | 4.054863 | 1 | 0.66908 | 44.63279 |
| GOTERM_BP_DIRECT | GO:0021537~telencephalon development | 4 | 0.003438 | 0.033235 | LHX1, SIX3, NRGN, SCN5A | 714 | 17 | 16792 | 5.533696 | 1 | 0.667579 | 44.92701 |
| GOTERM_BP_DIRECT | GO:0007165~signal transduction | 63 | 0.054143 | 0.035256 | ZNF536, FGF14, FGF16, SORL1, | 714 | 1161 | 16792 | 1.276182 | 1 | 0.6848 | 46.92387 |
| GOTERM_BP_DIRECT | GO:0001654~eye development | 5 | 0.004297 | 0.036777 | SALL2, SMOC1, SIX3, BMPR1B, N | 714 | 30 | 16792 | 3.919701 | 1 | 0.695789 | 48.38108 |
| GOTERM_BP_DIRECT | GO:0071773~cellular response to BMP stimulus | 5 | 0.004297 | 0.036777 | SPINT2, TMEM100, ADAMTS12, | 714 | 30 | 16792 | 3.919701 | 1 | 0.695789 | 48.38108 |
| GOTERM_BP_DIRECT | GO:0030154~cell differentiation | 29 | 0.024923 | 0.037649 | ETV7, HMX2, CADM1, NELL1, OI | 714 | 462 | 16792 | 1.476251 | 1 | 0.699876 | 49.19953 |
| GOTERM_BP_DIRECT | GO:0006906~vesicle fusion | 7 | 0.006016 | 0.038477 | DOC2A, SYT2, SYT14, SYT9, C2CI | 714 | 59 | 16792 | 2.790296 | 1 | 0.703403 | 49.96589 |
| GOTERM_BP_DIRECT | GO:0051412~response to corticosterone | 4 | 0.003438 | 0.038657 | UCN3, MAOB, TH, NEFL | 714 | 18 | 16792 | 5.226268 | 1 | 0.70069 | 50.13065 |
| GOTERM_BP_DIRECT | GO:0007623~circadian rhythm | 8 | 0.006875 | 0.039692 | PROK2, HTR7, NTRK1, HS3ST2, P | 714 | 75 | 16792 | 2.508609 | 1 | 0.706042 | 51.07011 |

| | | | | | | | | | | | | |
|---|---|---|---|---|---|---|---|---|---|---|---|---|
| GOTERM_BP_DIRECT | GO:0021952~central nervous system projection | 3 | 0.002578 | 0.042522 | SPTBN4, DCX, DCLK1 | 714 | 8 | 16792 | 8.819328 | 1 | 0.726889 | 53.55287 |
| GOTERM_BP_DIRECT | GO:0010269~response to selenium ion | 3 | 0.002578 | 0.042522 | APOB, GIP, MAOB | 714 | 8 | 16792 | 8.819328 | 1 | 0.726889 | 53.55287 |
| GOTERM_BP_DIRECT | GO:0007207~phospholipase C-activating G-prot | 3 | 0.002578 | 0.042522 | CHRM5, CHRM4, HRH3 | 714 | 8 | 16792 | 8.819328 | 1 | 0.726889 | 53.55287 |
| GOTERM_BP_DIRECT | GO:0060124~positive regulation of growth horn | 3 | 0.002578 | 0.042522 | GHRH, DRD2, ADCYAP1 | 714 | 8 | 16792 | 8.819328 | 1 | 0.726889 | 53.55287 |
| GOTERM_BP_DIRECT | GO:0060371~regulation of atrial cardiac muscle | 3 | 0.002578 | 0.042522 | SCN2B, SCN3B, SCN5A | 714 | 8 | 16792 | 8.819328 | 1 | 0.726889 | 53.55287 |
| GOTERM_BP_DIRECT | GO:0001523~retinoid metabolic process | 7 | 0.006016 | 0.044212 | AKR1C3, APOA2, APOB, AKR1C4 | 714 | 61 | 16792 | 2.698811 | 1 | 0.736782 | 54.97912 |
| GOTERM_BP_DIRECT | GO:0050885~neuromuscular process controlling | 6 | 0.005156 | 0.044483 | ATP2B2, JPH4, TNR, NEFL, CACN | 714 | 46 | 16792 | 3.067592 | 1 | 0.734864 | 55.20356 |
| GOTERM_BP_DIRECT | GO:0008045~motor neuron axon guidance | 4 | 0.003438 | 0.044498 | ALCAM, RNF165, LHX1, LHX9 | 714 | 19 | 16792 | 4.951202 | 1 | 0.730912 | 55.21626 |
| GOTERM_BP_DIRECT | GO:0031290~retinal ganglion cell axon guidance | 4 | 0.003438 | 0.044498 | ALCAM, ISL2, EFNA5, BMPR1B | 714 | 19 | 16792 | 4.951202 | 1 | 0.730912 | 55.21626 |
| GOTERM_BP_DIRECT | GO:0009953~dorsal/ventral pattern formation | 5 | 0.004297 | 0.045182 | NOTO, LHX1, TBX20, DSCAML1, | 714 | 32 | 16792 | 3.67472 | 1 | 0.732382 | 55.77807 |
| GOTERM_BP_DIRECT | GO:0048167~regulation of synaptic plasticity | 5 | 0.004297 | 0.045182 | ATP2B2, JPH4, HRH1, FGF14, VG | 714 | 32 | 16792 | 3.67472 | 1 | 0.732382 | 55.77807 |
| GOTERM_BP_DIRECT | GO:0019226~transmission of nerve impulse | 4 | 0.003438 | 0.05075 | CHRM5, DRD5, SPTBN4, CACNA: | 714 | 20 | 16792 | 4.703641 | 1 | 0.769681 | 60.11553 |
| GOTERM_BP_DIRECT | GO:0007169~transmembrane receptor protein t | 9 | 0.007735 | 0.051318 | IGF1R, RET, DOK5, NTRK1, PTK6, | 714 | 96 | 16792 | 2.204832 | 1 | 0.76976 | 60.53397 |
| GOTERM_BP_DIRECT | GO:0030299~intestinal cholesterol absorption | 3 | 0.002578 | 0.053161 | ABCG8, ABCG5, AKR1C1 | 714 | 9 | 16792 | 7.839402 | 1 | 0.778232 | 61.86576 |
| GOTERM_BP_DIRECT | GO:0042448~progesterone metabolic process | 3 | 0.002578 | 0.053161 | AKR1C3, AKR1C2, AKR1C1 | 714 | 9 | 16792 | 7.839402 | 1 | 0.778232 | 61.86576 |
| GOTERM_BP_DIRECT | GO:0086014~atrial cardiac muscle cell action po | 3 | 0.002578 | 0.053161 | ANK2, SCN3B, SCN5A | 714 | 9 | 16792 | 7.839402 | 1 | 0.778232 | 61.86576 |
| GOTERM_BP_DIRECT | GO:0097120~receptor localization to synapse | 3 | 0.002578 | 0.053161 | NLGN1, RELN, DLG2 | 714 | 9 | 16792 | 7.839402 | 1 | 0.778232 | 61.86576 |
| GOTERM_BP_DIRECT | GO:0051967~negative regulation of synaptic tra | 3 | 0.002578 | 0.053161 | DRD2, NPY2R, ADORA1 | 714 | 9 | 16792 | 7.839402 | 1 | 0.778232 | 61.86576 |
| GOTERM_BP_DIRECT | GO:0010579~positive regulation of adenylate cy | 3 | 0.002578 | 0.053161 | VIP, DRD5, ADCYAP1 | 714 | 9 | 16792 | 7.839402 | 1 | 0.778232 | 61.86576 |
| GOTERM_BP_DIRECT | GO:0035095~behavioral response to nicotine | 3 | 0.002578 | 0.053161 | CHRNB4, CHRNA7, CHRFAM7A | 714 | 9 | 16792 | 7.839402 | 1 | 0.778232 | 61.86576 |
| GOTERM_BP_DIRECT | GO:0042632~cholesterol homeostasis | 7 | 0.006016 | 0.053767 | ABCG8, APOA2, APOB, ABCG5, [ | 714 | 64 | 16792 | 2.572304 | 1 | 0.778475 | 62.29435 |
| GOTERM_BP_DIRECT | GO:0009968~negative regulation of signal trans | 5 | 0.004297 | 0.054568 | RGS22, RGS11, PTK6, RGS17, RG | 714 | 34 | 16792 | 3.45856 | 1 | 0.779938 | 62.85334 |
| GOTERM_BP_DIRECT | GO:0006814~sodium ion transport | 8 | 0.006875 | 0.056177 | SCN3A, SCN3B, SCN2A, SCN9A, | 714 | 81 | 16792 | 2.322786 | 1 | 0.786309 | 63.9538 |
| GOTERM_BP_DIRECT | GO:0051384~response to glucocorticoid | 7 | 0.006016 | 0.057209 | APOA2, S100B, BCHE, CDO1, MI | 714 | 65 | 16792 | 2.53273 | 1 | 0.788994 | 64.643 |
| GOTERM_BP_DIRECT | GO:0008217~regulation of blood pressure | 7 | 0.006016 | 0.057209 | CALCA, RENBP, TRHDE, NPY, TAC | 714 | 65 | 16792 | 2.53273 | 1 | 0.788994 | 64.643 |
| GOTERM_BP_DIRECT | GO:0032094~response to food | 4 | 0.003438 | 0.057403 | OPRM1, GHRH, CHRNA7, CHRFA | 714 | 21 | 16792 | 4.479659 | 1 | 0.786697 | 64.77119 |
| GOTERM_BP_DIRECT | GO:0045921~positive regulation of exocytosis | 4 | 0.003438 | 0.057403 | UNC13D, VSNL1, EXPH5, RAB27 | 714 | 21 | 16792 | 4.479659 | 1 | 0.786697 | 64.77119 |
| GOTERM_BP_DIRECT | GO:0007204~positive regulation of cytosolic calc | 11 | 0.009454 | 0.059303 | OPRM1, CALCA, PROK2, OPRL1, | 714 | 134 | 16792 | 1.930599 | 1 | 0.794293 | 66.00357 |
| GOTERM_BP_DIRECT | GO:0042594~response to starvation | 5 | 0.004297 | 0.059626 | UCN3, GIP, SSTR3, SSTR1, ADCY | 714 | 35 | 16792 | 3.359744 | 1 | 0.792773 | 66.2086 |
| GOTERM_BP_DIRECT | GO:0040018~positive regulation of multicellular | 5 | 0.004297 | 0.059626 | GHRH, DRD2, SPTBN4, ATP8A2, | 714 | 35 | 16792 | 3.359744 | 1 | 0.792773 | 66.2086 |
| GOTERM_BP_DIRECT | GO:0086091~regulation of heart rate by cardiac | 5 | 0.004297 | 0.059626 | KCNJ5, ANK2, SCN2B, SCN3B, SC | 714 | 35 | 16792 | 3.359744 | 1 | 0.792773 | 66.2086 |
| GOTERM_BP_DIRECT | GO:0007157~heterophilic cell-cell adhesion via p | 6 | 0.005156 | 0.060019 | ALCAM, CADM4, CADM1, CADM | 714 | 50 | 16792 | 2.822185 | 1 | 0.791674 | 66.45735 |
| GOTERM_BP_DIRECT | GO:0042493~response to drug | 20 | 0.017188 | 0.060971 | RET, GIP, DRD2, SLC6A2, MAOB, | 714 | 304 | 16792 | 1.54725 | 1 | 0.79369 | 67.05155 |
| GOTERM_BP_DIRECT | GO:0031175~neuron projection development | 9 | 0.007735 | 0.062366 | FLRT3, NPY, EPHA8, MAP2, NLG | 714 | 100 | 16792 | 2.116639 | 1 | 0.798042 | 67.90518 |
| GOTERM_BP_DIRECT | GO:0050796~regulation of insulin secretion | 7 | 0.006016 | 0.064481 | GIP, KCNB1, TFAP2B, SYT9, NOS. | 714 | 67 | 16792 | 2.457126 | 1 | 0.805933 | 69.15895 |
| GOTERM_BP_DIRECT | GO:0061564~axon development | 3 | 0.002578 | 0.064623 | NEFL, NEFM, GAP43 | 714 | 10 | 16792 | 7.055462 | 1 | 0.803548 | 69.24158 |
| GOTERM_BP_DIRECT | GO:0060322~head development | 3 | 0.002578 | 0.064623 | FLRT3, LHX1, STRA6 | 714 | 10 | 16792 | 7.055462 | 1 | 0.803548 | 69.24158 |
| GOTERM_BP_DIRECT | GO:0048387~negative regulation of retinoic acic | 3 | 0.002578 | 0.064623 | ZNF536, CYP26C1, CYP26A1 | 714 | 10 | 16792 | 7.055462 | 1 | 0.803548 | 69.24158 |
| GOTERM_BP_DIRECT | GO:0010996~response to auditory stimulus | 3 | 0.002578 | 0.064623 | KCNC1, SLC26A5, ATP8A2 | 714 | 10 | 16792 | 7.055462 | 1 | 0.803548 | 69.24158 |
| GOTERM_BP_DIRECT | GO:0007611~learning or memory | 5 | 0.004297 | 0.064924 | S100B, KCNAB1, NTRK1, SHC3, ( | 714 | 36 | 16792 | 3.266418 | 1 | 0.80199 | 69.41539 |
| GOTERM_BP_DIRECT | GO:0006810~transport | 22 | 0.018907 | 0.06758 | GABRG2, GRIK1, SLC9A5, SLC6A | 714 | 348 | 16792 | 1.486783 | 1 | 0.812127 | 70.91299 |
| GOTERM_BP_DIRECT | GO:0035094~response to nicotine | 5 | 0.004297 | 0.070459 | PENK, DRD2, CHRNB4, CHRNA7, | 714 | 37 | 16792 | 3.178136 | 1 | 0.822611 | 72.45777 |
| GOTERM_BP_DIRECT | GO:0048813~dendrite morphogenesis | 5 | 0.004297 | 0.070459 | MAP2, ELAVL4, DCX, DCLK1, CA( | 714 | 37 | 16792 | 3.178136 | 1 | 0.822611 | 72.45777 |
| GOTERM_BP_DIRECT | GO:0043278~response to morphine | 4 | 0.003438 | 0.071863 | PENK, DRD2, TAC1, PRKCE | 714 | 23 | 16792 | 4.090123 | 1 | 0.826 | 73.18303 |
| GOTERM_BP_DIRECT | GO:0050770~regulation of axonogenesis | 4 | 0.003438 | 0.071863 | RET, POU3F2, LRRC4C, CACNA1 | 714 | 23 | 16792 | 4.090123 | 1 | 0.826 | 73.18303 |
| GOTERM_BP_DIRECT | GO:0019933~cAMP-mediated signaling | 5 | 0.004297 | 0.076227 | GHRH, AKAP6, GAL, GLP1R, ADC | 714 | 38 | 16792 | 3.094501 | 1 | 0.8415 | 75.32333 |
| GOTERM_BP_DIRECT | GO:0007154~cell communication | 5 | 0.004297 | 0.076227 | GJD2, ENPP5, SLC8A2, SNTG1, N | 714 | 38 | 16792 | 3.094501 | 1 | 0.8415 | 75.32333 |
| GOTERM_BP_DIRECT | GO:0042551~neuron maturation | 3 | 0.002578 | 0.076819 | RET, VSX1, FEV | 714 | 11 | 16792 | 6.414057 | 1 | 0.841161 | 75.60109 |
| GOTERM_BP_DIRECT | GO:1901652~response to peptide | 3 | 0.002578 | 0.076819 | HCRTR1, GPR22, NPFFR2 | 714 | 11 | 16792 | 6.414057 | 1 | 0.841161 | 75.60109 |
| GOTERM_BP_DIRECT | GO:0048266~behavioral response to pain | 3 | 0.002578 | 0.076819 | PIRT, SCN9A, CACNA1A | 714 | 11 | 16792 | 6.414057 | 1 | 0.841161 | 75.60109 |
| GOTERM_BP_DIRECT | GO:0002064~epithelial cell development | 3 | 0.002578 | 0.076819 | SHROOM3, HYDIN, ONECUT2 | 714 | 11 | 16792 | 6.414057 | 1 | 0.841161 | 75.60109 |
| GOTERM_BP_DIRECT | GO:0048484~enteric nervous system developme | 3 | 0.002578 | 0.076819 | RET, GDNF, TLX2 | 714 | 11 | 16792 | 6.414057 | 1 | 0.841161 | 75.60109 |
| GOTERM_BP_DIRECT | GO:1900273~positive regulation of long-term sy | 3 | 0.002578 | 0.076819 | DRD2, RELN, NRGN | 714 | 11 | 16792 | 6.414057 | 1 | 0.841161 | 75.60109 |
| GOTERM_BP_DIRECT | GO:0030317~sperm motility | 6 | 0.005156 | 0.078264 | LRRC6, APOB, DNAI1, CHRNA7, ( | 714 | 54 | 16792 | 2.613134 | 1 | 0.84415 | 76.26616 |
| GOTERM_BP_DIRECT | GO:0048015~phosphatidylinositol-mediated sig | 9 | 0.007735 | 0.08153 | KCNH1, IGF1R, PIRT, NTRK1, FGF | 714 | 106 | 16792 | 1.996829 | 1 | 0.853736 | 77.70727 |
| GOTERM_BP_DIRECT | GO:0014016~neuroblast differentiation | 2 | 0.001719 | 0.083121 | BCHE, SIX3 | 714 | 2 | 16792 | 23.51821 | 1 | 0.856872 | 78.37908 |
| GOTERM_BP_DIRECT | GO:0010949~negative regulation of intestinal pl | 2 | 0.001719 | 0.083121 | ABCG8, ABCG5 | 714 | 2 | 16792 | 23.51821 | 1 | 0.856872 | 78.37908 |
| GOTERM_BP_DIRECT | GO:0042418~epinephrine biosynthetic process | 2 | 0.001719 | 0.083121 | PNMT, TH | 714 | 2 | 16792 | 23.51821 | 1 | 0.856872 | 78.37908 |
| GOTERM_BP_DIRECT | GO:0031645~negative regulation of neurologica | 2 | 0.001719 | 0.083121 | CALCA, NPY2R | 714 | 2 | 16792 | 23.51821 | 1 | 0.856872 | 78.37908 |
| GOTERM_BP_DIRECT | GO:1905069~allantois development | 2 | 0.001719 | 0.083121 | BMP7, BMP5 | 714 | 2 | 16792 | 23.51821 | 1 | 0.856872 | 78.37908 |
| GOTERM_BP_DIRECT | GO:0001988~positive regulation of heart rate in | 2 | 0.001719 | 0.083121 | CHRNA7, CHRFAM7A | 714 | 2 | 16792 | 23.51821 | 1 | 0.856872 | 78.37908 |
| GOTERM_BP_DIRECT | GO:0060700~regulation of ribonuclease activity | 2 | 0.001719 | 0.083121 | OAS3, OAS1 | 714 | 2 | 16792 | 23.51821 | 1 | 0.856872 | 78.37908 |
| GOTERM_BP_DIRECT | GO:0045796~negative regulation of intestinal ch | 2 | 0.001719 | 0.083121 | ABCG8, ABCG5 | 714 | 2 | 16792 | 23.51821 | 1 | 0.856872 | 78.37908 |
| GOTERM_BP_DIRECT | GO:0071379~cellular response to prostaglandin | 2 | 0.001719 | 0.083121 | AKR1C3, APOB | 714 | 2 | 16792 | 23.51821 | 1 | 0.856872 | 78.37908 |
| GOTERM_BP_DIRECT | GO:0071469~cellular response to alkaline pH | 2 | 0.001719 | 0.083121 | KCNK4, INSRR | 714 | 2 | 16792 | 23.51821 | 1 | 0.856872 | 78.37908 |
| GOTERM_BP_DIRECT | GO:0018105~peptidyl-serine phosphorylation | 10 | 0.008594 | 0.084731 | CAMK4, TTBK2, TTBK1, MAPK13 | 714 | 125 | 16792 | 1.881457 | 1 | 0.85996 | 79.03925 |
| GOTERM_BP_DIRECT | GO:0051899~membrane depolarization | 4 | 0.003438 | 0.087772 | SCN3B, SCN5A, CACNA1A, CACN | 714 | 25 | 16792 | 3.762913 | 1 | 0.867589 | 80.23508 |
| GOTERM_BP_DIRECT | GO:0001578~microtubule bundle formation | 4 | 0.003438 | 0.087772 | CAPN6, NAV1, TPPP, MAP2 | 714 | 25 | 16792 | 3.762913 | 1 | 0.867589 | 80.23508 |
| GOTERM_BP_DIRECT | GO:0021766~hippocampus development | 6 | 0.005156 | 0.088383 | SLC32A1, DLX2, BCAN, RELN, DC | 714 | 56 | 16792 | 2.519808 | 1 | 0.867194 | 80.46724 |
| GOTERM_BP_DIRECT | GO:0045665~negative regulation of neuron diffe | 6 | 0.005156 | 0.088383 | ZNF536, ISL2, DTX1, CNTN2, SIX | 714 | 56 | 16792 | 2.519808 | 1 | 0.867194 | 80.46724 |
| GOTERM_BP_DIRECT | GO:0048468~cell development | 5 | 0.004297 | 0.088448 | DCT, GDF1, IRF6, GDF10, GDF15 | 714 | 40 | 16792 | 2.939776 | 1 | 0.865072 | 80.49192 |
| GOTERM_BP_DIRECT | GO:0007190~activation of adenylate cyclase act | 5 | 0.004297 | 0.088448 | CALCA, DRD5, LHCGR, GLP1R, AI | 714 | 40 | 16792 | 2.939776 | 1 | 0.865072 | 80.49192 |
| GOTERM_BP_DIRECT | GO:0035641~locomotory exploration behavior | 3 | 0.002578 | 0.089667 | PENK, TNR, PRKCE | 714 | 12 | 16792 | 5.879552 | 1 | 0.866625 | 80.94723 |
| GOTERM_BP_DIRECT | GO:0002031~G-protein coupled receptor interna | 3 | 0.002578 | 0.089667 | CALCA, DRD2, DNM1 | 714 | 12 | 16792 | 5.879552 | 1 | 0.866625 | 80.94723 |
| GOTERM_BP_DIRECT | GO:0043950~positive regulation of cAMP-media | 3 | 0.002578 | 0.089667 | OPRM1, GIP, LHCGR | 714 | 12 | 16792 | 5.879552 | 1 | 0.866625 | 80.94723 |
| GOTERM_BP_DIRECT | GO:0055008~cardiac muscle tissue morphogene | 3 | 0.002578 | 0.089667 | TCAP, TBX20, ZFPM1 | 714 | 12 | 16792 | 5.879552 | 1 | 0.866625 | 80.94723 |
| GOTERM_BP_DIRECT | GO:0032230~positive regulation of synaptic trar | 3 | 0.002578 | 0.089667 | NLGN1, TAC1, PRKCE | 714 | 12 | 16792 | 5.879552 | 1 | 0.866625 | 80.94723 |
| GOTERM_BP_DIRECT | GO:0043401~steroid hormone mediated signalir | 6 | 0.005156 | 0.093685 | NR1I2, NR6A1, ESRRG, PAQR6, F | 714 | 57 | 16792 | 2.475601 | 1 | 0.876507 | 82.37802 |
| GOTERM_BP_DIRECT | GO:0000165~MAPK cascade | 17 | 0.01461 | 0.094229 | RET, SPTBN4, FGF16, RASGEF1A | 714 | 262 | 16792 | 1.525991 | 1 | 0.875899 | 82.56382 |
| GOTERM_BP_DIRECT | GO:0032024~positive regulation of insulin secre | 5 | 0.004297 | 0.09489 | UCN3, MYRIP, GIP, NNAT, PRKCE | 714 | 41 | 16792 | 2.868074 | 1 | 0.875636 | 82.78721 |
| GOTERM_BP_DIRECT | GO:0043408~regulation of MAPK cascade | 5 | 0.004297 | 0.09489 | GDF1, NPFFR2, GDF10, GDF15, I | 714 | 41 | 16792 | 2.868074 | 1 | 0.875636 | 82.78721 |
| GOTERM_BP_DIRECT | GO:0000278~mitotic cell cycle | 5 | 0.004297 | 0.09489 | XRCC2, PAK3, PRDM5, PPP2R2B, | 714 | 41 | 16792 | 2.868074 | 1 | 0.875636 | 82.78721 |
| GOTERM_BP_DIRECT | GO:0009887~organ morphogenesis | 8 | 0.006875 | 0.096042 | ETV7, LHX1, COMP, FGF16, TH, ( | 714 | 92 | 16792 | 2.045062 | 1 | 0.876771 | 83.1695 |
| GOTERM_BP_DIRECT | GO:0030534~adult behavior | 4 | 0.003438 | 0.096233 | GABRG2, NRXN3, PAX5, SHANK2 | 714 | 26 | 16792 | 3.618186 | 1 | 0.875183 | 83.23232 |
| GOTERM_BP_DIRECT | GO:0072661~protein targeting to plasma memb | 4 | 0.003438 | 0.096233 | ANK1, ANK2, KCNB2, KCNB1 | 714 | 26 | 16792 | 3.618186 | 1 | 0.875183 | 83.23232 |
| GOTERM_BP_DIRECT | GO:0017157~regulation of exocytosis | 4 | 0.003438 | 0.096233 | RAB3C, RAB26, STX1B, STXBP5L | 714 | 26 | 16792 | 3.618186 | 1 | 0.875183 | 83.23232 |

**Enriched GO BP terms by genes over-expressed in Subtype II PCC**

| Category | Term | Count | % | PValue | Genes | List Total | Pop Hits | Pop Total | Fold Enric | Bonferron | Benjamin | FDR |
|---|---|---|---|---|---|---|---|---|---|---|---|---|
| GOTERM_BP_DIRECT | GO:0007267~cell-cell signaling | 40 | 4.405286 | 5.09E-11 | ADORA2A, FGF9, EFNA1, CTF1, I | 786 | 254 | 16792 | 3.364389 | 1.59E-07 | 1.59E-07 | 9.21E-08 |
| GOTERM_BP_DIRECT | GO:0060021~palate development | 16 | 1.762115 | 1.92E-06 | SATB2, PRRX1, LEF1, COL2A1, SI | 786 | 76 | 16792 | 4.497656 | 0.005977 | 0.002993 | 0.003478 |
| GOTERM_BP_DIRECT | GO:0008584~male gonad development | 17 | 1.872247 | 6.72E-06 | AMHR2, TESC, FGF9, ESR1, INHA | 786 | 94 | 16792 | 3.863678 | 0.020735 | 0.00696 | 0.012155 |
| GOTERM_BP_DIRECT | GO:0045926~negative regulation of growth | 8 | 0.881057 | 1.46E-05 | MT1L, MT1M, GPC3, MT1A, MT. | 786 | 19 | 16792 | 8.995313 | 0.044512 | 0.011319 | 0.026413 |
| GOTERM_BP_DIRECT | GO:0071805~potassium ion transmembrane tra | 18 | 1.982379 | 4.71E-05 | HPN, TRPM5, SLC9A2, KCNA5, K | 786 | 121 | 16792 | 3.178096 | 0.136569 | 0.028941 | 0.085154 |
| GOTERM_BP_DIRECT | GO:0070374~positive regulation of ERK1 and ER | 22 | 2.422907 | 7.02E-05 | NOX4, EGFR, BMP4, TNFAIP8L3, | 786 | 175 | 16792 | 2.685743 | 0.196627 | 0.035832 | 0.126937 |
| GOTERM_BP_DIRECT | GO:0071300~cellular response to retinoic acid | 13 | 1.431718 | 8.75E-05 | MUC1, ALDH1A2, TESC, FZD10, | 786 | 70 | 16792 | 3.967575 | 0.238816 | 0.038233 | 0.158188 |
| GOTERM_BP_DIRECT | GO:0001649~osteoblast differentiation | 16 | 1.762115 | 9.57E-05 | ALPL, BMP4, BMP2, FGF9, FHL2, | 786 | 104 | 16792 | 3.286749 | 0.257899 | 0.036597 | 0.172893 |
| GOTERM_BP_DIRECT | GO:0060070~canonical Wnt signaling pathway | 14 | 1.54185 | 1.18E-04 | FZD9, FZD8, NDP, LEF1, CDH3, S | 786 | 83 | 16792 | 3.603544 | 0.307389 | 0.039988 | 0.212857 |
| GOTERM_BP_DIRECT | GO:0071294~cellular response to zinc ion | 7 | 0.770925 | 1.64E-04 | MT1L, MT1M, MT1A, MT2A, MT | 786 | 19 | 16792 | 7.870899 | 0.401173 | 0.049986 | 0.297051 |
| GOTERM_BP_DIRECT | GO:0055085~transmembrane transport | 26 | 2.863436 | 2.00E-04 | MFSD7, SLC45A3, ABCA8, SLC13 | 786 | 244 | 16792 | 2.276478 | 0.463374 | 0.055015 | 0.360469 |
| GOTERM_BP_DIRECT | GO:0045165~cell fate commitment | 10 | 1.101322 | 2.36E-04 | WNT4, BMP2, GATA5, GATA6, G | 786 | 46 | 16792 | 4.644319 | 0.521072 | 0.059506 | 0.426203 |
| GOTERM_BP_DIRECT | GO:0010628~positive regulation of gene expres | 27 | 2.973568 | 2.51E-04 | FGFR4, FGF9, GJA1, PAWR, CDH. | 786 | 262 | 16792 | 2.20162 | 0.542193 | 0.05833 | 0.452255 |
| GOTERM_BP_DIRECT | GO:0000122~negative regulation of transcriptio | 55 | 6.057269 | 4.10E-04 | THRB, FGF9, EFNA1, FST, PRRX1, | 786 | 720 | 16792 | 1.631962 | 0.721457 | 0.087255 | 0.738803 |
| GOTERM_BP_DIRECT | GO:0045669~positive regulation of osteoblast d | 11 | 1.211454 | 4.25E-04 | MSX2, BMP4, WNT4, BMP2, IL6, | 786 | 60 | 16792 | 3.916709 | 0.733917 | 0.08448 | 0.765153 |

| | | | | | | | | | | | | |
|---|---|---|---|---|---|---|---|---|---|---|---|---|
| GOTERM_BP_DIRECT | GO:0008016~regulation of heart contraction | 8 | 0.881057 | 4.67E-04 | FXYD1, DES, THRB, PLN, HSPB7, | 786 | 31 | 16792 | 5.513256 | 0.76653 | 0.086908 | 0.840401 |
| GOTERM_BP_DIRECT | GO:0030513~positive regulation of BMP signalin | 8 | 0.881057 | 4.67E-04 | MSX2, BMP4, MSX1, GPC3, GAT/ | 786 | 31 | 16792 | 5.513256 | 0.76653 | 0.086908 | 0.840401 |
| GOTERM_BP_DIRECT | GO:0050729~positive regulation of inflammator | 12 | 1.321586 | 5.46E-04 | EGFR, LPL, CCL13, CCL23, CCL14, | 786 | 73 | 16792 | 3.511869 | 0.817648 | 0.095259 | 0.982461 |
| GOTERM_BP_DIRECT | GO:0051480~regulation of cytosolic calcium ion | 8 | 0.881057 | 5.73E-04 | FZD9, SCGN, TRPC4, PLN, RYR2, | 786 | 32 | 16792 | 5.340967 | 0.832676 | 0.09455 | 1.031856 |
| GOTERM_BP_DIRECT | GO:0035115~embryonic forelimb morphogenes | 8 | 0.881057 | 5.73E-04 | MSX2, ALDH1A2, MSX1, OSR2, C | 786 | 32 | 16792 | 5.340967 | 0.832676 | 0.09455 | 1.031856 |
| GOTERM_BP_DIRECT | GO:0009954~proximal/distal pattern formation | 7 | 0.770925 | 6.68E-04 | ALDH1A2, HOXA10, HOXA9, DLL | 786 | 24 | 16792 | 6.231128 | 0.875398 | 0.103818 | 1.200979 |
| GOTERM_BP_DIRECT | GO:0042475~odontogenesis of dentin-containin | 10 | 1.101322 | 9.36E-04 | DLX3, BMP4, SMO, BMP2, MSX1 | 786 | 55 | 16792 | 3.88434 | 0.946081 | 0.135854 | 1.679944 |
| GOTERM_BP_DIRECT | GO:0001666~response to hypoxia | 19 | 2.092511 | 0.001174 | MUC1, NOX4, BMP2, EGLN3, ITC | 786 | 172 | 16792 | 2.359962 | 0.97434 | 0.160056 | 2.102593 |
| GOTERM_BP_DIRECT | GO:0055078~sodium ion homeostasis | 5 | 0.550661 | 0.001201 | CYP4A11, CYP11B2, CYP4F12, SC | 786 | 11 | 16792 | 9.710849 | 0.976409 | 0.156599 | 2.150312 |
| GOTERM_BP_DIRECT | GO:0043401~steroid hormone mediated signalir | 10 | 1.101322 | 0.001219 | PGR, BMP4, VDR, THRB, ESR1, LI | 786 | 57 | 16792 | 3.748047 | 0.97769 | 0.152391 | 2.182021 |
| GOTERM_BP_DIRECT | GO:1902476~chloride transmembrane transpor | 13 | 1.431718 | 0.001286 | FXYD1, GABRD, GABRE, SLC12A1 | 786 | 93 | 16792 | 2.986347 | 0.981885 | 0.153906 | 2.300137 |
| GOTERM_BP_DIRECT | GO:0006813~potassium ion transport | 12 | 1.321586 | 0.001468 | KCNJ15, KCNJ4, KCND3, KCNS1, | 786 | 82 | 16792 | 3.12642 | 0.989749 | 0.16741 | 2.622311 |
| GOTERM_BP_DIRECT | GO:0035116~embryonic hindlimb morphogenes | 7 | 0.770925 | 0.001594 | MSX2, BMP4, MSX1, OSR2, GPC3 | 786 | 28 | 16792 | 5.340967 | 0.993071 | 0.174061 | 2.843385 |
| GOTERM_BP_DIRECT | GO:0050679~positive regulation of epithelial cel | 10 | 1.101322 | 0.001769 | BMP4, EGFR, SMO, CYP7B1, IL6, | 786 | 60 | 16792 | 3.560645 | 0.995988 | 0.184854 | 3.150864 |
| GOTERM_BP_DIRECT | GO:0001501~skeletal system development | 16 | 1.762115 | 0.001875 | ALPL, BMP2, BMP1, HAPLN3, SO | 786 | 137 | 16792 | 2.49505 | 0.997121 | 0.188552 | 3.33707 |
| GOTERM_BP_DIRECT | GO:0006810~transport | 30 | 3.303965 | 0.001934 | ABCA8, FXYD2, GABRB2, CLCNK/ | 786 | 348 | 16792 | 1.841713 | 0.997603 | 0.187836 | 3.439841 |
| GOTERM_BP_DIRECT | GO:0002062~chondrocyte differentiation | 8 | 0.881057 | 0.001972 | NOV, BMP4, BMP2, OSR2, OSR1 | 786 | 39 | 16792 | 4.382332 | 0.997874 | 0.185453 | 3.507127 |
| GOTERM_BP_DIRECT | GO:0007409~axonogenesis | 13 | 1.431718 | 0.002029 | SLITRK2, SLITRK1, OMD, SLITRK4 | 786 | 98 | 16792 | 2.833982 | 0.998217 | 0.18468 | 3.60544 |
| GOTERM_BP_DIRECT | GO:0048468~cell development | 8 | 0.881057 | 0.002296 | INHBB, BMP1, GATA5, GATA6, G | 786 | 40 | 16792 | 4.272774 | 0.999226 | 0.200597 | 4.071312 |
| GOTERM_BP_DIRECT | GO:0045987~positive regulation of smooth mus | 6 | 0.660793 | 0.002399 | MYOCD, NPNT, ADRA1B, TBXA2I | 786 | 21 | 16792 | 6.103962 | 0.99944 | 0.203001 | 4.251068 |
| GOTERM_BP_DIRECT | GO:0014032~neural crest cell development | 5 | 0.550661 | 0.002415 | EDNRA, ALDH1A2, SOX11, FOXC | 786 | 13 | 16792 | 8.216872 | 0.999467 | 0.19882 | 4.278289 |
| GOTERM_BP_DIRECT | GO:0007165~signal transduction | 76 | 8.370044 | 0.002636 | GNA14, FGF9, CRHBP, IGFBP6, F | 786 | 1161 | 16792 | 1.398496 | 0.999733 | 0.209473 | 4.660785 |
| GOTERM_BP_DIRECT | GO:0030178~negative regulation of Wnt signalir | 9 | 0.991189 | 0.002669 | MDFI, BARX1, DACT1, FGF9, WIF | 786 | 52 | 16792 | 3.697592 | 0.999759 | 0.206601 | 4.718577 |
| GOTERM_BP_DIRECT | GO:0030509~BMP signaling pathway | 11 | 1.211454 | 0.002749 | BMP4, RGMA, BMP2, BMP1, GD | 786 | 76 | 16792 | 3.092139 | 0.999812 | 0.207003 | 4.856779 |
| GOTERM_BP_DIRECT | GO:0006869~lipid transport | 11 | 1.211454 | 0.002749 | ABCA8, SPNS2, SPNS3, APOD, SE | 786 | 76 | 16792 | 3.092139 | 0.999812 | 0.207003 | 4.856779 |
| GOTERM_BP_DIRECT | GO:0071392~cellular response to estradiol stim | 7 | 0.770925 | 0.002765 | MSX2, EGFR, IL6, ZNF703, CRHBI | 786 | 31 | 16792 | 4.824099 | 0.999821 | 0.203165 | 4.883528 |
| GOTERM_BP_DIRECT | GO:0001658~branching involved in ureteric bud | 8 | 0.881057 | 0.003064 | BMP4, TCF21, WNT4, BMP2, GP | 786 | 42 | 16792 | 4.069308 | 0.99993 | 0.217523 | 5.398754 |
| GOTERM_BP_DIRECT | GO:0060065~uterus development | 5 | 0.550661 | 0.003257 | FOXL2, MYOCD, HOXA10, ESR1, | 786 | 14 | 16792 | 7.629953 | 0.999962 | 0.224479 | 5.728838 |
| GOTERM_BP_DIRECT | GO:0045907~positive regulation of vasoconstric | 7 | 0.770925 | 0.003271 | EGFR, FGG, AVPR1A, ADRA1B, TI | 786 | 32 | 16792 | 4.673346 | 0.999963 | 0.220462 | 5.752039 |
| GOTERM_BP_DIRECT | GO:0007605~sensory perception of sound | 15 | 1.651982 | 0.00376 | SLC26A4, COL4A3, CLRN1, THRB | 786 | 133 | 16792 | 2.409459 | 0.999992 | 0.243885 | 6.58513 |
| GOTERM_BP_DIRECT | GO:0009952~anterior/posterior pattern specific | 11 | 1.211454 | 0.004014 | MSX2, SMO, ALDH1A2, BARX1, I | 786 | 80 | 16792 | 2.937532 | 0.999996 | 0.252891 | 7.014888 |
| GOTERM_BP_DIRECT | GO:0008284~positive regulation of cell prolifera | 36 | 3.964758 | 0.004014 | CLDN7, FGFR4, FGF9, CTF1, PTH | 786 | 466 | 16792 | 1.650428 | 0.999996 | 0.247946 | 7.015569 |
| GOTERM_BP_DIRECT | GO:0006936~muscle contraction | 13 | 1.431718 | 0.004231 | FXYD1, ACTG2, DES, CKMT2, SN | 786 | 107 | 16792 | 2.59561 | 0.999998 | 0.254477 | 7.380298 |
| GOTERM_BP_DIRECT | GO:0045880~positive regulation of smoothened | 6 | 0.660793 | 0.004463 | SMO, GPC3, FGF9, PRRX1, WNT | 786 | 24 | 16792 | 5.340967 | 0.999999 | 0.261446 | 7.769428 |
| GOTERM_BP_DIRECT | GO:0001755~neural crest cell migration | 8 | 0.881057 | 0.004567 | SMO, EDNRB, NRTN, LEF1, SEM/ | 786 | 45 | 16792 | 3.798021 | 0.999999 | 0.261839 | 7.944808 |
| GOTERM_BP_DIRECT | GO:0030182~neuron differentiation | 12 | 1.321586 | 0.00474 | FZD8, ALDH1A2, WNT4, PTPRD, | 786 | 95 | 16792 | 2.698594 | 1 | 0.265489 | 8.233553 |
| GOTERM_BP_DIRECT | GO:0030198~extracellular matrix organization | 19 | 2.092511 | 0.004901 | COL4A4, COL4A3, COL13A1, EGI | 786 | 196 | 16792 | 2.070987 | 1 | 0.268396 | 8.500869 |
| GOTERM_BP_DIRECT | GO:0030855~epithelial cell differentiation | 10 | 1.101322 | 0.005146 | MUC1, PTER, TST, TCF21, BARX1 | 786 | 70 | 16792 | 3.051981 | 1 | 0.275029 | 8.907607 |
| GOTERM_BP_DIRECT | GO:0030501~positive regulation of bone minera | 7 | 0.770925 | 0.005202 | FZD9, BMP4, WNT4, BMP2, OSR | 786 | 35 | 16792 | 4.272774 | 1 | 0.272971 | 9.001128 |
| GOTERM_BP_DIRECT | GO:0048566~embryonic digestive tract develop | 5 | 0.550661 | 0.005497 | ALDH1A2, RARRES2, FGF9, GLI3, | 786 | 16 | 16792 | 6.676209 | 1 | 0.281354 | 9.486502 |
| GOTERM_BP_DIRECT | GO:0007155~cell adhesion | 35 | 3.854626 | 0.005568 | OPCML, MYBPC3, ITGA10, MMR | 786 | 459 | 16792 | 1.629053 | 1 | 0.279918 | 9.60437 |
| GOTERM_BP_DIRECT | GO:0034765~regulation of ion transmembrane t | 13 | 1.431718 | 0.005686 | KCNJ15, KCNJ4, TMEM37, KCND | 786 | 111 | 16792 | 2.502075 | 1 | 0.280469 | 9.798053 |
| GOTERM_BP_DIRECT | GO:0010043~response to zinc ion | 7 | 0.770925 | 0.006001 | ASS1, PLN, KRT14, HAAO, SLC30 | 786 | 36 | 16792 | 4.154085 | 1 | 0.28903 | 10.31334 |
| GOTERM_BP_DIRECT | GO:2000721~positive regulation of transcriptior | 3 | 0.330396 | 0.006345 | MYOCD, ITGA8, NPNT | 786 | 3 | 16792 | 21.36387 | 1 | 0.298319 | 10.87227 |
| GOTERM_BP_DIRECT | GO:0072138~mesenchymal cell proliferation inv | 3 | 0.330396 | 0.006345 | BMP4, BMP2, GPC3 | 786 | 3 | 16792 | 21.36387 | 1 | 0.298319 | 10.87227 |
| GOTERM_BP_DIRECT | GO:0070328~triglyceride homeostasis | 6 | 0.660793 | 0.006394 | LPL, GCKR, SCARB1, CETP, GPIHE | 786 | 26 | 16792 | 4.930123 | 1 | 0.295848 | 10.95182 |
| GOTERM_BP_DIRECT | GO:0002053~positive regulation of mesenchym | 6 | 0.660793 | 0.006394 | SMO, FGF9, PRRX1, TBX1, CHRD | 786 | 26 | 16792 | 4.930123 | 1 | 0.295848 | 10.95182 |
| GOTERM_BP_DIRECT | GO:0006811~ion transport | 14 | 1.54185 | 0.006483 | SLC12A1, GABRB2, SLCO4A1, SL | 786 | 127 | 16792 | 2.355072 | 1 | 0.295 | 11.09686 |
| GOTERM_BP_DIRECT | GO:0006954~inflammatory response | 30 | 3.303965 | 0.006486 | RARRES2, ADORA2A, CRHBP, GS | 786 | 379 | 16792 | 1.691071 | 1 | 0.290922 | 11.10163 |
| GOTERM_BP_DIRECT | GO:0010862~positive regulation of pathway-res | 8 | 0.881057 | 0.006568 | INHBB, BMP4, BMP2, RBPMS, BI | 786 | 48 | 16792 | 3.560645 | 1 | 0.289903 | 11.23439 |
| GOTERM_BP_DIRECT | GO:0001822~kidney development | 11 | 1.211454 | 0.006726 | BMP4, TCF21, ALDH1A2, WNT4, | 786 | 86 | 16792 | 2.732588 | 1 | 0.291668 | 11.48852 |
| GOTERM_BP_DIRECT | GO:0007494~midgut development | 4 | 0.440529 | 0.006917 | EGFR, SMO, ALDH1A2, ASS1 | 786 | 9 | 16792 | 9.495052 | 1 | 0.294567 | 11.79565 |
| GOTERM_BP_DIRECT | GO:0060539~diaphragm development | 4 | 0.440529 | 0.006917 | TCF21, ASS1, FGFRL1, MSC | 786 | 9 | 16792 | 9.495052 | 1 | 0.294567 | 11.79565 |
| GOTERM_BP_DIRECT | GO:0006704~glucocorticoid biosynthetic proces | 4 | 0.440529 | 0.006917 | HSD3B2, CYP21A2, HSD11B1, HS | 786 | 9 | 16792 | 9.495052 | 1 | 0.294567 | 11.79565 |
| GOTERM_BP_DIRECT | GO:0021542~dentate gyrus development | 5 | 0.550661 | 0.006926 | SMO, DRD1, EMX2, LEF1, NEUR( | 786 | 17 | 16792 | 6.28349 | 1 | 0.29096 | 11.80977 |
| GOTERM_BP_DIRECT | GO:0010460~positive regulation of heart rate | 5 | 0.550661 | 0.006926 | AVPR1A, RYR2, KCNQ1, HRC, AD | 786 | 17 | 16792 | 6.28349 | 1 | 0.29096 | 11.80977 |
| GOTERM_BP_DIRECT | GO:0071276~cellular response to cadmium ion | 5 | 0.550661 | 0.006926 | MT1A, MT1E, MT1G, MT1F, MT: | 786 | 17 | 16792 | 6.28349 | 1 | 0.29096 | 11.80977 |
| GOTERM_BP_DIRECT | GO:0001657~ureteric bud development | 7 | 0.770925 | 0.007861 | BMP4, TCF21, OSR1, SMAD6, NF | 786 | 38 | 16792 | 3.935449 | 1 | 0.319107 | 13.29938 |
| GOTERM_BP_DIRECT | GO:0060395~SMAD protein signal transduction | 9 | 0.991189 | 0.007975 | INHBB, BMP4, AFP, BMP2, BMP: | 786 | 62 | 16792 | 3.101207 | 1 | 0.318841 | 13.47973 |
| GOTERM_BP_DIRECT | GO:0051965~positive regulation of synapse asse | 9 | 0.991189 | 0.007975 | SLITRK2, FLRT2, SLITRK1, SLITRK | 786 | 62 | 16792 | 3.101207 | 1 | 0.318841 | 13.47973 |
| GOTERM_BP_DIRECT | GO:0036150~phosphatidylserine acyl-chain rem | 5 | 0.550661 | 0.00858 | PLA2G4A, MBOAT1, PLA2G2A, P | 786 | 18 | 16792 | 5.934408 | 1 | 0.334328 | 14.42915 |
| GOTERM_BP_DIRECT | GO:0048557~embryonic digestive tract morpho | 5 | 0.550661 | 0.00858 | RBPMS2, TCF21, ID2, SOX11, GL | 786 | 18 | 16792 | 5.934408 | 1 | 0.334328 | 14.42915 |
| GOTERM_BP_DIRECT | GO:0021983~pituitary gland development | 6 | 0.660793 | 0.008846 | BMP4, ALDH1A2, MSX1, SOX3, I | 786 | 28 | 16792 | 4.577972 | 1 | 0.338571 | 14.84287 |
| GOTERM_BP_DIRECT | GO:0007160~cell-matrix adhesion | 11 | 1.211454 | 0.009205 | COL17A1, FGG, LYVE1, LYPD3, C | 786 | 90 | 16792 | 2.611139 | 1 | 0.345517 | 15.39993 |
| GOTERM_BP_DIRECT | GO:0045944~positive regulation of transcriptior | 63 | 6.938326 | 0.009337 | CDX1, THRB, EVX1, F2RL1, PRRX | 786 | 981 | 16792 | 1.371992 | 1 | 0.34544 | 15.60373 |
| GOTERM_BP_DIRECT | GO:0090090~negative regulation of canonical W | 16 | 1.762115 | 0.009524 | BMP2, NKD1, HECW1, IGFBP6, L | 786 | 163 | 16792 | 2.097067 | 1 | 0.346977 | 15.8913 |
| GOTERM_BP_DIRECT | GO:0046426~negative regulation of JAK-STAT ca | 7 | 0.770925 | 0.010102 | ASPN, FLRT2, RTN4RL1, RTN4R, | 786 | 40 | 16792 | 3.738677 | 1 | 0.35966 | 16.77411 |
| GOTERM_BP_DIRECT | GO:0071549~cellular response to dexamethasoı | 6 | 0.660793 | 0.010286 | EGFR, IL6, ASS1, SERPINF1, FBX( | 786 | 29 | 16792 | 4.420111 | 1 | 0.360847 | 17.05337 |
| GOTERM_BP_DIRECT | GO:0006508~proteolysis | 36 | 3.964758 | 0.011466 | AMZ1, ADAMTS17, MASP1, DPP | 786 | 500 | 16792 | 1.538198 | 1 | 0.388858 | 18.82391 |
| GOTERM_BP_DIRECT | GO:0090184~positive regulation of kidney devel | 3 | 0.330396 | 0.012296 | BMP4, SOX8, FOXD1 | 786 | 4 | 16792 | 16.0229 | 1 | 0.406167 | 20.04778 |
| GOTERM_BP_DIRECT | GO:0006705~mineralocorticoid biosynthetic prc | 3 | 0.330396 | 0.012296 | HSD3B2, CYP21A2, CYP11B2 | 786 | 4 | 16792 | 16.0229 | 1 | 0.406167 | 20.04778 |
| GOTERM_BP_DIRECT | GO:0072498~embryonic skeletal joint developm | 3 | 0.330396 | 0.012296 | OSR2, OSR1, WNT9A | 786 | 4 | 16792 | 16.0229 | 1 | 0.406167 | 20.04778 |
| GOTERM_BP_DIRECT | GO:0042474~middle ear morphogenesis | 5 | 0.550661 | 0.012616 | MSX1, OSR2, OSR1, PRRX1, TBX: | 786 | 20 | 16792 | 5.340967 | 1 | 0.410014 | 20.51423 |
| GOTERM_BP_DIRECT | GO:0007275~multicellular organism developme | 37 | 4.07489 | 0.012645 | CDX1, POU6F2, SPESP1, PRRX2, | 786 | 521 | 16792 | 1.517204 | 1 | 0.40661 | 20.5559 |
| GOTERM_BP_DIRECT | GO:0030857~negative regulation of epithelial ce | 4 | 0.440529 | 0.012668 | SMO, OSR1, DLL1, YAP1 | 786 | 11 | 16792 | 7.768679 | 1 | 0.403153 | 20.59031 |
| GOTERM_BP_DIRECT | GO:0060272~embryonic skeletal joint morphog | 4 | 0.440529 | 0.012668 | BMP4, OSR2, OSR1, COL2A1 | 786 | 11 | 16792 | 7.768679 | 1 | 0.403153 | 20.59031 |
| GOTERM_BP_DIRECT | GO:0048711~positive regulation of astrocyte dif | 4 | 0.440529 | 0.012668 | MAG, BMP2, ID2, CLCF1 | 786 | 11 | 16792 | 7.768679 | 1 | 0.403153 | 20.59031 |
| GOTERM_BP_DIRECT | GO:0045740~positive regulation of DNA replicat | 7 | 0.770925 | 0.012764 | EGFR, IL6, PLA2G1B, PDGFC, ARI | 786 | 42 | 16792 | 3.560645 | 1 | 0.40151 | 20.72916 |
| GOTERM_BP_DIRECT | GO:0006814~sodium ion transport | 10 | 1.101322 | 0.013138 | SLC38A4, SLC4A10, SLC13A5, SL | 786 | 81 | 16792 | 2.637515 | 1 | 0.406552 | 21.27077 |
| GOTERM_BP_DIRECT | GO:0071356~cellular response to tumor necrosi | 12 | 1.321586 | 0.013834 | CCL13, IL6, CCL23, CCL14, ASS1, | 786 | 110 | 16792 | 2.330604 | 1 | 0.418866 | 22.2689 |
| GOTERM_BP_DIRECT | GO:0016055~Wnt signaling pathway | 17 | 1.872247 | 0.014581 | FZD9, NKD1, NDP, LEF1, NOTUM | 786 | 187 | 16792 | 1.94217 | 1 | 0.431775 | 23.32694 |
| GOTERM_BP_DIRECT | GO:0003007~heart morphogenesis | 6 | 0.660793 | 0.015551 | FLRT2, ALDH1A2, MYBPC3, TGFI | 786 | 32 | 16792 | 4.005725 | 1 | 0.448863 | 24.68005 |
| GOTERM_BP_DIRECT | GO:0050873~brown fat cell differentiation | 6 | 0.660793 | 0.015551 | RARRES2, ADRB1, SLC2A4, MRA | 786 | 32 | 16792 | 4.005725 | 1 | 0.448863 | 24.68005 |
| GOTERM_BP_DIRECT | GO:0035815~positive regulation of renal sodiun | 4 | 0.440529 | 0.016312 | EDNRB, ADORA2A, AVPR1A, NPI | 786 | 12 | 16792 | 7.121289 | 1 | 0.460792 | 25.72668 |
| GOTERM_BP_DIRECT | GO:0003376~sphingosine-1-phosphate signaling | 4 | 0.440529 | 0.016312 | S1PR3, SPNS2, SPNS3, S1PR5 | 786 | 12 | 16792 | 7.121289 | 1 | 0.460792 | 25.72668 |
| GOTERM_BP_DIRECT | GO:0006469~negative regulation of protein kina | 11 | 1.211454 | 0.017285 | ASPN, FLRT2, TESC, IL6, RTN4RL: | 786 | 99 | 16792 | 2.373763 | 1 | 0.476392 | 27.04367 |
| GOTERM_BP_DIRECT | GO:0051260~protein homooligomerization | 16 | 1.762115 | 0.019089 | KCND3, SCUBE3, SCUBE1, GSDM | 786 | 177 | 16792 | 1.931197 | 1 | 0.506768 | 29.42779 |
| GOTERM_BP_DIRECT | GO:0007218~neuropeptide signaling pathway | 11 | 1.211454 | 0.019631 | GRP, QRFPR, NXPH4, GPR139, N | 786 | 101 | 16792 | 2.326758 | 1 | 0.512567 | 30.1301 |
| GOTERM_BP_DIRECT | GO:0034220~ion transmembrane transport | 18 | 1.982379 | 0.019815 | FXYD1, FXYD2, SLC12A1, GABRA | 786 | 210 | 16792 | 1.831189 | 1 | 0.511812 | 30.36637 |
| GOTERM_BP_DIRECT | GO:0042743~hydrogen peroxide metabolic proc | 3 | 0.330396 | 0.019862 | EGFR, DUOXA2, DUOXA1 | 786 | 5 | 16792 | 12.81832 | 1 | 0.508652 | 30.42666 |
| GOTERM_BP_DIRECT | GO:0072162~metanephric mesenchymal cell dif | 3 | 0.330396 | 0.019862 | TCF21, WNT4, OSR1 | 786 | 5 | 16792 | 12.81832 | 1 | 0.508652 | 30.42666 |
| GOTERM_BP_DIRECT | GO:0048143~astrocyte activation | 3 | 0.330396 | 0.019862 | EGFR, SMO, ADORA2A | 786 | 5 | 16792 | 12.81832 | 1 | 0.508652 | 30.42666 |
| GOTERM_BP_DIRECT | GO:0001837~epithelial to mesenchymal transitio | 6 | 0.660793 | 0.01991 | WNT4, BMP2, TGFBR3, LEF1, WI | 786 | 34 | 16792 | 3.770094 | 1 | 0.505568 | 30.48872 |
| GOTERM_BP_DIRECT | GO:0048286~lung alveolus development | 6 | 0.660793 | 0.01991 | BMP4, TCF21, MYOCD, HOPX, AI | 786 | 34 | 16792 | 3.770094 | 1 | 0.505568 | 30.48872 |
| GOTERM_BP_DIRECT | GO:0030282~bone mineralization | 6 | 0.660793 | 0.01991 | ASPN, BMP2, GPC3, CLEC3B, PTI | 786 | 34 | 16792 | 3.770094 | 1 | 0.505568 | 30.48872 |
| GOTERM_BP_DIRECT | GO:0061029~eyelid development in camera-typ | 4 | 0.440529 | 0.020482 | EGFR, OSR2, SOX11, TWIST1 | 786 | 13 | 16792 | 6.573498 | 1 | 0.511646 | 31.21783 |
| GOTERM_BP_DIRECT | GO:0003198~epithelial to mesenchymal transitio | 4 | 0.440529 | 0.020482 | MSX2, BMP4, MSX1, SNAI2 | 786 | 13 | 16792 | 6.573498 | 1 | 0.511646 | 31.21783 |
| GOTERM_BP_DIRECT | GO:0050730~regulation of peptidyl-tyrosine ph | 4 | 0.440529 | 0.020482 | EGFR, EFNA1, TMEM102, PDGF( | 786 | 13 | 16792 | 6.573498 | 1 | 0.511646 | 31.21783 |
| GOTERM_BP_DIRECT | GO:0003148~outflow tract septum morphogene | 5 | 0.550661 | 0.020646 | MSX2, BMP4, GATA6, SMAD6, T | 786 | 23 | 16792 | 4.644319 | 1 | 0.510602 | 31.42598 |
| GOTERM_BP_DIRECT | GO:0006654~phosphatidic acid biosynthetic pro | 6 | 0.660793 | 0.022362 | PLA2G4A, PLD1, PLA2G2A, PLA2 | 786 | 35 | 16792 | 3.662377 | 1 | 0.535235 | 33.56637 |
| GOTERM_BP_DIRECT | GO:0007568~aging | 15 | 1.651982 | 0.022817 | IL6, ASS1, PTH1R, DCN, NPY5R, I | 786 | 165 | 16792 | 1.94217 | 1 | 0.538654 | 34.12393 |
| GOTERM_BP_DIRECT | GO:0007517~muscle organ development | 10 | 1.101322 | 0.023093 | MSX1, SMTN, CTF1, ITGB1BP2, F | 786 | 89 | 16792 | 2.400435 | 1 | 0.539179 | 34.45971 |
| GOTERM_BP_DIRECT | GO:0036152~phosphatidylethanolamine acyl-ch | 5 | 0.550661 | 0.023883 | PLA2G4A, MBOAT1, PLA2G2A, P | 786 | 24 | 16792 | 4.450806 | 1 | 0.547563 | 35.41056 |

| | | | | | | | | | | | | |
|---|---|---|---|---|---|---|---|---|---|---|---|---|
| GOTERM_BP_DIRECT | GO:0051924~regulation of calcium ion transpor | 5 | 0.550661 | 0.023883 | SLN, P2RX1, ADORA2A, PLN, GJA | 786 | 24 | 16792 | 4.450806 | 1 | 0.547563 | 35.41056 |
| GOTERM_BP_DIRECT | GO:0072001~renal system development | 4 | 0.440529 | 0.025179 | BMP4, SMO, EMX2, TBX18 | 786 | 14 | 16792 | 6.103962 | 1 | 0.563082 | 36.94482 |
| GOTERM_BP_DIRECT | GO:0006816~calcium ion transport | 9 | 0.991189 | 0.025254 | VDR, TRPC4, SLN, CACHD1, NMU | 786 | 76 | 16792 | 2.529932 | 1 | 0.560415 | 37.03165 |
| GOTERM_BP_DIRECT | GO:0030335~positive regulation of cell migratio | 16 | 1.762115 | 0.026012 | BMP4, EGFR, BMP2, DRD1, FAM | 786 | 184 | 16792 | 1.857728 | 1 | 0.567548 | 37.91145 |
| GOTERM_BP_DIRECT | GO:0050727~regulation of inflammatory respon | 8 | 0.881057 | 0.027137 | IL1R1, DUOXA2, SLC7A2, DUOXA | 786 | 63 | 16792 | 2.712872 | 1 | 0.579464 | 39.1963 |
| GOTERM_BP_DIRECT | GO:0003333~amino acid transmembrane transp | 5 | 0.550661 | 0.027409 | SLC38A4, SLC6A9, SLC16A10, SL | 786 | 25 | 16792 | 4.272774 | 1 | 0.579485 | 39.50285 |
| GOTERM_BP_DIRECT | GO:0048709~oligodendrocyte differentiation | 5 | 0.550661 | 0.027409 | TRPC4, NTRK2, NLGN3, GLI3, SO | 786 | 25 | 16792 | 4.272774 | 1 | 0.579485 | 39.50285 |
| GOTERM_BP_DIRECT | GO:0042493~response to drug | 23 | 2.53304 | 0.028442 | LPL, IL6, DRD1, ASS1, ADORA2A, | 786 | 304 | 16792 | 1.616345 | 1 | 0.589537 | 40.65349 |
| GOTERM_BP_DIRECT | GO:0060363~cranial suture morphogenesis | 3 | 0.330396 | 0.028878 | MSX2, BMP4, TWIST1 | 786 | 6 | 16792 | 10.68193 | 1 | 0.591583 | 41.13361 |
| GOTERM_BP_DIRECT | GO:0061002~negative regulation of dendritic sp | 3 | 0.330396 | 0.028878 | NGEF, EFNA1, NLGN3 | 786 | 6 | 16792 | 10.68193 | 1 | 0.591583 | 41.13361 |
| GOTERM_BP_DIRECT | GO:0046881~positive regulation of follicle-stimu | 3 | 0.330396 | 0.028878 | INHBB, FOXL2, INHA | 786 | 6 | 16792 | 10.68193 | 1 | 0.591583 | 41.13361 |
| GOTERM_BP_DIRECT | GO:0050957~equilibrioception | 3 | 0.330396 | 0.028878 | CLRN1, USH1G, CDH23 | 786 | 6 | 16792 | 10.68193 | 1 | 0.591583 | 41.13361 |
| GOTERM_BP_DIRECT | GO:0003180~aortic valve morphogenesis | 3 | 0.330396 | 0.028878 | BMP4, EFNA1, TWIST1 | 786 | 6 | 16792 | 10.68193 | 1 | 0.591583 | 41.13361 |
| GOTERM_BP_DIRECT | GO:0061312~BMP signaling pathway involved in | 3 | 0.330396 | 0.028878 | MSX2, BMP4, MSX1 | 786 | 6 | 16792 | 10.68193 | 1 | 0.591583 | 41.13361 |
| GOTERM_BP_DIRECT | GO:0006198~cAMP catabolic process | 4 | 0.440529 | 0.030404 | PDE1B, PDE2A, PDE1A, PDE4C | 786 | 15 | 16792 | 5.697031 | 1 | 0.607171 | 42.78474 |
| GOTERM_BP_DIRECT | GO:0006691~leukotriene metabolic process | 4 | 0.440529 | 0.030404 | GGT5, CYP4A11, LTC4S, DPEP1 | 786 | 15 | 16792 | 5.697031 | 1 | 0.607171 | 42.78474 |
| GOTERM_BP_DIRECT | GO:0042060~wound healing | 9 | 0.991189 | 0.033067 | EGFR, IL6, ARHGEF19, TFF3, DSP | 786 | 80 | 16792 | 2.403435 | 1 | 0.63498 | 45.55999 |
| GOTERM_BP_DIRECT | GO:0042472~inner ear morphogenesis | 7 | 0.770925 | 0.033544 | FGF9, USH1G, ITGA8, PRRX1, CO | 786 | 52 | 16792 | 2.875905 | 1 | 0.636825 | 46.04413 |
| GOTERM_BP_DIRECT | GO:0048704~embryonic skeletal system morpho | 6 | 0.660793 | 0.034081 | MDFI, SATB2, OSR2, SOX11, WN | 786 | 39 | 16792 | 3.286749 | 1 | 0.63928 | 46.58355 |
| GOTERM_BP_DIRECT | GO:0043410~positive regulation of MAPK casca | 9 | 0.991189 | 0.035251 | BMP2, IL6, GPR37, FGF9, C1QTN | 786 | 81 | 16792 | 2.373763 | 1 | 0.648456 | 47.74116 |
| GOTERM_BP_DIRECT | GO:0055007~cardiac muscle cell differentiation | 5 | 0.550661 | 0.035348 | NOX4, BMP4, BMP2, MYOCD, G | 786 | 27 | 16792 | 3.956272 | 1 | 0.646073 | 47.83684 |
| GOTERM_BP_DIRECT | GO:0042476~odontogenesis | 5 | 0.550661 | 0.035348 | BMP4, OSR2, OSR1, AQP1, TWIS | 786 | 27 | 16792 | 3.956272 | 1 | 0.646073 | 47.83684 |
| GOTERM_BP_DIRECT | GO:0001656~metanephros development | 5 | 0.550661 | 0.035348 | OSR2, ID2, ITGA8, FOXC2, GLI3 | 786 | 27 | 16792 | 3.956272 | 1 | 0.646073 | 47.83684 |
| GOTERM_BP_DIRECT | GO:0045109~intermediate filament organizatio | 4 | 0.440529 | 0.036152 | DES, KRT17, DSP, KRT71 | 786 | 16 | 16792 | 5.340967 | 1 | 0.651103 | 48.61744 |
| GOTERM_BP_DIRECT | GO:0014002~astrocyte development | 4 | 0.440529 | 0.036152 | DRD1, LAMC3, DLL1, MT3 | 786 | 16 | 16792 | 5.340967 | 1 | 0.651103 | 48.61744 |
| GOTERM_BP_DIRECT | GO:0036149~phosphatidylinositol acyl-chain rer | 4 | 0.440529 | 0.036152 | PLA2G4A, PLA2G2A, PLA2G1B, F | 786 | 16 | 16792 | 5.340967 | 1 | 0.651103 | 48.61744 |
| GOTERM_BP_DIRECT | GO:0030819~positive regulation of cAMP biosyr | 6 | 0.660793 | 0.037507 | PTHLH, DRD1, ADRB1, ADORA2 | 786 | 40 | 16792 | 3.20458 | 1 | 0.661505 | 49.90741 |
| GOTERM_BP_DIRECT | GO:2000096~positive regulation of Wnt signalin | 3 | 0.330396 | 0.039192 | NKD1, GPC3, RSPO3 | 786 | 7 | 16792 | 9.155943 | 1 | 0.674602 | 51.47021 |
| GOTERM_BP_DIRECT | GO:0014049~positive regulation of glutamate se | 3 | 0.330396 | 0.039192 | ADORA2A, AVPR1A, NTSR1 | 786 | 7 | 16792 | 9.155943 | 1 | 0.674602 | 51.47021 |
| GOTERM_BP_DIRECT | GO:0060575~intestinal epithelial cell differentia | 3 | 0.330396 | 0.039192 | GATA5, GATA6, GATA4 | 786 | 7 | 16792 | 9.155943 | 1 | 0.674602 | 51.47021 |
| GOTERM_BP_DIRECT | GO:0010890~positive regulation of sequestering | 3 | 0.330396 | 0.039192 | LPL, PLIN5, FITM1 | 786 | 7 | 16792 | 9.155943 | 1 | 0.674602 | 51.47021 |
| GOTERM_BP_DIRECT | GO:0046069~cGMP catabolic process | 3 | 0.330396 | 0.039192 | PDE1B, PDE2A, PDE1A | 786 | 7 | 16792 | 9.155943 | 1 | 0.674602 | 51.47021 |
| GOTERM_BP_DIRECT | GO:0003183~mitral valve morphogenesis | 3 | 0.330396 | 0.039192 | EFNA1, SMAD6, TWIST1 | 786 | 7 | 16792 | 9.155943 | 1 | 0.674602 | 51.47021 |
| GOTERM_BP_DIRECT | GO:0006644~phospholipid metabolic process | 7 | 0.770925 | 0.039385 | LPL, PLA2G4A, GATA6, PLA2G2A | 786 | 54 | 16792 | 2.76939 | 1 | 0.673159 | 51.64642 |
| GOTERM_BP_DIRECT | GO:0018146~keratan sulfate biosynthetic proce | 5 | 0.550661 | 0.039766 | ST3GAL1, OMD, CHST6, ACAN, F | 786 | 28 | 16792 | 3.814976 | 1 | 0.673493 | 51.9913 |
| GOTERM_BP_DIRECT | GO:0048247~lymphocyte chemotaxis | 5 | 0.550661 | 0.039766 | CCL13, CCL23, CCL14, CCL16, CC | 786 | 28 | 16792 | 3.814976 | 1 | 0.673493 | 51.9913 |
| GOTERM_BP_DIRECT | GO:0019228~neuronal action potential | 5 | 0.550661 | 0.039766 | DRD1, P2RX1, CACNA1I, MYH14, | 786 | 28 | 16792 | 3.814976 | 1 | 0.673493 | 51.9913 |
| GOTERM_BP_DIRECT | GO:0040007~growth | 5 | 0.550661 | 0.039766 | INHBB, BMP4, BMP2, GDF6, FOX | 786 | 28 | 16792 | 3.814976 | 1 | 0.673493 | 51.9913 |
| GOTERM_BP_DIRECT | GO:0035690~cellular response to drug | 8 | 0.881057 | 0.041762 | EGFR, PDE2A, CRHBP, MT2A, KC | 786 | 69 | 16792 | 2.47697 | 1 | 0.688507 | 53.76424 |
| GOTERM_BP_DIRECT | GO:0030224~monocyte differentiation | 4 | 0.440529 | 0.042415 | BMP4, PDE1B, PDE2A, MT1G | 786 | 17 | 16792 | 5.026792 | 1 | 0.691099 | 54.33133 |
| GOTERM_BP_DIRECT | GO:0009948~anterior/posterior axis specificatio | 4 | 0.440529 | 0.042415 | BMP4, CDX1, GPC3, WLS | 786 | 17 | 16792 | 5.026792 | 1 | 0.691099 | 54.33133 |
| GOTERM_BP_DIRECT | GO:0032331~negative regulation of chondrocyt | 4 | 0.440529 | 0.042415 | PTHLH, BMP4, WNT9A, SNAI2 | 786 | 17 | 16792 | 5.026792 | 1 | 0.691099 | 54.33133 |
| GOTERM_BP_DIRECT | GO:0002076~osteoblast development | 4 | 0.440529 | 0.042415 | PTHLH, MSX2, SATB2, PTH1R | 786 | 17 | 16792 | 5.026792 | 1 | 0.691099 | 54.33133 |
| GOTERM_BP_DIRECT | GO:0010942~positive regulation of cell death | 5 | 0.550661 | 0.044482 | BMP4, CYSLTR2, HP, KCNK2, MT | 786 | 29 | 16792 | 3.683425 | 1 | 0.705557 | 56.08145 |
| GOTERM_BP_DIRECT | GO:0008544~epidermis development | 9 | 0.991189 | 0.044955 | PTHLH, COL17A1, KRT17, KRT5, | 786 | 85 | 16792 | 2.262057 | 1 | 0.706358 | 56.47244 |
| GOTERM_BP_DIRECT | GO:0002548~monocyte chemotaxis | 6 | 0.660793 | 0.04497 | CCL13, IL6, CCL23, CCL14, CCL16 | 786 | 42 | 16792 | 3.051981 | 1 | 0.703416 | 56.48477 |
| GOTERM_BP_DIRECT | GO:1903779~regulation of cardiac conduction | 7 | 0.770925 | 0.045834 | FXYD1, FXYD2, RYR3, PLN, NPR1 | 786 | 56 | 16792 | 2.670483 | 1 | 0.707397 | 57.19185 |
| GOTERM_BP_DIRECT | GO:0042733~embryonic digit morphogenesis | 7 | 0.770925 | 0.045834 | BMP4, OSR2, OSR1, GJA1, GLI3, | 786 | 56 | 16792 | 2.670483 | 1 | 0.707397 | 57.19185 |
| GOTERM_BP_DIRECT | GO:0042593~glucose homeostasis | 10 | 1.101322 | 0.046664 | SSTR5, IL6, FGFR4, SLC2A4, PPP1 | 786 | 101 | 16792 | 2.115234 | 1 | 0.710988 | 57.85979 |
| GOTERM_BP_DIRECT | GO:0071347~cellular response to interleukin-1 | 8 | 0.881057 | 0.047586 | CCL13, IL6, CCL23, CCL14, MYLK | 786 | 71 | 16792 | 2.407196 | 1 | 0.715198 | 58.59091 |
| GOTERM_BP_DIRECT | GO:0010596~negative regulation of endothelial | 4 | 0.440529 | 0.049182 | SERPINF1, APOH, DCN, SLIT2 | 786 | 18 | 16792 | 4.747526 | 1 | 0.724322 | 59.82815 |
| GOTERM_BP_DIRECT | GO:0036148~phosphatidylglycerol acyl-chain re | 4 | 0.440529 | 0.049182 | PLA2G4A, PLA2G2A, PLA2G1B, F | 786 | 18 | 16792 | 4.747526 | 1 | 0.724322 | 59.82815 |
| GOTERM_BP_DIRECT | GO:0007204~positive regulation of cytosolic calc | 12 | 1.321586 | 0.049491 | EDNRA, EDNRB, S1PR3, C1QTNF | 786 | 134 | 16792 | 1.913182 | 1 | 0.723701 | 60.06323 |
| GOTERM_BP_DIRECT | GO:0071773~cellular response to BMP stimulus | 5 | 0.550661 | 0.049498 | BMP4, BMP2, GATA5, GATA6, C | 786 | 30 | 16792 | 3.560645 | 1 | 0.720873 | 60.06869 |
| GOTERM_BP_DIRECT | GO:0007596~blood coagulation | 15 | 1.651982 | 0.050227 | ADORA2A, SCUBE1, F2RL1, ITGA | 786 | 184 | 16792 | 1.74162 | 1 | 0.72335 | 60.61856 |
| GOTERM_BP_DIRECT | GO:0070244~negative regulation of thymocyte a | 3 | 0.330396 | 0.050664 | BMP4, EFNA1, ADA | 786 | 8 | 16792 | 8.01145 | 1 | 0.723682 | 60.94536 |
| GOTERM_BP_DIRECT | GO:0019934~cGMP-mediated signaling | 3 | 0.330396 | 0.050664 | EDNRB, PDE2A, PRKG1 | 786 | 8 | 16792 | 8.01145 | 1 | 0.723682 | 60.94536 |
| GOTERM_BP_DIRECT | GO:0061303~cornea development in camera-typ | 3 | 0.330396 | 0.050664 | SOX11, WNT9B, WNT9A | 786 | 8 | 16792 | 8.01145 | 1 | 0.723682 | 60.94536 |
| GOTERM_BP_DIRECT | GO:0035865~cellular response to potassium ion | 3 | 0.330396 | 0.050664 | CYP11B2, CRHBP, CACNA1H | 786 | 8 | 16792 | 8.01145 | 1 | 0.723682 | 60.94536 |
| GOTERM_BP_DIRECT | GO:0050891~multicellular organismal water hor | 3 | 0.330396 | 0.050664 | SCNN1B, AQP1, SCNN1A | 786 | 8 | 16792 | 8.01145 | 1 | 0.723682 | 60.94536 |
| GOTERM_BP_DIRECT | GO:0048671~negative regulation of collateral sp | 3 | 0.330396 | 0.050664 | RGMA, BCL11A, FGF13 | 786 | 8 | 16792 | 8.01145 | 1 | 0.723682 | 60.94536 |
| GOTERM_BP_DIRECT | GO:0070588~calcium ion transmembrane transp | 11 | 1.211454 | 0.052071 | TMEM37, TRPM6, TRPC4, TRPM | 786 | 119 | 16792 | 1.974811 | 1 | 0.730843 | 61.97851 |
| GOTERM_BP_DIRECT | GO:0015701~bicarbonate transport | 6 | 0.660793 | 0.053257 | SLC26A4, SLC4A10, CA9, CA3, C | 786 | 44 | 16792 | 2.913255 | 1 | 0.736238 | 62.82996 |
| GOTERM_BP_DIRECT | GO:0007417~central nervous system developme | 11 | 1.211454 | 0.054543 | SH3GL3, NPAS2, HAPLN3, GABR | 786 | 120 | 16792 | 1.958355 | 1 | 0.742109 | 63.73239 |
| GOTERM_BP_DIRECT | GO:0045765~regulation of angiogenesis | 5 | 0.550661 | 0.054812 | TSPAN12, IL6, EFNA1, PROK1, V | 786 | 31 | 16792 | 3.445785 | 1 | 0.741175 | 63.91825 |
| GOTERM_BP_DIRECT | GO:0048701~embryonic cranial skeleton morph | 5 | 0.550661 | 0.054812 | BMP4, PRRX1, WNT9B, TBX1, TV | 786 | 31 | 16792 | 3.445785 | 1 | 0.741175 | 63.91825 |
| GOTERM_BP_DIRECT | GO:0051968~positive regulation of synaptic trar | 4 | 0.440529 | 0.05644 | EGFR, DRD1, ADORA2A, NLGN3 | 786 | 19 | 16792 | 4.497656 | 1 | 0.749006 | 65.02626 |
| GOTERM_BP_DIRECT | GO:0008015~blood circulation | 6 | 0.660793 | 0.057712 | COL4A3, MEOX2, ADORA2A, PLN | 786 | 45 | 16792 | 2.848516 | 1 | 0.75431 | 65.86849 |
| GOTERM_BP_DIRECT | GO:0048661~positive regulation of smooth mus | 7 | 0.770925 | 0.060598 | RBPMS2, BMP4, EGFR, IL6, ID2, I | 786 | 60 | 16792 | 2.492451 | 1 | 0.768931 | 67.71066 |
| GOTERM_BP_DIRECT | GO:0003151~outflow tract morphogenesis | 6 | 0.660793 | 0.062373 | BMP4, SOX11, TGFBR3, TBX1, W | 786 | 46 | 16792 | 2.786591 | 1 | 0.776442 | 68.79602 |
| GOTERM_BP_DIRECT | GO:0032000~positive regulation of fatty acid be | 3 | 0.330396 | 0.063164 | PLIN5, IRS1, TWIST1 | 786 | 9 | 16792 | 7.121289 | 1 | 0.778309 | 69.26852 |
| GOTERM_BP_DIRECT | GO:0071435~potassium ion export | 3 | 0.330396 | 0.063164 | KCND3, KCNA5, KCNQ1 | 786 | 9 | 16792 | 7.121289 | 1 | 0.778309 | 69.26852 |
| GOTERM_BP_DIRECT | GO:0006688~glycosphingolipid biosynthetic pro | 3 | 0.330396 | 0.063164 | A4GALT, ST8SIA5, ST8SIA1 | 786 | 9 | 16792 | 7.121289 | 1 | 0.778309 | 69.26852 |
| GOTERM_BP_DIRECT | GO:0086014~atrial cardiac muscle cell action po | 3 | 0.330396 | 0.063164 | GJA1, KCNA5, KCNQ1 | 786 | 9 | 16792 | 7.121289 | 1 | 0.778309 | 69.26852 |
| GOTERM_BP_DIRECT | GO:0050953~sensory perception of light stimulu | 3 | 0.330396 | 0.063164 | CLRN1, USH1G, CDH23 | 786 | 9 | 16792 | 7.121289 | 1 | 0.778309 | 69.26852 |
| GOTERM_BP_DIRECT | GO:0097105~presynaptic membrane assembly | 3 | 0.330396 | 0.063164 | PTPRD, NLGN3, LRP4 | 786 | 9 | 16792 | 7.121289 | 1 | 0.778309 | 69.26852 |
| GOTERM_BP_DIRECT | GO:0021795~cerebral cortex cell migration | 3 | 0.330396 | 0.063164 | EGFR, HTR6, FGF13 | 786 | 9 | 16792 | 7.121289 | 1 | 0.778309 | 69.26852 |
| GOTERM_BP_DIRECT | GO:0019370~leukotriene biosynthetic process | 4 | 0.440529 | 0.064174 | GGT5, GGT6, PLA2G1B, LTC4S | 786 | 20 | 16792 | 4.272774 | 1 | 0.781317 | 69.86255 |
| GOTERM_BP_DIRECT | GO:0030318~melanocyte differentiation | 4 | 0.440529 | 0.064174 | EDNRB, C10ORF11, GLI3, OCA2 | 786 | 20 | 16792 | 4.272774 | 1 | 0.781317 | 69.86255 |
| GOTERM_BP_DIRECT | GO:0030324~lung development | 8 | 0.881057 | 0.06432 | EGFR, ALDH1A2, GPC3, CCBE1, I | 786 | 76 | 16792 | 2.248828 | 1 | 0.779661 | 69.94751 |
| GOTERM_BP_DIRECT | GO:0055114~oxidation-reduction process | 37 | 4.07489 | 0.06561 | HSD3B2, PHYHD1, ALDH1L1, CYI | 786 | 592 | 16792 | 1.335242 | 1 | 0.784065 | 70.6877 |
| GOTERM_BP_DIRECT | GO:0009653~anatomical structure morphogene | 9 | 0.991189 | 0.065837 | GPC4, S1PR3, CPM, LYVE1, KRT1 | 786 | 92 | 16792 | 2.089944 | 1 | 0.782857 | 70.81622 |
| GOTERM_BP_DIRECT | GO:0015758~glucose transport | 5 | 0.550661 | 0.066321 | EDNRA, SLC2A4, SLC2A1, PLA2G | 786 | 33 | 16792 | 3.23695 | 1 | 0.782993 | 71.08844 |
| GOTERM_BP_DIRECT | GO:0010718~positive regulation of epithelial to | 5 | 0.550661 | 0.066321 | BMP2, ZNF703, LEF1, BAMBI, TV | 786 | 33 | 16792 | 3.23695 | 1 | 0.782993 | 71.08844 |
| GOTERM_BP_DIRECT | GO:0045600~positive regulation of fat cell differ | 6 | 0.660793 | 0.06724 | BMP2, RARRES2, ID2, WIF1, DKK | 786 | 47 | 16792 | 2.727302 | 1 | 0.785355 | 71.59891 |
| GOTERM_BP_DIRECT | GO:0016525~negative regulation of angiogenesi | 7 | 0.770925 | 0.068923 | COL4A3, SERPINF1, KLK3, APOH, | 786 | 62 | 16792 | 2.41205 | 1 | 0.791451 | 72.51156 |
| GOTERM_BP_DIRECT | GO:0045666~positive regulation of neuron diffe | 8 | 0.881057 | 0.071897 | BMP4, BMP2, DUOXA1, GDF6, S | 786 | 78 | 16792 | 2.191166 | 1 | 0.803352 | 74.05644 |
| GOTERM_BP_DIRECT | GO:0016125~sterol metabolic process | 4 | 0.440529 | 0.072367 | CYP7B1, CYP39A1, CYP21A2, CYI | 786 | 21 | 16792 | 4.069308 | 1 | 0.803286 | 74.29293 |
| GOTERM_BP_DIRECT | GO:0048844~artery morphogenesis | 4 | 0.440529 | 0.072367 | PRRX1, FOXC2, TBX1, WNT11 | 786 | 21 | 16792 | 4.069308 | 1 | 0.803286 | 74.29293 |
| GOTERM_BP_DIRECT | GO:0015718~monocarboxylic acid transport | 3 | 0.330396 | 0.076569 | SLC16A3, SLC16A9, SLC5A8 | 786 | 10 | 16792 | 6.40916 | 1 | 0.819568 | 76.31946 |
| GOTERM_BP_DIRECT | GO:0043586~tongue development | 3 | 0.330396 | 0.076569 | EGFR, LEF1, GLI3 | 786 | 10 | 16792 | 6.40916 | 1 | 0.819568 | 76.31946 |
| GOTERM_BP_DIRECT | GO:0010038~response to metal ion | 3 | 0.330396 | 0.076569 | MT1A, MT2A, MT3 | 786 | 10 | 16792 | 6.40916 | 1 | 0.819568 | 76.31946 |
| GOTERM_BP_DIRECT | GO:0051481~negative regulation of cytosolic cal | 3 | 0.330396 | 0.076569 | RYR3, KCNA5, HRC | 786 | 10 | 16792 | 6.40916 | 1 | 0.819568 | 76.31946 |
| GOTERM_BP_DIRECT | GO:0046415~urate metabolic process | 3 | 0.330396 | 0.076569 | GCKR, SLC16A9, SLC22A11 | 786 | 10 | 16792 | 6.40916 | 1 | 0.819568 | 76.31946 |
| GOTERM_BP_DIRECT | GO:0030574~collagen catabolic process | 7 | 0.770925 | 0.077877 | COL4A4, COL4A3, COL6A6, COL | 786 | 64 | 16792 | 2.336673 | 1 | 0.822881 | 76.91868 |
| GOTERM_BP_DIRECT | GO:0001890~placenta development | 5 | 0.550661 | 0.078979 | DLX3, NDP, MC2R, DCN, ADA | 786 | 35 | 16792 | 3.051981 | 1 | 0.825271 | 77.4126 |
| GOTERM_BP_DIRECT | GO:0006865~amino acid transport | 5 | 0.550661 | 0.078979 | SLC38A4, SLC16A10, SLC7A2, SL | 786 | 35 | 16792 | 3.051981 | 1 | 0.825271 | 77.4126 |
| GOTERM_BP_DIRECT | GO:0086091~regulation of heart rate by cardiac | 5 | 0.550661 | 0.078979 | KCND3, DSG2, DSP, KCNA5, KCN | 786 | 35 | 16792 | 3.051981 | 1 | 0.825271 | 77.4126 |
| GOTERM_BP_DIRECT | GO:0008285~negative regulation of cell prolifer | 26 | 2.863436 | 0.079434 | FRK, ADORA2A, PTH1R, FGFRL1, | 786 | 396 | 16792 | 1.402678 | 1 | 0.825027 | 77.61324 |
| GOTERM_BP_DIRECT | GO:0001558~regulation of cell growth | 8 | 0.881057 | 0.07998 | NOV, FXYD2, WISP2, RASGRP2, I | 786 | 80 | 16792 | 2.136387 | 1 | 0.825153 | 77.85222 |
| GOTERM_BP_DIRECT | GO:0043627~response to estrogen | 7 | 0.770925 | 0.082588 | GATA6, SMAD6, ESR1, CA2, GLI3 | 786 | 65 | 16792 | 2.300724 | 1 | 0.833242 | 78.96057 |
| GOTERM_BP_DIRECT | GO:0007189~adenylate cyclase-activating G-pro | 6 | 0.660793 | 0.083065 | PTHLH, DRD1, ADORA2A, ADOR | 786 | 50 | 16792 | 2.563664 | 1 | 0.833053 | 79.15746 |
| GOTERM_BP_DIRECT | GO:0030177~positive regulation of Wnt signalin | 5 | 0.550661 | 0.085727 | BMP2, DACT1, RSPO1, RSPO3, W | 786 | 36 | 16792 | 2.967204 | 1 | 0.840852 | 80.22485 |

| | | | | | | | | | | | | |
|---|---|---|---|---|---|---|---|---|---|---|---|---|
| GOTERM_BP_DIRECT | GO:0007200~phospholipase C-activating G-prot | 7 | 0.770925 | 0.087454 | EDNRB, GPR139, NMUR1, CYSLT | 786 | 66 | 16792 | 2.265865 | 1 | 0.845016 | 80.88972 |
| GOTERM_BP_DIRECT | GO:0007507~heart development | 14 | 1.54185 | 0.088023 | DNAH11, BMP2, FOXL1, GJA1, T | 786 | 183 | 16792 | 1.634394 | 1 | 0.845096 | 81.10396 |
| GOTERM_BP_DIRECT | GO:0007519~skeletal muscle tissue developmer | 6 | 0.660793 | 0.088741 | MEOX2, MYL3, KLHL31, MYH14, | 786 | 51 | 16792 | 2.513396 | 1 | 0.845684 | 81.37117 |
| GOTERM_BP_DIRECT | GO:0042542~response to hydrogen peroxide | 6 | 0.660793 | 0.088741 | TRPA1, HP, KCNA5, AREG, ADA, | 786 | 51 | 16792 | 2.513396 | 1 | 0.845684 | 81.37117 |
| GOTERM_BP_DIRECT | GO:0007219~Notch signaling pathway | 10 | 1.101322 | 0.08966 | S1PR3, BMP2, CNTN6, PLN, FOX( | 786 | 115 | 16792 | 1.857728 | 1 | 0.846942 | 81.70808 |
| GOTERM_BP_DIRECT | GO:0045766~positive regulation of angiogenesis | 10 | 1.101322 | 0.08966 | GATA6, CYSLTR2, CCBE1, GATA4 | 786 | 115 | 16792 | 1.857728 | 1 | 0.846942 | 81.70808 |
| GOTERM_BP_DIRECT | GO:0007166~cell surface receptor signaling patl | 19 | 2.092511 | 0.089796 | EGFR, COL4A3, IL1R1, CTF1, PTH | 786 | 274 | 16792 | 1.481436 | 1 | 0.845561 | 81.75748 |
| GOTERM_BP_DIRECT | GO:0032967~positive regulation of collagen bios | 4 | 0.440529 | 0.090052 | BMP4, WNT4, CREB3L1, ITGA2 | 786 | 23 | 16792 | 3.715455 | 1 | 0.844588 | 81.84996 |
| GOTERM_BP_DIRECT | GO:0006702~androgen biosynthetic process | 3 | 0.330396 | 0.090769 | HSD3B2, WNT4, SCARB1 | 786 | 11 | 16792 | 5.826509 | 1 | 0.84517 | 82.10695 |
| GOTERM_BP_DIRECT | GO:0001946~lymphangiogenesis | 3 | 0.330396 | 0.090769 | CCBE1, PTPN14, FOXC2 | 786 | 11 | 16792 | 5.826509 | 1 | 0.84517 | 82.10695 |
| GOTERM_BP_DIRECT | GO:0048484~enteric nervous system developme | 3 | 0.330396 | 0.090769 | EDNRA, EDNRB, SOX8 | 786 | 11 | 16792 | 5.826509 | 1 | 0.84517 | 82.10695 |
| GOTERM_BP_DIRECT | GO:0048745~smooth muscle tissue developmer | 3 | 0.330396 | 0.090769 | BMP4, ITGA8, FOXP2 | 786 | 11 | 16792 | 5.826509 | 1 | 0.84517 | 82.10695 |
| GOTERM_BP_DIRECT | GO:0042417~dopamine metabolic process | 3 | 0.330396 | 0.090769 | DRD1, SNCAIP, NPR1 | 786 | 11 | 16792 | 5.826509 | 1 | 0.84517 | 82.10695 |
| GOTERM_BP_DIRECT | GO:0030517~negative regulation of axon extens | 3 | 0.330396 | 0.090769 | BCL11A, RTN4R, MT3 | 786 | 11 | 16792 | 5.826509 | 1 | 0.84517 | 82.10695 |
| GOTERM_BP_DIRECT | GO:0006707~cholesterol catabolic process | 3 | 0.330396 | 0.090769 | CYP39A1, SCARB1, MT3 | 786 | 11 | 16792 | 5.826509 | 1 | 0.84517 | 82.10695 |
| GOTERM_BP_DIRECT | GO:0001823~mesonephros development | 3 | 0.330396 | 0.090769 | BMP4, OSR2, OSR1 | 786 | 11 | 16792 | 5.826509 | 1 | 0.84517 | 82.10695 |
| GOTERM_BP_DIRECT | GO:0061045~negative regulation of wound heal | 3 | 0.330396 | 0.090769 | WNT4, GJA1, WFDC1 | 786 | 11 | 16792 | 5.826509 | 1 | 0.84517 | 82.10695 |
| GOTERM_BP_DIRECT | GO:0003323~type B pancreatic cell developmen | 3 | 0.330396 | 0.090769 | BMP4, SMO, DLL1 | 786 | 11 | 16792 | 5.826509 | 1 | 0.84517 | 82.10695 |
| GOTERM_BP_DIRECT | GO:0098911~regulation of ventricular cardiac m | 3 | 0.330396 | 0.090769 | DSG2, DSP, RYR2 | 786 | 11 | 16792 | 5.826509 | 1 | 0.84517 | 82.10695 |
| GOTERM_BP_DIRECT | GO:2000296~negative regulation of hydrogen p | 2 | 0.220264 | 0.091314 | HP, MT3 | 786 | 2 | 16792 | 21.36387 | 1 | 0.845172 | 82.29983 |
| GOTERM_BP_DIRECT | GO:0035051~cardiocyte differentiation | 2 | 0.220264 | 0.091314 | BMP2, MYOCD | 786 | 2 | 16792 | 21.36387 | 1 | 0.845172 | 82.29983 |
| GOTERM_BP_DIRECT | GO:0038183~bile acid signaling pathway | 2 | 0.220264 | 0.091314 | VDR, NR1H4 | 786 | 2 | 16792 | 21.36387 | 1 | 0.845172 | 82.29983 |
| GOTERM_BP_DIRECT | GO:1901894~regulation of calcium-transporting | 2 | 0.220264 | 0.091314 | SLN, PLN | 786 | 2 | 16792 | 21.36387 | 1 | 0.845172 | 82.29983 |
| GOTERM_BP_DIRECT | GO:1901877~negative regulation of calcium ion | 2 | 0.220264 | 0.091314 | SLN, PLN | 786 | 2 | 16792 | 21.36387 | 1 | 0.845172 | 82.29983 |
| GOTERM_BP_DIRECT | GO:0072174~metanephric tubule formation | 2 | 0.220264 | 0.091314 | WNT4, WNT9B | 786 | 2 | 16792 | 21.36387 | 1 | 0.845172 | 82.29983 |
| GOTERM_BP_DIRECT | GO:2000609~regulation of thyroid hormone ger | 2 | 0.220264 | 0.091314 | DUOXA2, DUOXA1 | 786 | 2 | 16792 | 21.36387 | 1 | 0.845172 | 82.29983 |
| GOTERM_BP_DIRECT | GO:0072180~mesonephric duct morphogenesis | 2 | 0.220264 | 0.091314 | GPC3, OSR1 | 786 | 2 | 16792 | 21.36387 | 1 | 0.845172 | 82.29983 |
| GOTERM_BP_DIRECT | GO:0090427~activation of meiosis | 2 | 0.220264 | 0.091314 | MSX2, MSX1 | 786 | 2 | 16792 | 21.36387 | 1 | 0.845172 | 82.29983 |
| GOTERM_BP_DIRECT | GO:2001055~positive regulation of mesenchyma | 2 | 0.220264 | 0.091314 | MSX2, MSX1 | 786 | 2 | 16792 | 21.36387 | 1 | 0.845172 | 82.29983 |
| GOTERM_BP_DIRECT | GO:0071422~succinate transmembrane transpc | 2 | 0.220264 | 0.091314 | SLC13A5, SLC13A3 | 786 | 2 | 16792 | 21.36387 | 1 | 0.845172 | 82.29983 |
| GOTERM_BP_DIRECT | GO:0086100~endothelin receptor signaling path | 2 | 0.220264 | 0.091314 | EDNRA, EDNRB | 786 | 2 | 16792 | 21.36387 | 1 | 0.845172 | 82.29983 |
| GOTERM_BP_DIRECT | GO:0032912~negative regulation of transformin | 2 | 0.220264 | 0.091314 | GATA6, CDH3 | 786 | 2 | 16792 | 21.36387 | 1 | 0.845172 | 82.29983 |
| GOTERM_BP_DIRECT | GO:0002017~regulation of blood volume by ren | 2 | 0.220264 | 0.091314 | CYP11B2, HSD11B2 | 786 | 2 | 16792 | 21.36387 | 1 | 0.845172 | 82.29983 |
| GOTERM_BP_DIRECT | GO:0007588~excretion | 5 | 0.550661 | 0.092744 | ADORA2B, KCNK5, CLCNKA, CLC | 786 | 37 | 16792 | 2.887009 | 1 | 0.848073 | 82.79693 |
| GOTERM_BP_DIRECT | GO:0045786~negative regulation of cell cycle | 5 | 0.550661 | 0.092744 | BMP4, BMP2, NUPR1, INHA, HP( | 786 | 37 | 16792 | 2.887009 | 1 | 0.848073 | 82.79693 |
| GOTERM_BP_DIRECT | GO:0071320~cellular response to cAMP | 6 | 0.660793 | 0.094613 | NOX4, ASS1, CRHBP, AQP1, KCN | 786 | 52 | 16792 | 2.465062 | 1 | 0.852273 | 83.42647 |
| GOTERM_BP_DIRECT | GO:0051897~positive regulation of protein kina: | 8 | 0.881057 | 0.097656 | NOX4, EGFR, TNFAIP8L3, IL6, MI | 786 | 84 | 16792 | 2.034654 | 1 | 0.85985 | 84.40548 |
| GOTERM_BP_DIRECT | GO:0030511~positive regulation of transforming | 4 | 0.440529 | 0.099505 | MYOCD, ITGA8, NPNT, TGFBR3 | 786 | 24 | 16792 | 3.560645 | 1 | 0.863583 | 84.97322 |
