# Supplementary material for: Identification of Clinical Relevant Molecular Subtypes of Pheochromocytoma
Source: Front Endocrinol (Lausanne). 2021 Jun 21;12:605797. doi: 10.3389/fendo.2021.605797 (PMC8256389; doi:10.3389/fendo.2021.605797)
Supplement: Supplementary file 4 [file DataSheet_4.pdf]

Table 4. Biological process enriched in each subtypes of PCC. Enriched GO BP terms by genes over-expressed in Subtype I PCC.

Enriched GO BP ter	Term	Count	%	PValue	Genes	List Total	Pop Hits	Pop Total	Fold Enrich
KEGG_PATHWAY	hsa04080:Neuroactive ligand-receptor interaction	33	0.028361	1.80E-09	OPRM1, GRIK1, GLRA1, DRD2, NPY2R, DR	243	277	6879	3.37251
KEGG_PATHWAY	hsa04728:Dopaminergic synapse	19	0.016329	4.33E-07	KIF5A, DRD2, DRD5, TH, MAOB, GRIA3, C	243	128	6879	4.202064
KEGG_PATHWAY	hsa05033:Nicotine addiction	9	0.007735	6.05E-05	SLC32A1, GABRG2, GABRG3, GRIA2, GRIA	243	40	6879	6.369444
KEGG_PATHWAY	hsa05031:Amphetamine addiction	11	0.009454	8.97E-05	ARC, CAMK4, GRIA2, GRIA1, PPP1R1B, M	243	66	6879	4.718107
KEGG_PATHWAY	hsa05034:Alcoholism	17	0.01461	4.68E-04	HIST1H2AG, DRD2, HIST1H2BG, TH, MAO	243	177	6879	2.718909
KEGG_PATHWAY	hsa04723:Retrograde endocannabinoid signaling	12	0.010313	7.88E-04	GNG8, KCNJ5, SLC32A1, GABRG2, GABRG	243	101	6879	3.363403
KEGG_PATHWAY	hsa05032:Morphine addiction	11	0.009454	0.001266	OPRM1, GNG8, KCNJ5, SLC32A1, GABRG2	243	91	6879	3.421924
KEGG_PATHWAY	hsa04020:Calcium signaling pathway	16	0.013751	0.001527	SLC8A2, DRD5, LHCGR, ATP2B2, CHRM5,	243	179	6879	2.530381
KEGG_PATHWAY	hsa04024:cAMP signaling pathway	17	0.01461	0.001572	DRD2, DRD5, ATP1A3, CREB5, GRIA3, CN	243	198	6879	2.43054
KEGG_PATHWAY	hsa04725:Cholinergic synapse	12	0.010313	0.001723	GNG8, CHRM5, KCNQ3, CHRM4, CAMK4,	243	111	6879	3.060394
KEGG_PATHWAY	hsa05030:Cocaine addiction	7	0.006016	0.007014	GRIA2, DRD2, PPP1R1B, MAOB, TH, CREB	243	49	6879	4.044092
KEGG_PATHWAY	hsa04514:Cell adhesion molecules (CAMs)	12	0.010313	0.011198	ALCAM, NCAM2, CADM1, NRXN3, CD274	243	142	6879	2.39228
KEGG_PATHWAY	hsa00601:Glycosphingolipid biosynthesis - lacto and neolacto se	5	0.004297	0.01214	FUT9, B3GALT5, B3GNT4, B3GALT1, B3G	243	26	6879	5.44397
KEGG_PATHWAY	hsa04360:Axon guidance	11	0.009454	0.013725	PLXNC1, EPHA6, EPHA8, PAK3, PLXNB1, S	243	127	6879	2.45193
KEGG_PATHWAY	hsa04713:Circadian entrainment	9	0.007735	0.018253	GNG8, KCNJ5, NOS1AP, GRIA2, GRIA1, RY	243	95	6879	2.681871
KEGG_PATHWAY	hsa04730:Long-term depression	7	0.006016	0.018255	IGF1R, GRIA2, GRIA1, GRID2, RYR1,	243	60	6879	3.302675
KEGG_PATHWAY	hsa04724:Glutamatergic synapse	10	0.008594	0.018643	GLS2, GNG8, GRIA2, GRIK1, GRIA1, GRIA3	243	114	6879	2.483214
KEGG_PATHWAY	hsa04261:Adrenergic signaling in cardiomyocytes	11	0.009454	0.023253	ATP2B2, ATP2B3, MAPK13, ATP1A3, CRE	243	138	6879	2.256486
KEGG_PATHWAY	hsa04727:GABAergic synapse	8	0.006875	0.029472	GLS2, GNG8, SLC32A1, GABRG2, GABRG3	243	85	6879	2.664343
KEGG_PATHWAY	hsa05014:Amyotrophic lateral sclerosis (ALS)	6	0.005156	0.030241	PRPH, GRIA2, GRIA1, MAPK13, NEFL, NEF	243	50	6879	3.397037
KEGG_PATHWAY	hsa04911:Insulin secretion	7	0.006016	0.078072	GIP, ATP1A3, CREB5, CAMK2B, GLP1R,	243	85	6879	2.3313
KEGG_PATHWAY	hsa04530:Tight junction	7	0.006016	0.085196	MYH15, CLDN10, CLDN20, AMOTL1, PRK	243	87	6879	2.277707
KEGG_PATHWAY	hsa04726:Serotonergic synapse	8	0.006875	0.095567	GNG8, KCNJ5, HTR7, MAOB, GNB3, CACN	243	111	6879	2.040262

Biological process enriched in each subtype of PCC. Enriched GO BP terms by genes over-expressed in Subtype II PCC.

Category	Term	Count	%	PValue	Genes	List Total	Pop Hits	Pop Total	Fold Enrich
KEGG_PATHWAY	hsa04080:Neuroactive ligand-receptor interaction	33	3.650442	2.11E-06	DRD1, THRB, ADORA2B, ADORA2A,	327	277	6879	2.506177
KEGG_PATHWAY	hsa04020:Calcium signaling pathway	25	2.765487	3.24E-06	EGFR, GNA14, DRD1, ADORA2B, TNNC1,	327	179	6879	2.938086
KEGG_PATHWAY	hsa04024:cAMP signaling pathway	24	2.654867	5.55E-05	FXYD2, PLD1, DRD1, VAV3, ADORA2A, NP	327	198	6879	2.549903
KEGG_PATHWAY	hsa04974:Protein digestion and absorption	15	1.659292	5.79E-05	COL4A4, COL4A3, FXYD2, COL13A1, SLC1	327	88	6879	3.585801
KEGG_PATHWAY	hsa04270:Vascular smooth muscle contraction	17	1.880531	1.15E-04	RAMP3, ADORA2B, ADORA2A, MYLK3, PR	327	117	6879	3.056614
KEGG_PATHWAY	hsa05217:Basal cell carcinoma	11	1.216814	1.85E-04	FZD9, SMO, WNT4, BMP2, FZD10, WNT9	327	54	6879	4.285253
KEGG_PATHWAY	hsa04390:Hippo signaling pathway	19	2.10177	2.59E-04	FZD9, BMP2, GDF6, SOX2, WWC1, LEF1, T	327	151	6879	2.647002
KEGG_PATHWAY	hsa04978:Mineral absorption	9	0.995575	9.26E-04	FXYD2, TF, VDR, TRPM6, MT1M, MT1A, M	327	44	6879	4.302961
KEGG_PATHWAY	hsa05414:Dilated cardiomyopathy	11	1.216814	0.006062	TNNT2, DES, ADRB1, MYL3, TNNC1, ITGA	327	84	6879	2.754806
KEGG_PATHWAY	hsa04022:cGMP-PKG signaling pathway	16	1.769912	0.007723	FXYD2, MYLK3, NPR1, PRKG1, IRS1,	327	158	6879	2.130298
KEGG_PATHWAY	hsa05032:Morphine addiction	11	1.216814	0.010547	GABRD, GABRE, DRD1, GNGT2, GABRR1,	327	91	6879	2.542897
KEGG_PATHWAY	hsa05410:Hypertrophic cardiomyopathy (HCM)	10	1.106195	0.011016	TNNT2, DES, MYL3, TNNC1, ITGA8, ITGA7	327	78	6879	2.697012
KEGG_PATHWAY	hsa05412:Arrhythmogenic right ventricular cardiomyopathy (AR	9	0.995575	0.013285	DES, DSG2, ITGA8, ITGA7, LEF1, GJA1, DS	327	67	6879	2.825825
KEGG_PATHWAY	hsa04512:ECM-receptor interaction	10	1.106195	0.021418	COL4A4, COL4A3, COL6A6, LAMC3, COL2	327	87	6879	2.418011
KEGG_PATHWAY	hsa00603:Glycosphingolipid biosynthesis - globo series	4	0.442478	0.026007	ST3GAL1, A4GALT, ST8SIA1, FUT1	327	14	6879	6.010485
KEGG_PATHWAY	hsa04261:Adrenergic signaling in cardiomyocytes	13	1.438053	0.030187	FXYD2, MYL3, TNNC1, MYH6, TPM2, TNN	327	138	6879	1.981718
KEGG_PATHWAY	hsa04340:Hedgehog signaling pathway	5	0.553097	0.036711	SMO, HHIP, GAS1, GLI2, GLI1	327	27	6879	3.895685
KEGG_PATHWAY	hsa04925:Aldosterone synthesis and secretion	9	0.995575	0.037193	HSD3B2, PDE2A, CYP21A2, CYP11B2, CAC	327	81	6879	2.337411
KEGG_PATHWAY	hsa04151:PI3K-Akt signaling pathway	25	2.765487	0.037261	COL4A4, PHLPP1, EGFR, COL4A3, FGF9, F	327	345	6879	1.524398
KEGG_PATHWAY	hsa05033:Nicotine addiction	6	0.663717	0.03914	SLC17A7, GABRD, GABRE, GABRR1,	327	40	6879	3.155505
KEGG_PATHWAY	hsa05200:Pathways in cancer	27	2.986726	0.051568	FGF9, FGF11, FGF10, FGF13, BDKRB1, GN	327	393	6879	1.445269
KEGG_PATHWAY	hsa00140:Steroid hormone biosynthesis	7	0.774336	0.055373	HSD3B2, CYP7B1, CYP3A5, CYP11B1, CYP	327	58	6879	2.538912
KEGG_PATHWAY	hsa04961:Endocrine and other factor-regulated calcium reabsor	6	0.663717	0.060142	FXYD2, VDR, KLK2, PTH1R, ESR1, KLK1	327	45	6879	2.804893
KEGG_PATHWAY	hsa04260:Cardiac muscle contraction	8	0.884956	0.063747	TNNT2, FXYD2, MYL3, TNNC1, COX4I2,	327	75	6879	2.243914
KEGG_PATHWAY	hsa04713:Circadian entrainment	9	0.995575	0.080325	GNGT2, GRIN2C, RYR3, CACNA1I, CACNA	327	95	6879	1.99295
KEGG_PATHWAY	hsa04924:Renin secretion	7	0.774336	0.081386	EDNRA, ADRB3, ADRB1, PDE1B, PDE1A, N	327	64	6879	2.300889
KEGG_PATHWAY	hsa00250:Alanine, aspartate and glutamate metabolism	5	0.553097	0.081829	FOLH1, ASPA, ASS1, GAD1, DDO	327	35	6879	3.005242
KEGG_PATHWAY	hsa04066:HIF-1 signaling pathway	9	0.995575	0.084217	EGFR, TF, SLC2A1, HK2, EGF, CAMK2A,	327	96	6879	1.97219
KEGG_PATHWAY	hsa04964:Proximal tubule bicarbonate reclamation	4	0.442478	0.092748	FXYD2, CA2, AQP1, PCK1	327	23	6879	3.658556

Bonferron	Benjamin	FDR
3.91E-07	3.91E-07	2.28E-06
9.43E-05	4.72E-05	5.50E-04
0.013109	0.004389	0.076909
0.019375	0.004879	0.114013
0.097019	0.020204	0.593265
0.15789	0.028234	0.996951
0.241312	0.038684	1.597307
0.283261	0.040776	1.923109
0.290288	0.037383	1.979437
0.313349	0.036895	2.168038
0.78444	0.130208	8.55856
0.914138	0.185014	13.33688
0.930242	0.18521	14.3801
0.950846	0.19362	16.11005
0.981975	0.234888	20.87616
0.981983	0.221998	20.87814
0.98347	0.214417	21.27469
0.994077	0.247942	25.84761
0.998529	0.290531	31.63049
0.998762	0.28446	32.31599
1	0.569946	64.41415
1	0.586193	67.75554
1	0.614055	72.10568

Bonferron	Benjamin	FDR
4.86E-04	4.86E-04	0.00271
7.45E-04	3.73E-04	0.004155
0.012675	0.004243	0.071079
0.013226	0.003323	0.07419
0.026005	0.005256	0.146765
0.04161	0.007058	0.236619
0.05793	0.008489	0.332086
0.19198	0.026294	1.181193
0.753006	0.143908	7.498719
0.831904	0.163327	9.461818
0.912734	0.198851	12.71057
0.921736	0.191282	13.23867
0.953855	0.210703	15.75638
0.993123	0.299309	24.23773
0.997667	0.332392	28.66873
0.999133	0.356368	32.49554
0.999816	0.397116	38.09151
0.999836	0.383874	38.4869
0.999839	0.36851	38.54282
0.999897	0.368187	40.06334
0.999995	0.440035	49.27669
0.999998	0.44874	51.82459
0.999999	0.462201	54.85145
1	0.468072	57.02161
1	0.537154	65.81959
1	0.528081	66.32186
1	0.516757	66.52909
1	0.514542	67.62854
1	0.537896	71.28847
